# A new theropod dinosaur from the early cretaceous (Barremian) of Cabo Espichel, Portugal: Implications for spinosaurid evolution

**DOI:** 10.1371/journal.pone.0262614

**Published:** 2022-02-16

**Authors:** Octávio Mateus, Darío Estraviz-López

**Affiliations:** 1 GEOBIOTEC, Department of Earth Sciences, NOVA School of Science and Technology, Caparica, Portugal; 2 Museu da Lourinhã, Lourinhã, Portugal; Chinese Academy of Sciences, CHINA

## Abstract

Spinosaurids are some of the most enigmatic Mesozoic theropod dinosaurs due to their unique adaptations to aquatic environments and their relative scarcity. Their taxonomy has proven to be especially problematic. Recent discoveries from Western Europe in general, specifically Iberia, provide some of the best specimens for the understanding of their phylogeny, leading to the description of the spinosaurid *Vallibonavenatrix cani* and the recognition of the Iberian dinosaur *Camarillasaurus cirugedae* as one of them. Portuguese associated spinosaurid remains (ML1190) from the Papo Seco Formation (early Barremian) were previously assigned to *Baryonyx walkeri* but new material recovered in 2020 along with new phylogenetic analyses suggests a different phylogenetic placement, making their revision necessary. Here we show that these remains are not attributable to *Baryonyx walkeri*, but to a new genus and species, *Iberospinus natarioi*, gen. et sp. nov. The new taxon is characterized by the presence of a single Meckelian foramen in the Meckelian sulcus, a straight profile of the ventral surface of the dentary and a distal thickening of the acromion process of the pubis between other characters. *Iberospinus natarioi* is recovered as a sister taxon of the clade formed by *Baryonyx* and *Suchomimus*, and outside Spinosaurinae when *Vallibonaventrix cani* is excluded from the analysis. The description of this taxon reinforces Iberia as a hotspot for spinosaur biodiversity, with several endemic taxa for the region. As expected for the clade, the dentary displays a highly vascularized neurovascular network. The morphometric analysis of parts of the skeleton (pedal phalanx and caudal vertebrae, among others) shows an intermediate condition between basal tetanurans and spinosaurines.

## Introduction

### The spinosaurid theropods

Spinosauridae Stromer, 1915 [1] is a bizarre clade of theropods at several levels [2]. Phylogenies point towards a ghost lineage for them of approximately 50 million years, between the middle Jurassic and the early Cretaceous [2] during which spinosaurid material is scarce or disputed; possible spinosaurid specimens from the Jurassic includes isolated teeth from the Tiourarén Formation of Niger and the Tendaguru beds of Tanzania as well as a manual ungual phalanx previously attributed to *Torvosaurus*, from the Morrison Formation of Colorado. However, these specimens are referred to as non-spinosaurid theropods by other authors [2–4]. The oldest uncontroversial spinosaurids come from the Barremian of Western Europe [5–7] and Southeast Asia [8], later appearing in the Aptian of Southeast Asia [9] and Africa [10] and the Albian of South America [11], with their last confirmed occurrences in the Cenomanian of North Africa and South America [2], although there is a possible occurrence in the Santonian of East Asia [12]. They are not as well-known as other theropod groups due to a combination of the fragmentary nature of their fossil record, mainly based on isolated elements like teeth [13] and their divergent bauplan from other theropods, either in cranial morphology or at a more general level in their body plan [14, 15]. This is related with their ecology, particularly linked to aquatic environments, either as specialized pursuit aquatic predators in some cases or as wading-ambush hunters, like herons [16, 17]. Given some neuroanatomical adaptations and other evidence, their diet mainly included fish [18], although other food items (e.g., pterosaurs) were consumed [19]. Isotopic studies have shown some variability in their habits and diet, with some spinosaurids presenting values of δ18O similar to those of semiaquatic animals like crocodylomorphs [20].

Typically, they were recovered as the sister taxa of Megalosauridae Fitzinger, 1843 [21]; united within the clade Megalosauroidea (Fitzinger, 1843) [21] by the following characters: the shape of the jugal ramus of the postorbital, the step to the hyposphene in dorsal vertebrae and the tall neural spines [22]. But a recent work [23] has suggested that they might actually not be part of a monophyletic Megalosauroidea, but a distinct clade of their own, within Tetanurae Gauthier, 1986 [24]. The taxonomy of the group itself is a highly contested matter, given the paucity of the fossil record and the non-overlapping nature of most of the taxa, with the taxonomy of North African spinosaurids particularly controversial [25–27]. Traditionally Spinosauridae has been divided into Baryonychinae Charig & Milner, 1986 [5] and Spinosaurinae Stromer, 1915 [1].While they recover a similar topology, recent studies point towards the possible paraphyletic nature of the subfamily Baryonychinae [11, 25]. The use of Bayesian statistics and different matrices have recovered a scenario in which the baryonychines still form a monophyletic group, although its taxonomy remains unsettled [7].

Spinosauridae has been defined as “The most inclusive clade containing *Spinosaurus aegyptiacus* but not *Torvosaurus tanneri*, *Allosaurus fragilis*, and *Passer domesticus*” [28] or “All theropods that are more closely related to *Spinosaurus aegyptiacus* than to either *Megalosaurus bucklandii* or *Allosaurus fragilis*” [23].

Spinosaurids are characterized by their robust forelimbs, tall neural spines and elongated jaws. Spinosauridae can be diagnosed by the following characters: presence of a premaxillary rosette, external nares posterior to the premaxillary teeth [10], ventral keel in posterior cervicals and anterior dorsals, pneumaticity or webbing at the base of the neural spine of the middle and posterior dorsals [25], and 21 dental characters including a dentary rosette with four teeth, flutes in the teeth and roots strongly tapered apically and oval to subcircular in cross-section at mid-root [29]. Different synapomorphies have been suggested in other analyses, such as the presence of accessory centrodiapophyseal laminae in middle to posterior dorsal vertebrae, the elongation of dorsal spines (at least twice the height of the centrum), a deep infraprezygapophyseal fossa, and six to seven premaxillary teeth (more than in any other tetanuran) [2, 25, 30].

#### Historical review of spinosaurid dinosaurs from Iberia

In spite of not being recognized as such, Spinosauridae has been known in Portugal since the 19th century thanks to the discovery of “*Suchosaurus girardi*’’ (Sauvage, 1897–1898) [31–33]. Shortly after the discovery of *Baryonyx*, a series of papers referred several isolated elements to this taxon from Spain, including a left maxilla [34] and a partial vertebral column and forelimb [35]. In addition, some isolated teeth and a hypertrophied manual ungual phalanx were attributed to baryonychine theropods [36, 37] along with several isolated teeth referred to spinosaurines [38, 39]. The first taxon of Spinosauridae described from the Iberian Peninsula, *Vallibonavenatrix cani* Malafaia et al., 2020 [6], was established on the basis of cervical, dorsal, sacral, and caudal elements, with fragments of ribs, chevrons, and a relatively complete pelvic girdle; their analysis placed it within Spinosaurinae. *Camarillasaurus cirugedae* Sánchez-Hernández & Benton, 2012 [40] was originally regarded as a ceratosaur but has now been suggested to be a megalosauroid, with probable spinosaurid affinities [8, 41]. For a more extended overview of Iberian Spinosauridae, as well as a detailed catalog of material, see [30].

Specimen ML1190, the subject of this work, was first described in 2011 after its discovery by the amateur fossil collector Carlos Natário in 1999. It was originally referred by Mateus et al., 2011 [42] to *Baryonyx walkeri* on the basis of the following dental characteristics: “Enamel surface with small (and nearly vertical) wrinkles, variable denticle size along the carinae, 6–7 denticles per millimeter, wrinkles forming a 45 degree angle near the carinae, and tooth root longer than crown”, although another autapomorphy of *Baryonyx* according to Sereno et al., 1998 [10], a well-developed peg-and-notch scapular attachment to the coracoid, is also mentioned. This work also notes some differences with the holotype of *Baryonyx*, especially the mound-like eminence on the lateral surface of the proximal pubis [42].

ML1190 was subjected to another study in 2017, when Waskow & Mateus studied histological samples from a rib attributed to the fossil. Their main conclusion regarding ML1190 was that this rib belonged to a fully mature individual of about 23–25 years of age when it died. It reached sexual maturity at about 13–15 years of age, despite the lack of fusion of the dorsal neural arches with the centrum by the time of its death [43].

More recently, ML1190 was included in a phylogenetic analysis by Arden et al., 2019 [44], in a matrix derived from that of Evers et al., 2015 [25]. This analysis recovered the specimen outside of the clade formed by *Baryonyx* or *Suchomimus*, due to the lack of the upturned anterior part of the dentary present in both aforementioned taxa.

In 2021, ML1190 was included in yet another two phylogenetic analyses. The first was a parsimony analysis that recovered the specimen as an indeterminate Baryonychinae and the second was a Bayesian analysis that found it to be grouped together with *Baryonyx* [7].

### Objectives of this work

Given the doubt cast on the original attribution raised by Arden et al., 2019 [44], and the parsimony analysis performed in [7], this work aims to clarify the phylogenetic position of ML1190. The second objective is to redescribe the published material and to describe new fossils, discovered during an excavation at the Praia de Aguncheiras site in June of 2020. The third objective will be to contribute to the knowledge of morphofunctional questions related to the paleobiology of spinosaurid theropods and a quantitative analysis of several elements (dentary, teeth, pedal ungual phalanx and caudal vertebrae) will be carried out. Finally, the last objective will be to study the neurovascular system and teeth replacement pattern of the dentary of ML1190 using CT scan data.

## Material and methods

### Geographical, stratigraphical and paleoenvironmental settings

**Fig 1:**
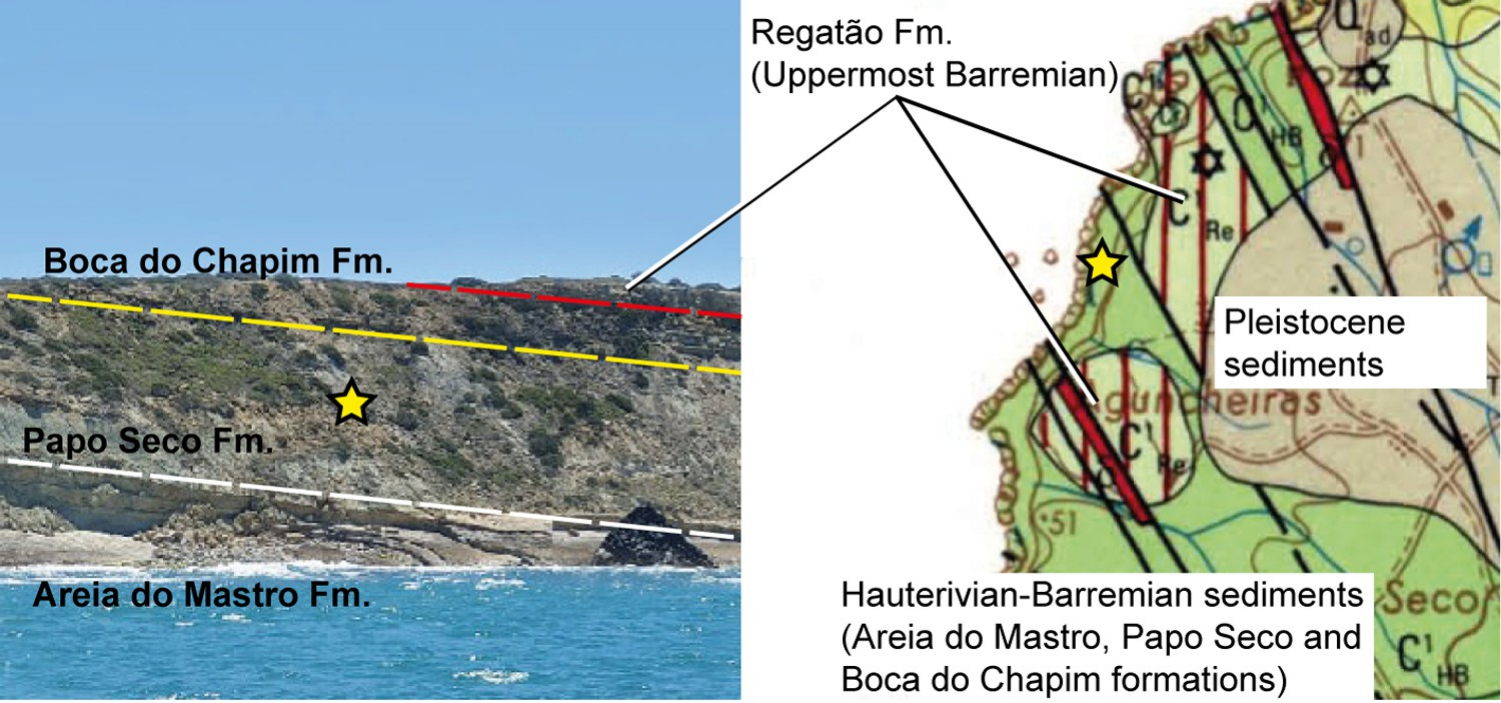
Geographical and geological settings of the type locality of *Iberospinus natarioi* (ML1190) at Praia de Aguncheiras. **Left**, photograph from the sea, showing the immediate geological context of the site (courtesy of André Carvalho); **right**, Geological map of the local area and Cretaceous outcrops (adapted from Manuppella, 1999). The site is marked with a yellow star. **White line**, base of Papo Seco formation; **yellow line**, base of Boca do Chapim formation; **red line**, base of Regatão formation.

The Papo Seco Formation outcrops are located in Cabo Espichel (Espichel Cape) in the Setúbal Peninsula, district of the same name, Municipality of Sesimbra, central west Portugal (38.4° N, 9.2° W; paleocoordinates: 31.8° N, 1.3° E, Fig 1). The Papo Seco Formation was first named by Rey [45], it is also known as *Grès marneux à Grands Sauriens* by Choffat [46] and *Grès à Dinosauriens* [47]. It was named after the site of Barraca do Papo Seco, 1500 m NW from Azóia. It is underlain by the Areia do Mastro Formation and overlain by the Boca do Chapim Formation [48].

Exposure of the Papo Seco Formation is found between the sites of Rochadouro and Areia do Mastro, above the low cliff along the foreshore and beach at Boca do Chapim. The Papo Seco Formation marks a return to more clastic-dominated sedimentation after the more carbonated Areia do Mastro Formation, primarily dominated by silty mud-mud deposition and green silty clays, with lignitic horizons and gypsum. It is interbedded with medium to coarse, commonly ribbon shaped, clastic sandstones. Along the exposure, between Rochadouro and Boca do Chapim, the clastic sands are not laterally continuous. The exposure clearly shows that the ribbon sands are isolated and change laterally into muds. Three distinct sand facies have been identified within the Papo Seco Formation [45, 49]. According to Rey et al., 2003 and Dinis et al., 2008 [50, 51], the Papo Seco Formation is Barremian in age.

The environment in which the Papo Seco Formation was deposited was more continental than in the overlying and underlying formations, being mostly lagoonal, with continental influences and a relatively closed environment with restricted marine influences, as freshwater bivalves of the genus *Nipponomaia* are also known [52, 53]. Nevertheless, some marine gastropods of the family Naticidae [52] have been documented, meaning that the conditions were likely partially estuarine.

Ganoid scales and teeth of *Lepidotes*-like Holostei fishes are common in the area [52, 53]. Tetrapod remains include Plesiochelyidae testudines, represented by multiple remains [52, 53], goniopholid teeth similar to those of *Anteophtalmosuchus* [54], and pterosaur teeth belonging to the clades Ornithocheiridae and Ctenochamatoidea [54]. Also discovered from the same area of Praia de Aguncheiras (also known as Praia do Guincho) is a possible ornithopod track from the Papo Seco Formation [55] although the immense majority of the tracks (from sauropod, ornithopod, and theropod dinosaurs) recovered in the Early Cretaceous of the area of Espichel come from the underlying and more carbonated Areia do Mastro Formation [56].

Body remains of ornithopod dinosaurs known from the area are mainly comprised of teeth, although a distal femur and fragmented caudal vertebrae have been mentioned by several works [54, 57, 58]. Part of a maxilla [54] from an ornithopod dinosaur was also published. Recently, associated ornithopod material coming from the Boca do Chapim locality was reported, comprising “several vertebrae and chevrons, a rib, pelvic bones, and a phalanx” [59].

Sauropod dinosaurs are known from teeth previously ascribed to *Astrodon* or *Pleurocoelus*, although they are most likely too fragmentary to be assigned with any certainty to any taxon [57, 58, 60]. More recently, part of a caudal vertebra was described from the lowermost part of the Papo Seco Formation [54].

Theropod dinosaurs are known from several spinosaurid teeth, besides those previously mentioned [54]. Also, a large bodied non-spinosaurid theropod is known due to two tooth fragments of a ziphodont tooth [58, 60]. A partial ulna from a dromaeosaurid or avian dinosaur was also recently recovered from the upper part of the Areia do Mastro Formation [61].

### Material

**Fig 2:**
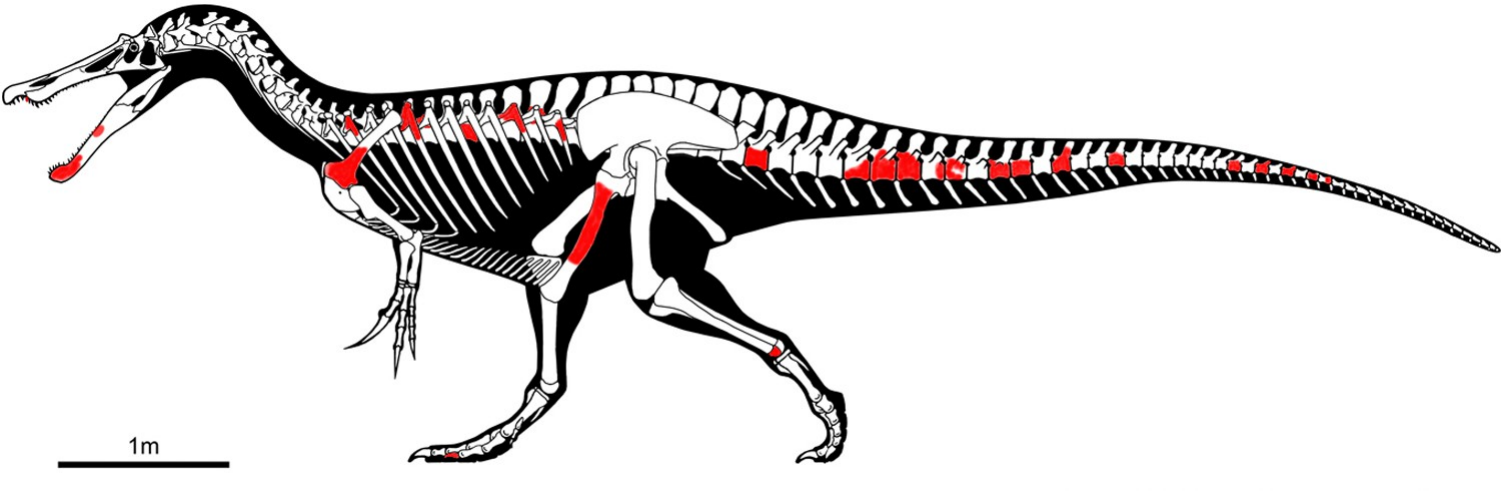
Skeletal diagram showing in red the recovered elements of ML1190 (*Iberospinus natarioi*) at Praia de Aguncheiras. Courtesy of Scott Hartman.

ML1190 is housed at the Museum of Lourinhã (ML), situated in the village of Lourinhã, Lisbon district, Portugal. The revision of already known material plus the inclusion of undescribed elements and new materials recovered during the excavation of June 2020 results in the following elements: one partial left dentary, one fragment of dentary including at least four teeth, one isolated complete tooth, two fragmentary isolated teeth, partial right scapula, one dorsal vertebral centrum, two damaged dorsal neural arches, four proximal rib fragments, a left pubic peduncle, right pubic shaft, two partial calcanea, one pedal ungual phalanx, and fifteen caudal vertebrae in different states of preservation. The list of this material, including inventory numbers, is provided in Table 1, we also present a graphical diagram of the recovered material (Fig 2). We are confident that all of this material comes from the same specimen as: 1) all the remains come from a small area of 5 x 2 meters, 2) they are of the same size class and, 3) there are no duplicate elements that could point to multiple specimens. Numerous parts of zygapophyses, ribs and probably chevrons as well as the remains of ornithopods, turtles, pterosaurs, and fish have also been recovered at the site. Furthermore, several gastropods and fossil plant material (they will be subject of another upcoming study) were also collected.

**Table 1:** Material recovered from the holotype of *Iberospinus natarioi* (ML1190).

Catalog number	Element
ML1190-1	Anterior part of dentary
ML1190-2	Fragment of dentary
ML1190-3	Tooth
ML1190-4	Fragmentary tooth
ML1190-5	Fragmentary tooth
ML1190-10	Partial right scapula
ML1190-6/8	Dorsal neural arch
ML1190-7	Dorsal neural arch
ML1190-234	Dorsal vertebra
ML1190-11	Proximal rib fragment
ML1190-12	Proximal rib fragment
ML1190-28	Proximal rib fragment
ML1190-239	Proximal rib fragment
ML1190-14	Pubic peduncle
ML1190-13	Right pubis shaft
ML1190-31	Calcaneum
ML1190-32	Partial calcaneum
ML1190-34	Ungual pedal phalanx
ML1190-15	Anterior caudal vertebra
ML1190-17	Anterior caudal vertebra
ML1190-16	Anterior caudal vertebra
ML1190-22	Anterior caudal vertebra
ML1190-18	Mid-anterior caudal vertebra
ML1190-21	Fragmentary middle caudal vertebra
ML1190-20	Middle caudal vertebra (broken in half)
ML1190-26	Middle caudal vertebra
ML1190-23	Mid-posterior caudal vertebra
ML1190-19	Posterior caudal vertebra
ML1190-27	Posterior caudal vertebra
ML1190-25	Posterior caudal vertebra
ML1190-240	Posterior caudal vertebra
ML1190-241	Posterior caudal vertebra
ML1190-275	Half posterior caudal vertebra

### Methods

All necessary permits were obtained for the described study, in compliance with all relevant regulations. The ICNF ("Instituto para a conservação e as Florestas") from Portugal granted permission for the fieldwork in the Natural Park of Serra da Arrábida, Portugal, in June of 2020 that resulted in the material herein published. Permit number: OF.24364/2020/DR-LVT/DRCNB/DOT.

First, each of the relevant materials is described in detail, including measurements taken with digital calipers. We used the terminology proposed by Hendrickx et al., 2015 [62] for the description of teeth. Both ML1190-1 and ML1190-2 were CT scanned in a GE VtomeX M240 machine at Microsense, Metrologia Industrial Lda. in Leiria, Portugal; capable of producing 0.05 mm slices, with resolution dependent on the size of the fossil. Avizo 9 was used in order to segment the obtained data and Blender was used for the rendering. Special care was given to the description of the inner cavities of the dentary, the neurovascular system and, the teeth replacement pattern. Also, most of the pieces were surface scanned in order to obtain non-distorted images of the fossils, with the images being rendered with the software Blender as well. The measurements of the dentary ML1190-1 were compared with those taken with the program ImageJ from photos of the holotype of *Baryonyx* (NHMUK VP R9951) and a dentary attributed to *Spinosaurus* (NHMUK VP 16421). The measurements of the caudal vertebrae were used to assess their order in the caudal series, using as a template the tail of the specimen FSAC-KK 11888, attributed to *Spinosaurus*, the most complete tail of any spinosaurid dinosaur known [16]. Likewise their measurements and proportions were compared with those of a theropod with a more universal body plan, *Dilophosaurus wetherilli*, as measured by the most recent work [63], as well as other large theropods with sufficiently well-known tails [64]. A similar methodology was used to quantitatively compare the ungual phalanx with other known theropod unguals, especially those included in [17]. The measurements, taken in accordance with [65], of the complete and isolated tooth (ML1190-3) were inserted into a database with more than 80 spinosaurid teeth measurements, from at least eight taxa of the group in order to perform a Principal Component Analysis (PCA). These measurements proposed in [62, 65] were compared with a range of other teeth (S4 File) attributed to basal spinosaurids [4], Iberian baryonychines [66], *Baryonyx* and *Suchomimus* [62, 65], Iberian spinosaurines [67, 68], *Irritator* [62], and *Spinosaurus* [62, 69]. For the first analysis (Fig 27A), a PCA was carried out; in order to include the biggest sample of teeth possible the apical length (AL) measurement according to [65] was not included.

We included original coding of *Vallibonavenatrix* from [6] and coded the additional characters from Arden et al., 2019 [44] that were possible to code for *Vallibonaventrix*. Then we coded into this matrix (which will serve as the basis of our analysis) 18 extra characters for ML1190, as well as re-coding character 148, as the premaxillary teeth are not known with certainty in the specimen. As ML1190 comprised mandibular and dental material, we coded its 36 observable characters into the matrix of Hendrickx et al., 2020 [70] the most comprehensive matrix for dental characters in theropods. Then we merged this matrix with the matrix from [44]. We deleted the paleobiogeographic and temporal characters matrix as we considered that Arden et al., 2019 [44] placed too much importance into the place and time of origin for each specimen (animals that lived in Europe shared one character even if they had nothing else in common). As both matrices had inherited characters from [71] we deleted two redundant characters from the matrix of [44] that we considered better detailed in the analysis from [70] (characters 153 and 31 in [71]) and vice versa with another character (character 14 of [70]). We finally deleted all the taxa from the matrix of [70] not included into the one of [44] and vice versa. The genera remaining in the analysis (besides the Iberian specimens *Vallibonavenatrix* and ML1190) are the spinosaurids: *Baryonyx*, *Suchomimus*, *Irritator*, *Ichthyovenator*, and *Spinosaurus*. The resulting matrix included 24 taxa, 534 characters, and was analyzed using TNT 1.5 (No taxon limit) [44]. See (S1 & S2 Files) for further details about the phylogenetic analyses.

#### Nomenclatural acts

The electronic edition of this article conforms to the requirements of the amended International Code of Zoological Nomenclature (ICZN), and hence the new names contained herein are available under that Code from the electronic edition of this article. This published work and the nomenclatural acts it contains have been registered in ZooBank, the online registration system for the ICZN. The ZooBank LSIDs (Life Science Identifiers) can be resolved and the associated information viewed through any standard web browser by appending the LSID to the prefix “http://zoobank.org/”. The LSID for this publication is: urn:lsid:zoobank.org:pub:69B14608-20E0-4B38-9A52-6016199EB5FB. The electronic edition of this work was published in a journal with an ISSN, has been archived, and is available from the following digital repositories: PubMed Central, LOCKSS.

## Results

### Systematic paleontology

Dinosauria Owen, 1842 [72]

Theropoda Marsh, 1881 [73]

Megalosauroidea (Fitzinger, 1843) [21]

Spinosauridae Stromer, 1915 [1]

*Iberospinus* n. gen.

urn:lsid:zoobank.org:act:7641902A-10D4-42E6-8C49-F4EA6DE2DC95

Etymology: *Ibero*- derived from the Roman name for the Iberian Peninsula; and -*spinus*, latin for “spine”, because the length of the neural spines is one of the main features that defined the clade to which this animal belongs to.

Diagnosis: As for the type species (see below).

*Iberospinus natarioi* sp. nov.

urn:lsid:zoobank.org:act:4D94DAA9-7E3B-4D4A-9507-487B02B6740D

Etymology: Dedicated to Carlos Natário, who discovered the holotype.

Holotype: ML 1190, many bones from same individual (Table 1).Type locality and horizon: Praia de Aguncheiras (Also known as Praia do Guincho), in the municipality of Sesimbra, Setúbal district, Portugal (38.4° N, 9.2° W; paleocoordinates: 31.8° N, 1.3° E). The sediments that contain the fossil belong to the Papo Seco Formation, dated to Early Barremian [50, 51].

Diagnosis: Medium sized spinosaurid theropod with the following autapomorphies: 1) dentary with a single foramen in the Meckelian sulcus, 2) dentary´s ventral edge is straight (not upturned), 3) presence of laminae in the pleurocelic depression of the medio-distal caudal vertebrae, 4) scapula, anterior rim is straight (acromion not protruding), 5) scapula with reduced acromial ridge, 6) scapula, contact with coracoid occupies the entire ventral surface, 7) pubic apron thick in almost the entire length of the pubis shaft, 8) mound like eminence in the proximal lateral part of the pubis.

### Description and comparisons of ML1190

#### Anterior part of left dentary (ML1190-1)

**Fig 3:**
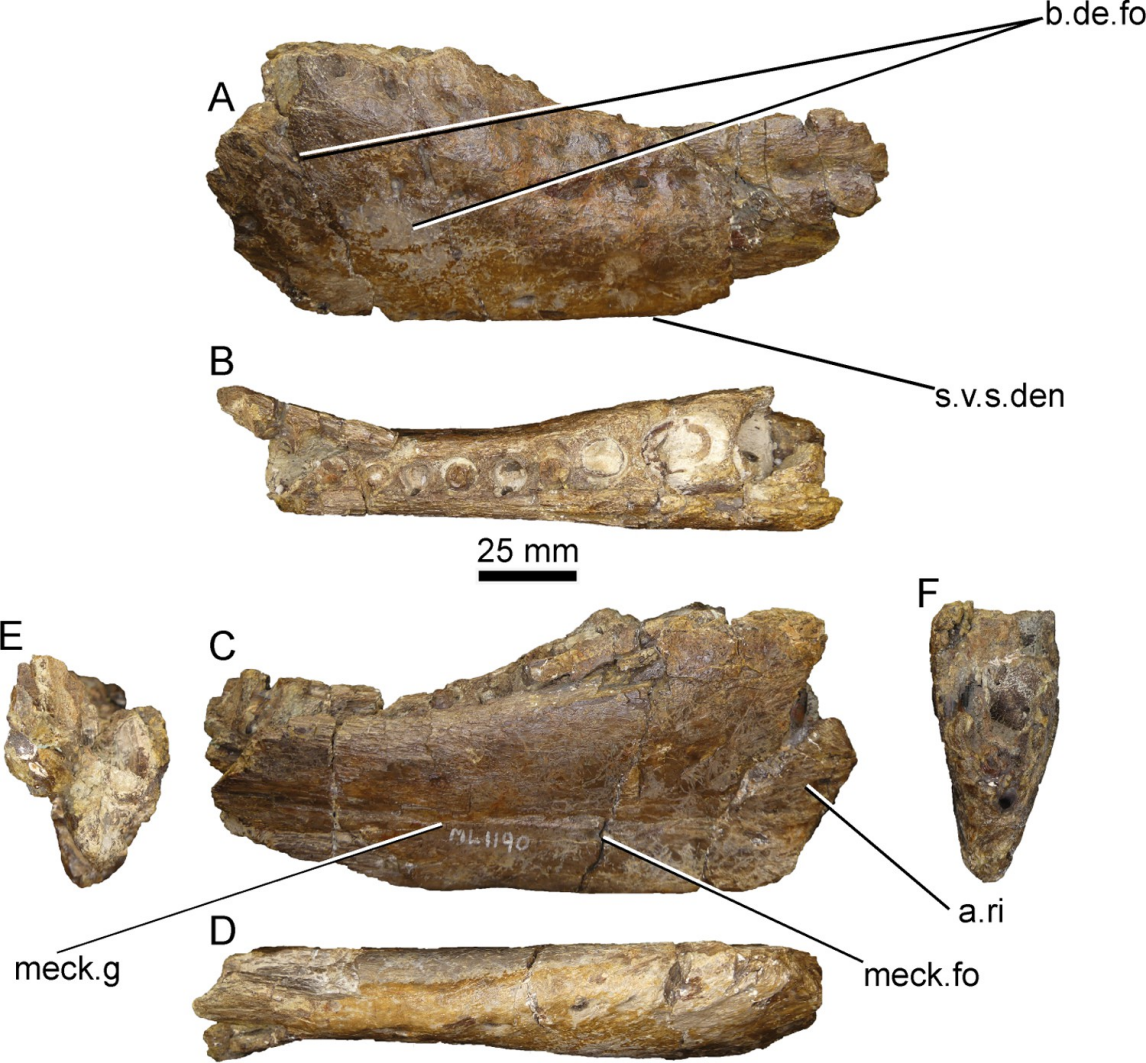
Anterior left dentary portion (holotype) of *Iberospinus natarioi* (ML1190-1). **A, lateral, B, dorsal, C, medial, D, ventral, E, posterior, and F anterior views. a.ri**, anterior ridges, **b.d.fo**, lateral dentary foramina, **meck.g**, Meckelian groove, **meck.fo**, Meckelian foramen, **s.v.s.den**, straight ventral surface of the dentary.

This piece was already described by Mateus et al., 2011 [42] and corresponds to the anteriormost part of the left dentary, with 12 alveoli present, although only nine are well preserved. Its measurements can be found in the S3 File. The anterior part is eroded, and the wall of the first preserved socket is missing, exposing three foramina in this area (Fig 3). In spite of this:

1. The symphyseal ridges are present in the ventro-medial side of the dentary, as noted by Hendrickx et al., 2016 [26] and they extend for about 10 mm. They do not extend more than ~25 mm from the anterior edge of the dentary in the holotype of *Baryonyx*.
2. The ventral Meckelian foramen is situated about 70 mm from the tip of the dentary in the holotype of *Baryonyx*, and lingually to the fourth alveoli. It is situated 50 mm caudally from the breaking point of the dentary in ML-1190 and medially to the third preserved foramen.
3. In the dorsal view, the lateral and medial sides of the dentary are converging anteriorly and are only separated by 15 mm at the point of breakage.

The dentary is most likely missing one tooth socket from the tip and that it is mostly complete in its anterior part. Therefore, we counted the number of alveoli from this missing alveolus.

In anterior view, the dentary is sub-triangular in shape with the medial surface straight and the lateral surface more expanded. A narrow sulcus corresponding to the paradental groove is present between the 4th and 6th alveoli. The interdental plates are heavily worn and it is difficult to distinguish their shape, although the one between the 4th and 5th is sub-triangular in shape. As described by Mateus et al., 2011 [42], replacement teeth can be seen in the 3rd, 7th and 9th alveoli. In the 7th and 8th it is also possible to distinguish the nutrient foramina.

**Fig 4:**
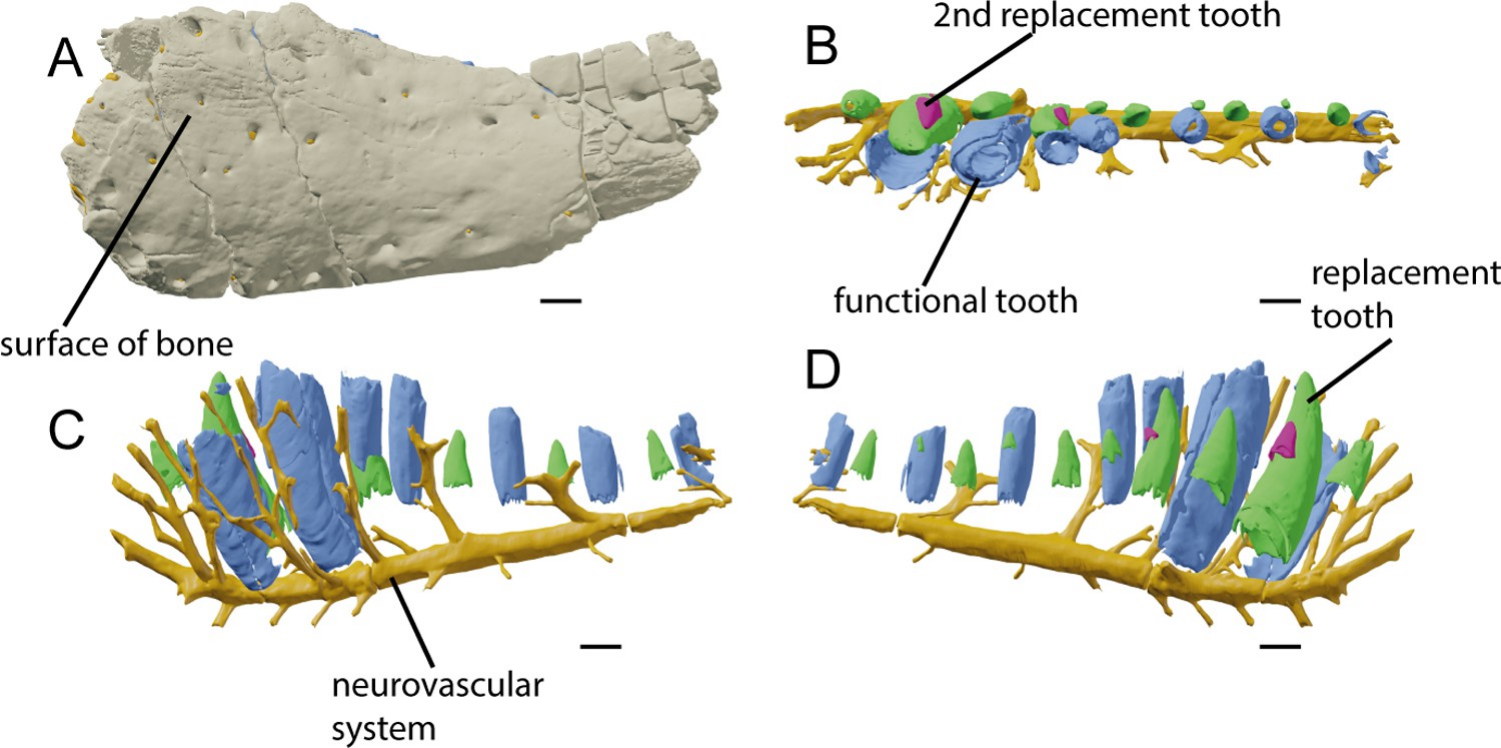
CT-scan of the holotype of *Iberospinus* natarioi (ML1190-1). **A**, Surface of the bone and the foramina in lateral view; **B**, Neurovascular system, teeth and replacement teeth in occlusal view; **C**, Neurovascular system, teeth and replacement teeth in lateral view; **D**, Neurovascular system, teeth and replacement teeth in occlusal view. Scale bar is 10 mm. Voxel size of the model: 0.100628. **Grey**, dentary bone, **Yellow**, neurovascular system, **Blue**, teeth, **Green**, first replacement teeth, **Pink**, second replacement teeth.

The Meckelian sulcus that runs through the medial side of the dentary is shallow compared to the one in the left dentary of the holotype of *Baryonyx*, and much straighter, running parallel to the ventral margin of the dentary. As confirmed by the CT-scan, the dorsal Meckelian foramen is absent (Fig 4), an autapomorphic situation not seen in any other megalosauroid [22]. The lateral side bears 28 foramina. The conserved 90 mm ventral surface of the dentary is straight in contrast with the strongly upturned NHM R9951 as pointed out by Arden et al., 2019 [44].

**Fig 5:**
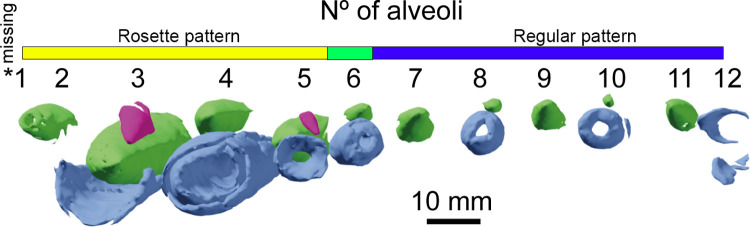
CT-scan of the holotype of *Iberospinus natarioi* (ML1190-1), showing the erupted teeth and replacement teeth. Scale bar is 10 mm. Voxel size of the model: 0.100628. **Blue**, erupted or semi-erupted teeth, **Green**, first replacement teeth, **Fuchsia**, second replacement teeth.

For the internal anatomy of the dentary, we have first to cover the replacement teeth and their pattern of eruption (Fig 5). There are two different areas and patterns of eruption; the first five alveoli (of them only the three distal ones are well preserved) bear either one or two replacement teeth in each of them, as seen in the premaxilla of *Oxalaia quilombensis* [74] and in the maxilla of the megalosauroid, *Torvosaururus gurneyi* [75]. The distal alveoli (from the 6th alveoli backwards) bear a pattern where the even number alveoli (i.e., 6, 8, 10) have small replacement teeth, meanwhile the odd ones bear a single and big replacement tooth, with the functional teeth being lost most likely due to taphonomic processes [7, 9, 11].

**Fig 6:**
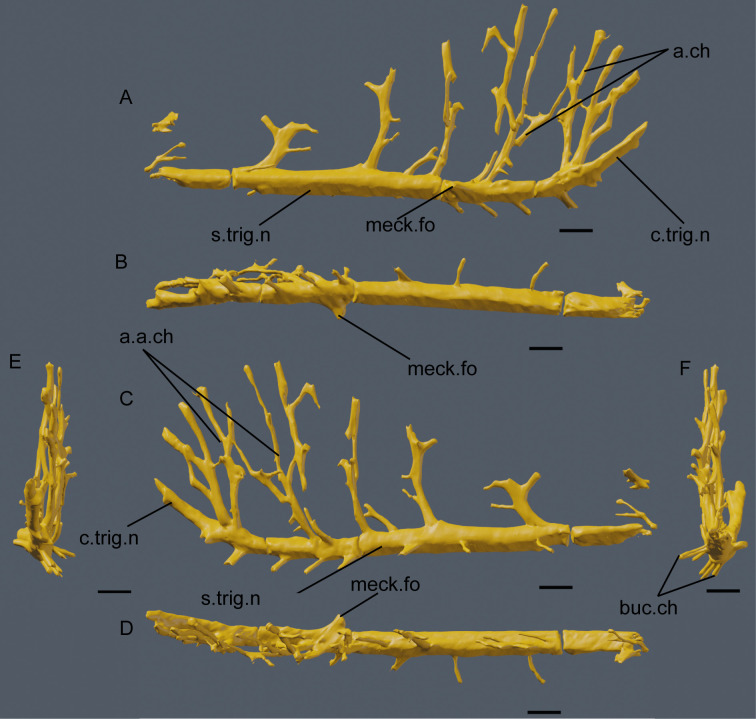
CT-scan of the holotype of *Iberospinus* natarioi (ML1190-1), showing the neurovascular system of the dentary. **A**, medial, **B**, ventral, **C**, lateral, **D**, occlusal, **E**, anterior, and **F** posterior view. **a.a.ch**, anterior anastomosed channels, **buc.ch**, lateral channels, **c.trig.n**, curved part of the mandibular branch of the trigeminal nerve, **meck.fo**, branch of the Meckelian foramen, **s.trig.n**, straight part of the mandibular branch of the trigeminal nerve. Scale bar is 10 mm. Voxel size of the model: 0.100628.

In the neurovascular system, there are two main groups of foramina projecting to the exterior along with one isolated foramen, all of them diverging from the main neurovascular canal. This is interpreted as the mandibular ramus of the trigeminal nerve (Fig 6). The trigeminal nerve itself curves dorsally, anteriorly from the 3rd alveoli, with it dividing in two just below the missing first and not preserved alveoli. The first and anteriormost group of foramina are eight simple and straight channels that project lateroventrally from the trigeminal nerve; posteriorly they become longer, thinner and more laterally oriented. Given that in the anteriormost portion the trigeminal nerve points dorsally, the first of this group actually points anteriorly from the dentary. The isolated foramen is the ventral Meckelian foramen, that points medially and slightly posteriorly from the fourth tooth socket. It is the only neurovascular canal in the preserved length of the dentary that clearly points medially and opens to the exterior, being short and wide compared with others in the dentary. The second group is the most complex in structure. It is composed of canals projecting dorsally from the main canal, anastomosed and with openings both to the alveoli and to the lateral surface of the dentary. There are eight main branches, which subsequently divide, with the most complex structures being situated anteriorly to the 4th alveoli. In this area the vascularization is so intense that channels form a braided structure connecting the 6th to the 8th rami in a net of neurovascular passages.

#### Piece of dentary (ML1190-2)

**Fig 7:**
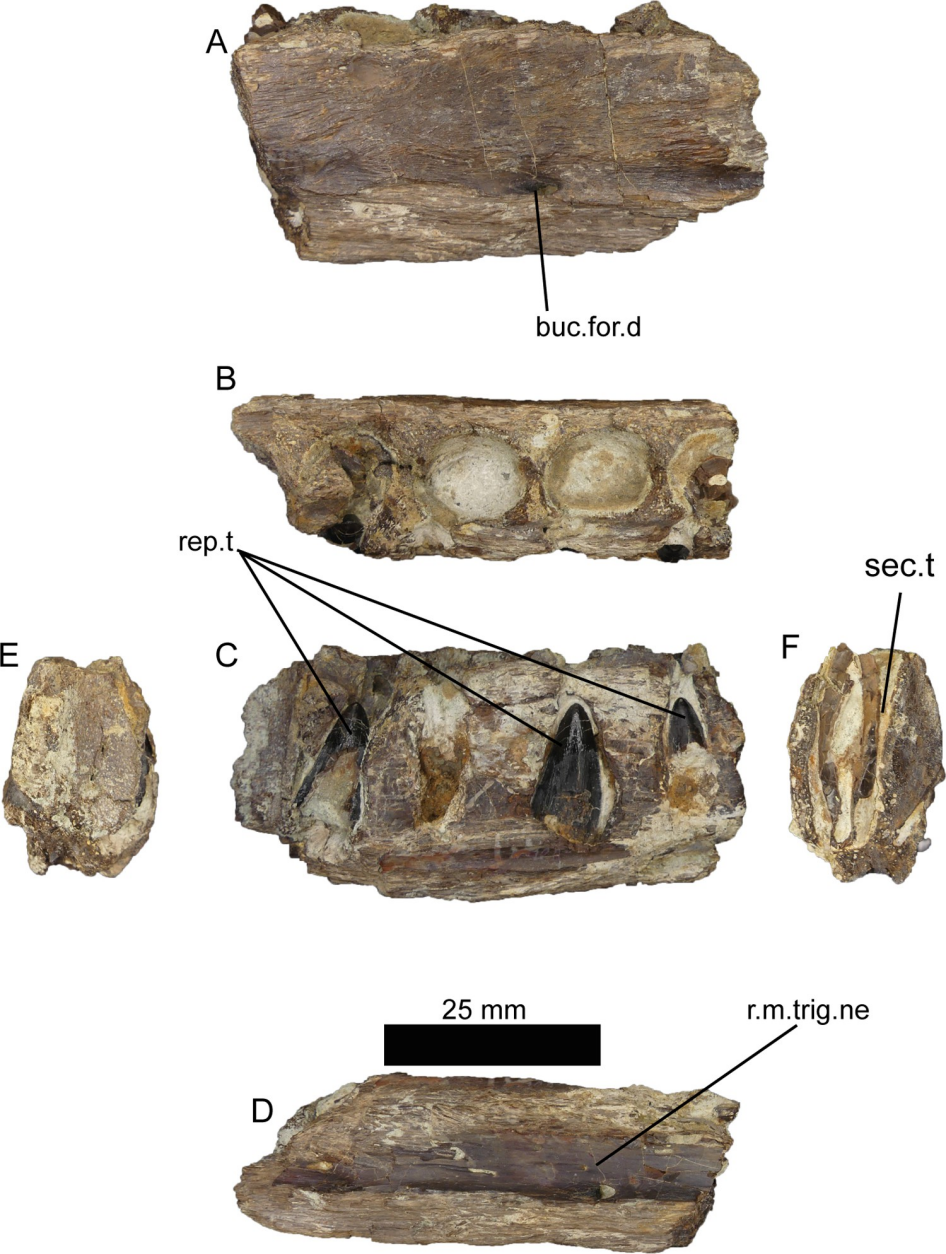
Dentary fragment of *Iberospinus natarioi* (ML1190-2). **A**, lateral, **B**, occlusal, **C**, medial, **D**, ventral, **E**, posterior, and **F** anterior view. **for.d**, medial foramen in the dentary, **rep.t.**, replacement teeth, **r.trg.n**, roof of the mandibular ramus of the trigeminal nerve **sec.t**, sectioned tooth.

This small piece of dentary with four alveoli was briefly mentioned by Mateus et al., 2011 [42]. The first and last tooth sockets bear the remnants of two teeth still in place, one in each socket. Three secondary replacement teeth are in the medial side of the fragment, two of them in the same alveoli as the previously aforementioned. It is still possible to see the remains of the paradental groove. In ventral view, the roof of a neurovascular channel is visible, likely the dorsal part of the mandibular branch of the trigeminal nerve. Therefore, this fossil could be interpreted as a dentary piece (Fig 7).

**Fig 8:**
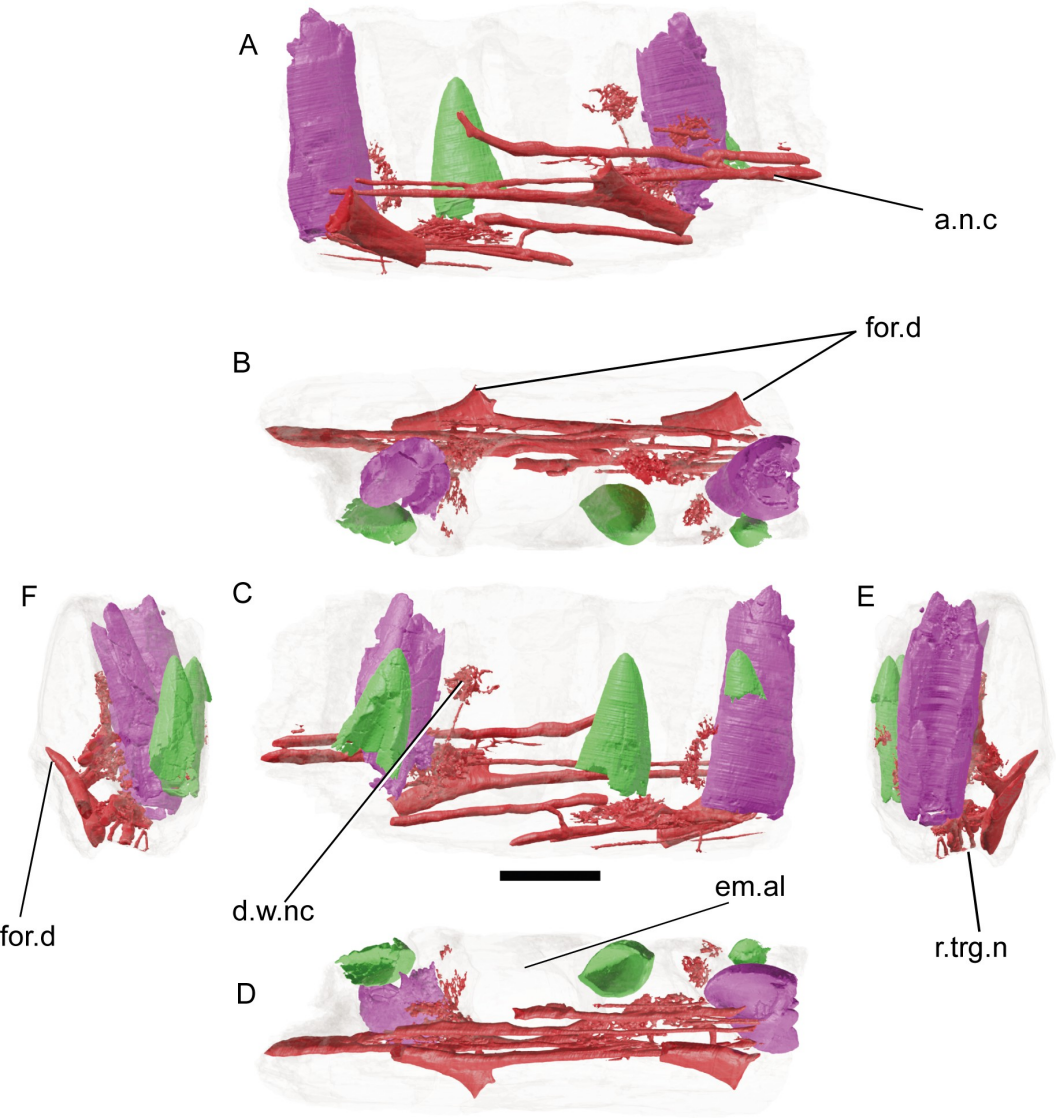
CT-scan of a fragment of dentary of *Iberospinus* natarioi (ML1190-2), showing the neurovascular system and the replacement teeth. **A**, lateral, **B**, occlusal, **C**, medial, **D**, ventral, **E**, anterior, and **F** posterior views. **a.n.c**, anteroposterior neurovascular canals, **d.w.nc**, dense web of neurovascular canals, em.al, empty alveolus, **for.d**, medial foramina of the dentary, **r.trg.n**, roof of the mandibular branch of the trigeminal nerve. Scale bar is 10 mm. Voxel size of the model: 0.047616.

Interestingly, the replacement teeth appear to follow a different replacement pattern than in ML1190-1, the anterior tip of the dentary. Both the first and the last alveoli have two in-situ replacement teeth. As expected the second alveolus has only one replacement tooth, but the third is barren of any replacement tooth and in the fourth there are two replacement teeth, when only one would be expected, as the first alveoli has two of them (Fig 8). This alteration of the pattern might have been caused by the traumatic removal of one of the teeth, and has been reported in Archosauria and lizards [76, 77]. Nevertheless, it is not possible to exclude the fact that the pattern of tooth replacement varies posteriorly through the dentary.

For the neurovascular anatomy, a series of eight anteroposteriorly oriented canals run through most of the fragment, labially from the preserved teeth. The six ventrally situated progress dorsally from their origins in what is interpreted as the roof of the trigeminal nerve canal. Only one of them preserves a clear foramen that opens to the exterior, this opens up laterally from the fragment and has a particular mediolaterally compressed section. There are also several extremely fine, branched canals around the alveoli that most likely served to innervate the teeth.

#### Distal premaxillary or mesial maxillary teeth (ML1190-3)

ML 1190–3 was already described in detail by Mateus et al. [42]. The measurements of ML 1190–3 were taken according to [29] plus the total length including root and the root height measured distally, the measurements can be found in the S4 File.

**Fig 9:**
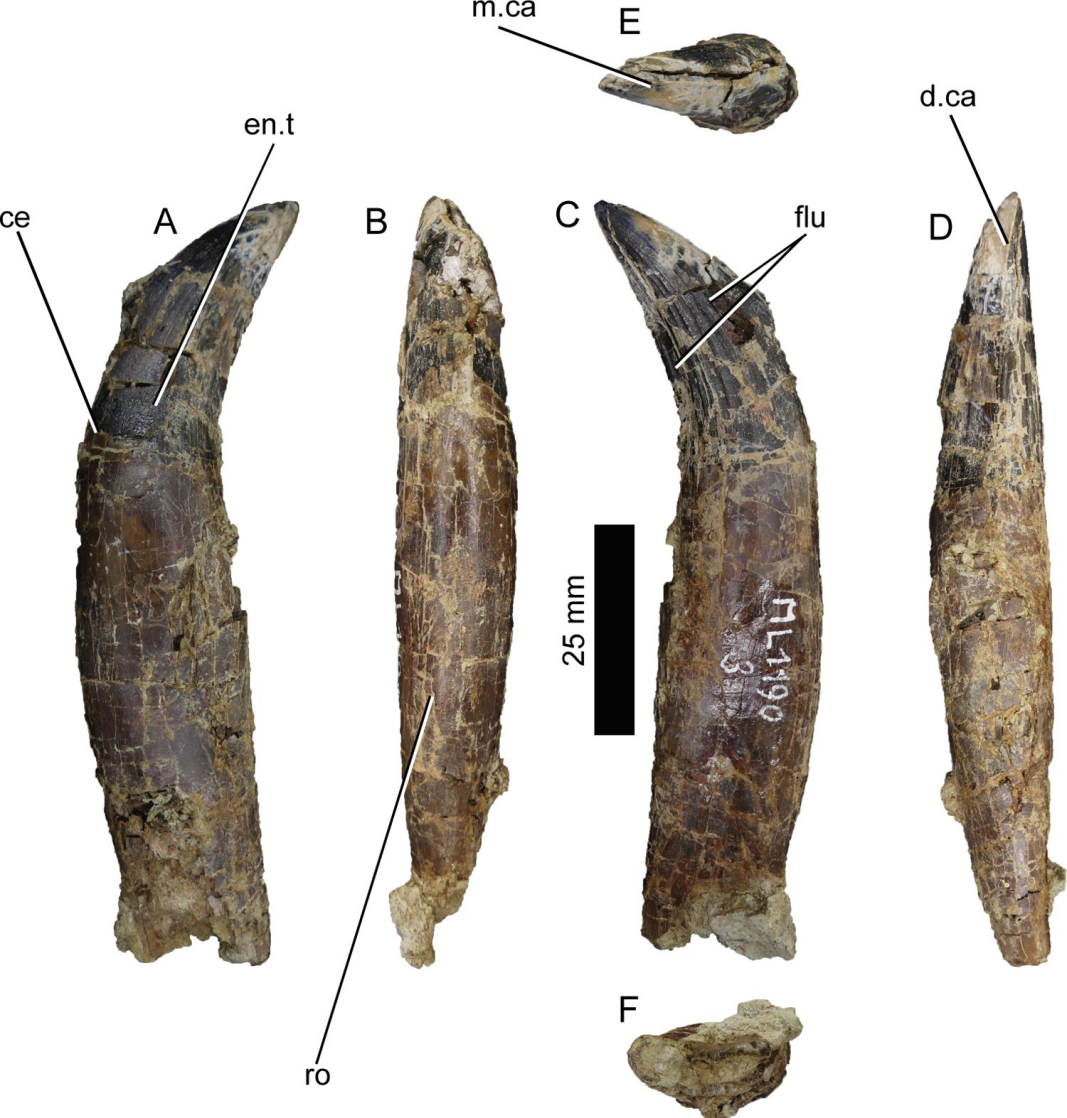
Premaxillary or anterior maxillary tooth of *Iberospinus natarioi* (ML1190-3). **A**, lingual, **B**, mesial, **C**, lingual, **D**, distal, **E**, occlusal, and **F** ventral view. **ce**, cervix, **d.ca**, distal carina, **en.t**, enamel texture, **flu,** flutes, **m.ca**, mesial carina, **ro**, root.

ML1190-3 (Fig 9) presents a relatively conical shape (conidonty), a crown base ratio bigger than 0.64 (0.81), a crown height between 3–6 cm (~3.7 cm), a subcircular cross section at the cervix, minute and numerous denticles (more than 250 denticles per carina), mesial denticles significantly smaller than the distal ones, flutes, marginal undulations of the crown, and veined enamel texture. All of these characters are present in Baryonychinae according to Hendrickx et al., 2019 [29]. It is also worth noting that the tooth does not present transverse undulations and its mesial denticles disappear just before reaching the cervix. This condition is similar to that observed in the baryonychine teeth described by Canudo et al., 2008 [78] from the Iberian Peninsula, where the mesial carina disappears about 2/3 or half way through the crown. The denticles vary relatively randomly in size across the height of the crown. Given the position of the most marked flutes (described by Hendrickx et al., 2019 [29] as usually present in the lingual side in *Baryonyx*) and the overall morphology it can be concluded that this tooth is a right premaxillary or mesial maxillary tooth.

#### Fragmentary tooth (ML1190-4)

ML1190-4 is a fragmentary tooth crown (41.12 mm long), more convex distally than ML1190-3. It bears nine flutes in both lateral faces of the crown. Both mesial and distal carinae bear denticles all over their preserved lengths. The surface texture is slightly veined. Measurements can be found in the S4 File.

#### Posterior dorsal neural arch (ML 1190–7)

**Fig 10:**
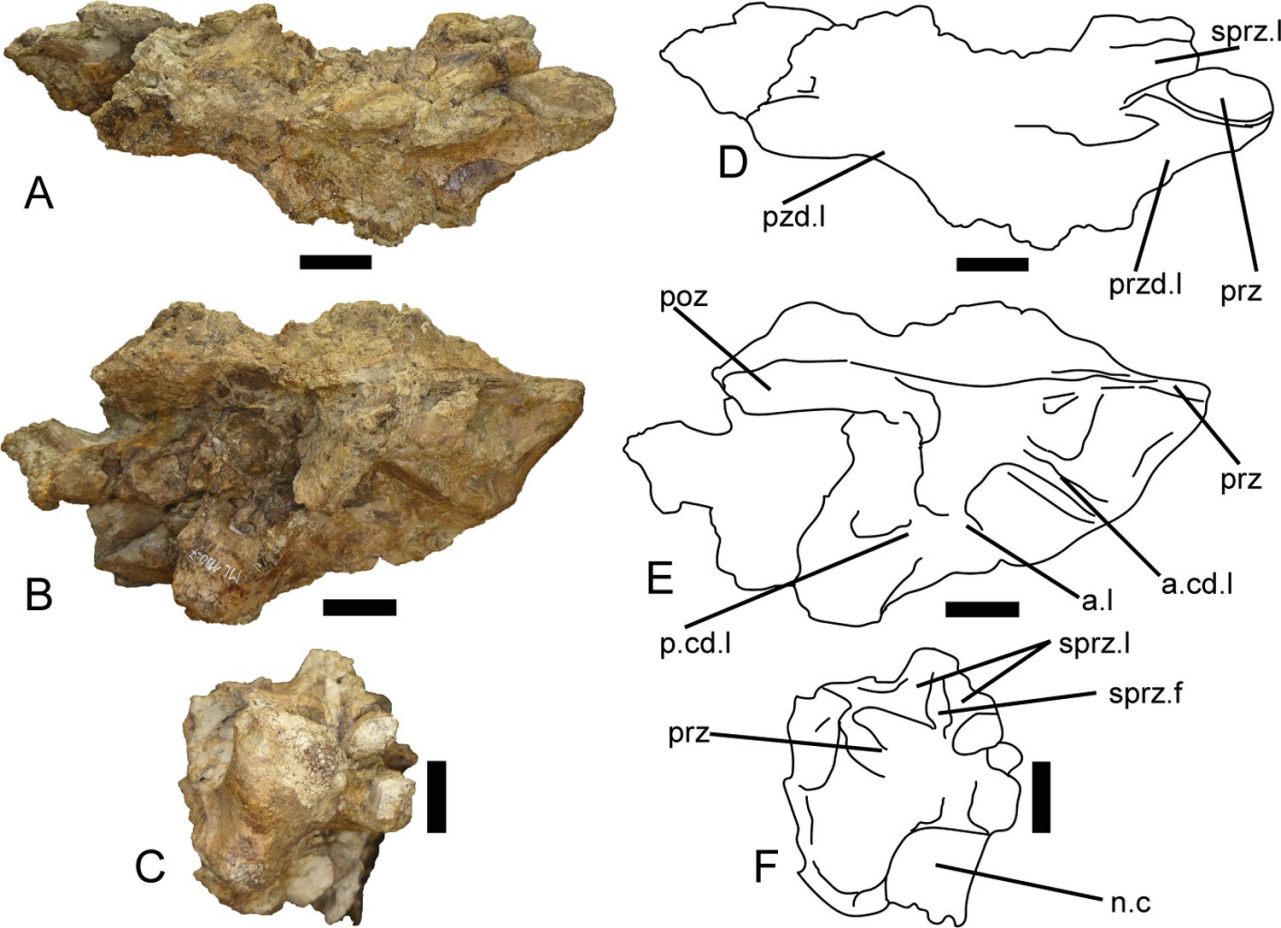
Partial dorsal neural arch of *Iberospinus natarioi* (ML1190-7). **A, D**; dorsal, **B, E**; lateral, and **C, F**; anterior view. Scale bar is 25 millimeters. **a.cd.l**, anterior centrodiapophyseal laminae, **a.l**, accessory lamina, **n.c**, neural canal, **poz**, postzygapophysis, **prz**, prezygapophysis, **przd.l**, prezygadiapophyseal lamina, **pzd.l**, postzygapodiapophyseal lamina, **p.cd.l**, posterior centrodiapophyseal laminae, **sprz.f**, spinoprezygapophyseal fossa, **sprz.l**, spinoprezygapophyseal laminae.

This piece is a right side of a posterior dorsal neural arch, given the relative position of the zygapophyses. It measures 160 mm in its anteroposterior axis. The neural spine and the right diapophysis are missing, and the majority of the left side is crushed and broken beyond any possible identification of its features (Fig 10).

The right prezygapophysis is eroded in its anteriormost part, but is still possible to distinguish the spinoprezygapophyseal laminae, which includes a deep and elongated spinoprezygapophyseal fossa. There are also some remains of the left prezygapophysis that hang below the left spinoprezygapophyseal laminae. Therefore, it is still possible to distinguish the roof of the neural canal between the two prezygapophyses, which is mostly smooth, lacking the ridges that can be observed in the floor of the neural canal of other vertebrae in the specimen. It is also possible to see some of the broken parapophysis and a deeply posteriorly excavated infraprezygapophyseal fossa. Above this fossa is a prezygadiapophyseal lamina that runs in a straight line from the prezygapophysis to the anterior end of the diapophysis. Although badly damaged it is possible to see the anterior centrodiapophyseal lamina (or parapodiapophyseal lamina, as it finishes in the posterodorsal side of the parapophysis) whose steep posteroventral side delimits the centrodiapophyseal fossa. Running posteroventrally from the diapophysis there is the posterior centrodiapophyseal lamina, which is reinforced in its first half by the accessory lamina that rises from the centrodiapophyseal fossa. On the other side of the centrodiapophyseal lamina, the centropostzygapophyseal fossa is deeply excavated in its posteroventral part. Dorsally delimiting this fossa there is the postzygapodiapophyseal lamina, which is slightly concave in dorsal view. The prezygapophysis is slightly anteroposterly elongated.

#### Posterior dorsal neural arch (ML1190-6/8)

**Fig 11:**
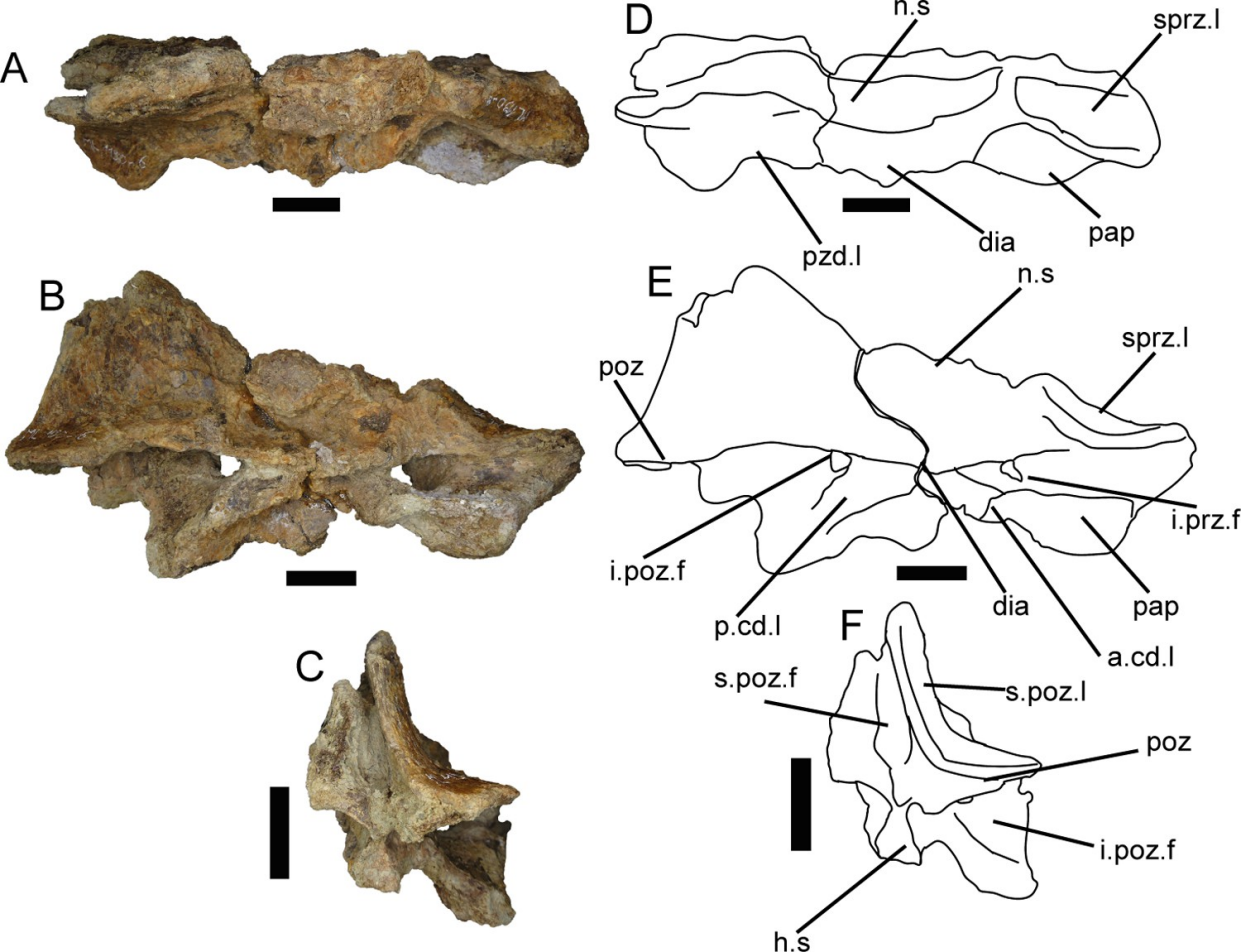
Partial dorsal neural arch of *Iberospinus natarioi* (ML1190-6/8). **A, D**; dorsal, **B, E**; lateral, and **C, F**; posterior view. Scale is 25 mm. **a.cd.l,** anterior centrodiapophyseal laminae, **h.s**, hyposphene, **i.poz.f**, infrapostzygapophyseal fossa, **i.prz.f**, infraprezygapophyseal fossa, **n.s**, base of the neural spine, **p.cd.l**, posterior centrodiapophyseal laminae, **pap**, parapophysis, **poz**, postzygapophysis, **pzd.l**, postzygapodiapophyseal lamina, **s.poz.f**, spinopozygapophiseal fossa, **s.poz.l**, spinopozygapophiseal lamina, **sprz.l**, spinoprezygapophyseal laminae.

This piece comprises the right side of a posterior dorsal neural arch, given the relative position of the zygapophyses, which measures 191 mm along its anteroposterior axis. ML1190-6/8 is a posterior arch that was already described by Mateus et al., 2011 [42], that still possesses part of the neural spine, the hyposphene, the right postzygapophysis, and parts of the left postzygapophysis. ML1190-8 is the anterior part of the neural arch and comprises part of the right prezygapophysis and the remnants of the parapophysis (Fig 11).

The right prezygapophysis is mostly eroded along its anterior part and in the posterior, it is not possible to distinguish the spinoprezygapophyseal lamina. Ventrolaterally from the prezygapophysis, the infraprezygapophyseal fossa is deeply excavated, even when the prezygadiapophyseal lamina is missing, likely taphonomic. The parapophysis is mostly missing with only part of the centrodiapophyseal lamina remaining, oriented anteroposteriorly along the major axis of the neural arch. The breakage between ML1190-6 and ML1190-08 is situated more or less in the middle of the diapophysis, where only the base remains. It is still possible to distinguish the centrodiapophyseal fossa, delimited on its posterior part by a thick posterior centrodiapophyseal lamina. The centropostzygapophyseal fossa is deeply marked and along the dorsal part it is bordered dorsally by a partially broken postzygapodiapophyseal lamina. The neural spine is at least 32 mm wide at its base and presents an extremely deep (15 mm) spinopostzygapophyseal fossa on its posterior area. The most distal part of the postzygapophysis is slightly eroded. The hyposphene is subtriangular in posterior view and is 16 mm wide.

#### Dorsal vertebral centrum (ML1190-234)

**Fig 12:**
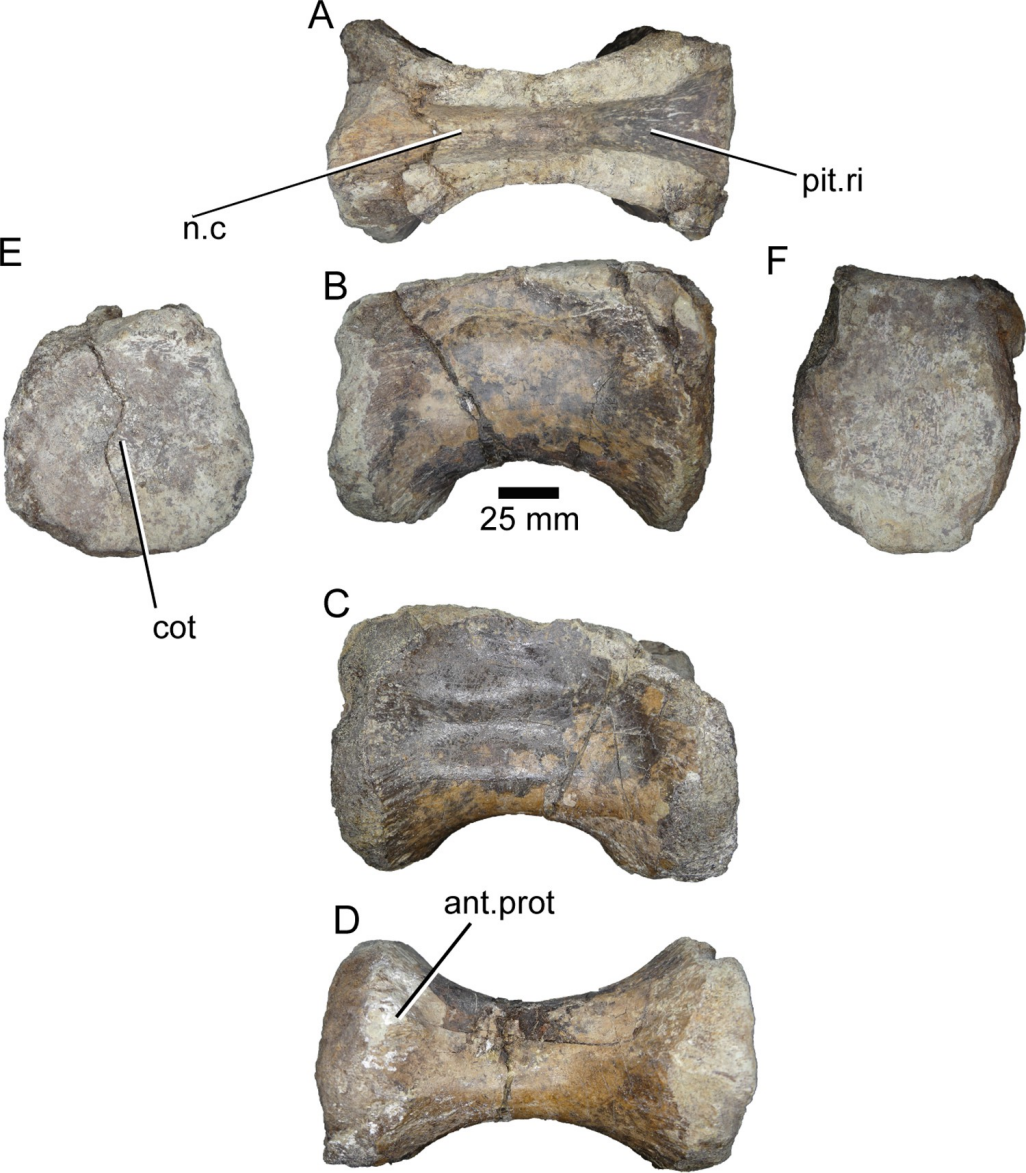
Dorsal vertebra of *Iberospinus natarioi* (ML1190-234). **A**, dorsal, **B, C**, lateral, **D**, ventral, **E**, anterior, and **F** posterior view. **ant.prot**, anteroventral protuberance, **cot**, cotyle, **n.c**, neural canal, **pit.ri**, pits and ridges in the floor of neural canal.

This dorsal vertebral centrum (~160 mm long) was discovered during the excavation of June 2020. Given the straight facets, the relatively robust middle section and other characters it is interpreted as a posterior dorsal centrum. The neural arch is completely missing, but the walls along the side of the neural canal that belonged to the centrum are still in place. Given the texture of the dorsal part of these walls of the neural canal, it is likely that the neural arch was not totally fused and dislodged along the neurocentral suture. The anterior facet is damaged, especially around the left anterodorsal portion, although the rims of both facets of the centrum are slightly worn. There is a fracture running anterodorsally-posteroventrally in the anterior half of the vertebra (Fig 12).

The whole centrum is longer than tall, a condition similar to that seen in *Suchomimus* or *Baryonyx* mid-posterior dorsal centra. This strongly contrasts with the appearance of the 12th dorsal vertebra of *Ichthyovenator*, much taller, and much more constricted dorsoventrally in the middle section. Of the three recovered mid-posterior centra of *Vallibonavenatrix*; ML1190-234 only resembles the posteriormost of them, with the other being more constricted in the middle section in ventral view [6, 9, 10, 14].

The anterior facet is mostly flat, with a certain concavity along the upper half and a small cotyle located in the middle, approximately 7 mm in diameter.

Along the dorsal part of the centrum, the floor of the neural canal presents several ridges and pits. The neural canal is shallow and thins posteriorly for about 53 mm, where it reaches the minimum diameter at 10 mm and is most shallow, then it immediately deepens and widens posteriorly for another 70 mm.

Both lateral sides of the centrum show no traces of pleurocoels or foramina, with some small ridges along the posterior part, a condition also present in the mid-posterior centra of *Vallibonavenatrix*. The ventral side of the centrum shows a smooth surface; mostly flat and without keels or ridges. In the anterior part of the ventral surface, there is a small anteroposteriorly elongated protuberance, slightly off centered to the right in posterior view, that measures 12 mm mediolaterally and 17 mm anteroposteriorly.

The posterior facet is shallow, concave and more dorsoventrally compressed than the anterior part.

#### Dorsal ribs (ML1190-11, ML1190-12, ML1190-28, ML1190-239)

**Fig 13:**
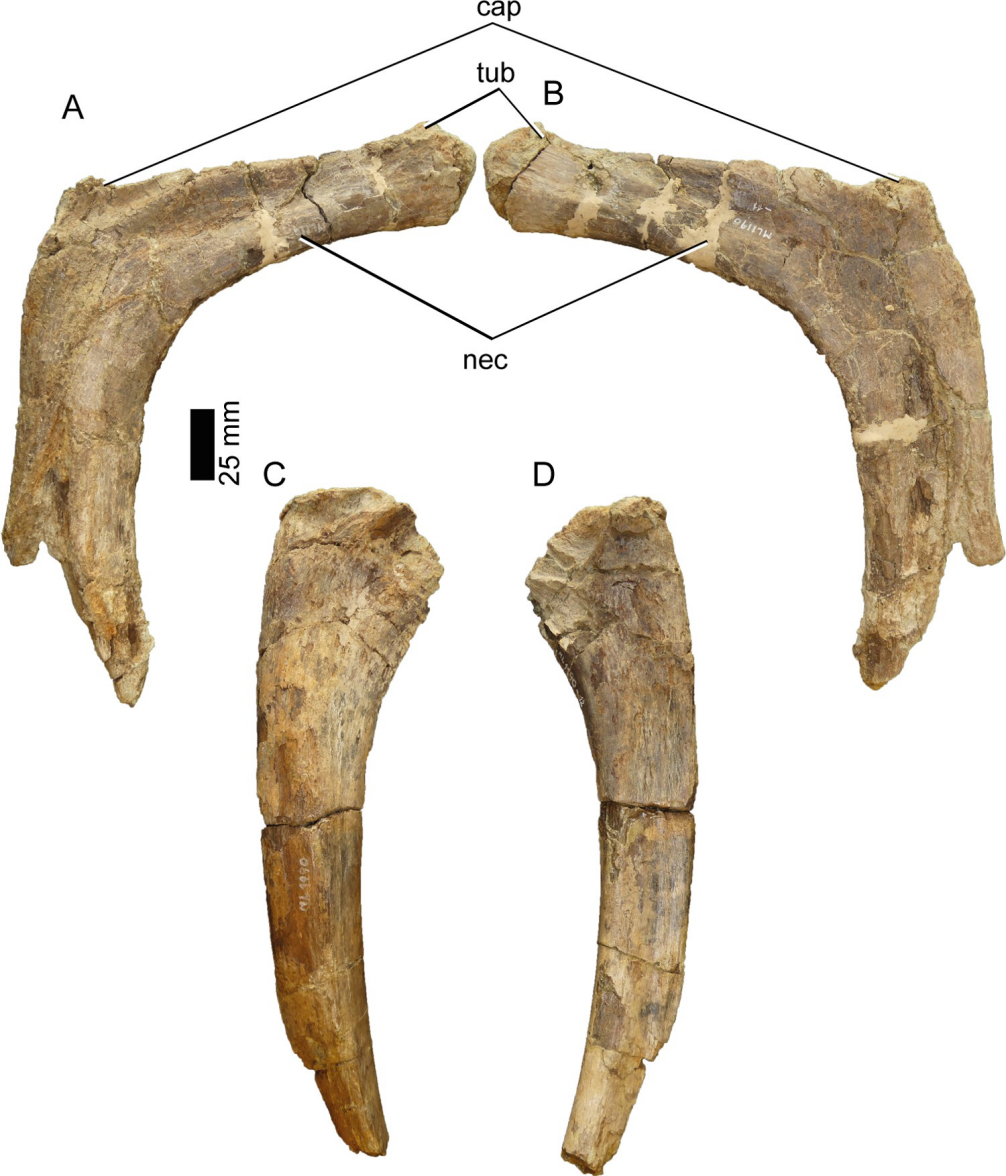
Ribs of *Iberospinus natarioi* (ML1190-11, upper; ML1190-12; lower). **A**, anterior, **B**, posterior, **C**, anterior, and **D** posterior view. **cap**, capitulum, **nec**, neck of the rib, **tub**, tuberculum.

Dozens of rib fragments have been found at the site, however only four are complete enough to be described. ML1190-11 and ML1190-12 were already described by Mateus et al., 2011 [42], who mentioned that the tuberculum was less pronounced than in the holotype of *Baryonyx*. These ribs are similar to the mid-dorsals of the holotype, lacking the tall capitulum of the anterior dorsals, but neither possessing the L-shaped cross-section with the strong posterior projection seen in the posterior dorsal ribs (Fig 13).

**Fig 14:**
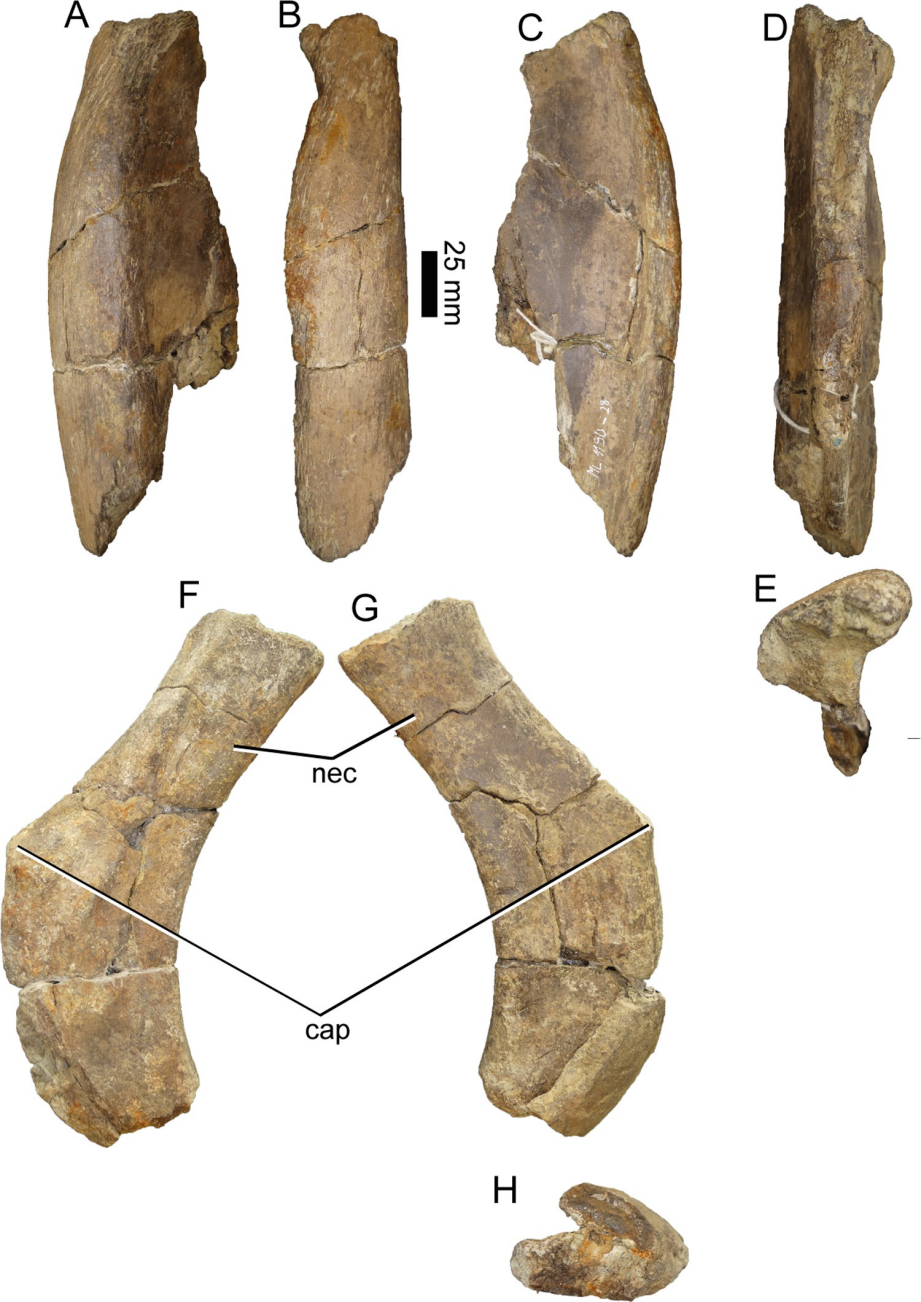
Ribs of *Iberospinus natarioi* (ML1190-28, upper; ML1190-239; lower). **A**, anterior, **B**, lateral, **C**, posterior, **D**, medial, **E**, distal, **F**, anterior, **G**, posterior and **H** distal view. **cap**, capitulum, **nec**, neck of the rib.

ML1190-28 and ML1190-239 seem to be relatively similar to the posteriormost ribs of the holotype of *Baryonyx*, with ML1190-239 possessing a strongly marked posterior projection that bends medially into a groove, giving this rib cross-section a V-like appearance. ML1190-28 is the widest rib (mediolaterally) discovered, more than 60 mm along this axis, despite being partially incomplete. Nevertheless, as noted by Mateus et al., 2011 [42] this widened surface becomes rapidly more oval in cross section as the rib tapers ventrally, giving them a more usual rib shape (Fig 14).

#### Caudal vertebrae

15 caudal vertebrae varying in states of preservation were found at the site of Praia de Aguncheiras. Their extended description and images can be found in S5 File and their measurements can be found in S6 File. Herein, we will discuss the main features of the better preserved vertebrae.

**Fig 15:**
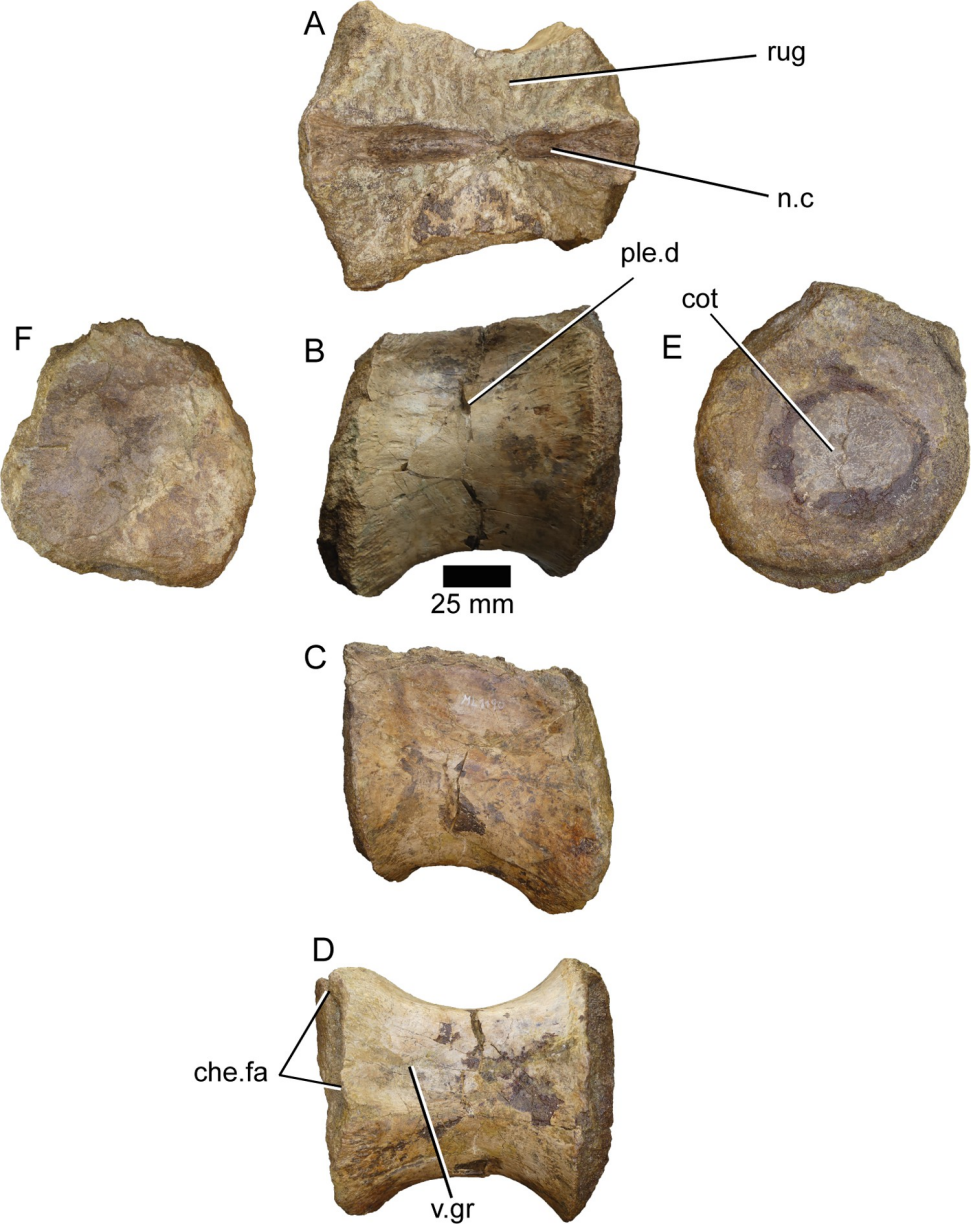
Caudal vertebra of *Iberospinus natarioi* (ML1190-15). **A**, dorsal, **B, C;** lateral, **D**, ventral, **E**, anterior, and **F,** posterior view. **che.fa**, chevron facets, **cot**, cotyle, **n.c**, neural canal, **ple.d**, pleurocelic depression, **rug**, rugosities, **v.gr**, ventral groove.

**ML1190-15** (Fig 15): This vertebra is interpreted as the anteriormost preserved and is the biggest of the caudal vertebrae of ML1190, which was already described by Mateus et al., 2011 [42]. It comprises a well preserved centrum lacking the neural arch. The vertebra is roughly as tall as long. The anterior facet is concave and slightly oval, with an anterior cotyle of about 7 mm of diameter in the middle. There is a shallow pleurocentral depression along the dorsal half of the centrum. Ventrally the vertebra presents a clearly marked groove along the midline whose edges end posteriorly in the chevron facets. Overall, the centrum is more similar in outline to that of the anterior caudals of *Vallibonavenatrix* or *Ichthyovenator* (oval-shaped, with the dorsal surface slightly reduced) than the anterior caudals of the *Spinosaurus* neotype (square-shaped).

**Fig 16:**
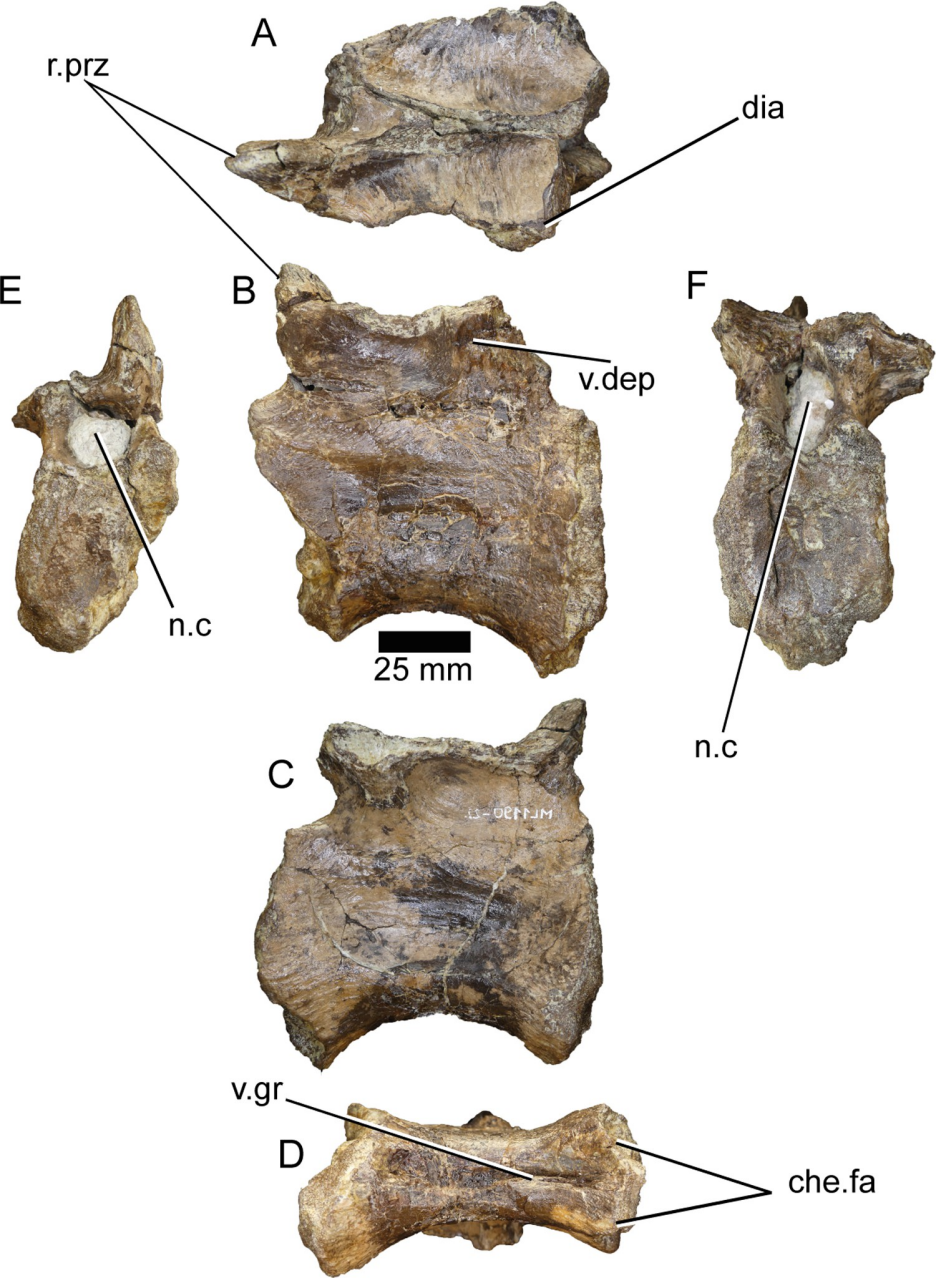
Caudal vertebra of *Iberospinus natarioi* (ML1190-22). **A**, Dorsal, **B, C**, lateral, **D**, ventral, **E**, anterior and **F** posterior view. **che.fa**, chevron facets, **dia**, diaphysis, **n.c**, neural canal, **prz**, remains of prezygapophysis, **v.dep**, ventral depression in the neural arch, **v.gr**, ventral groove.

**ML1190-22** (Fig 16) was previously an unpublished vertebra. It is strongly crushed mediolaterally, with the right ventral part of the centrum damaged. It still preserves the neural arch, although with an eroded and damaged right prezygapophysis. The neurocentral suture is almost invisible along the lateral surface of the centrum, with only the anteriormost portion visible. The presence of minor webbing is observed in the dorsal part of the diapophysis. The ventral groove runs across the midline of the vertebra, clearly marked, even in the anterior half. In the neural arch, the right prezygapophysis elevates to an angle of about 70° from the dorsal margin of the centrum. The spinoprezygapophyseal laminae are not well preserved but the spinoprezygapophyseal fossa delimited by them is much shallower and less elongated anteroposteriorly than in ML1190-16. Along the anteroventral part of the base of the transverse processes there is a shallow depression. In lateral view the centrum is relatively similar to some caudals of *Riparovenator*, although ML1190-22 seems less elongated and more robust.

**Fig 17:**
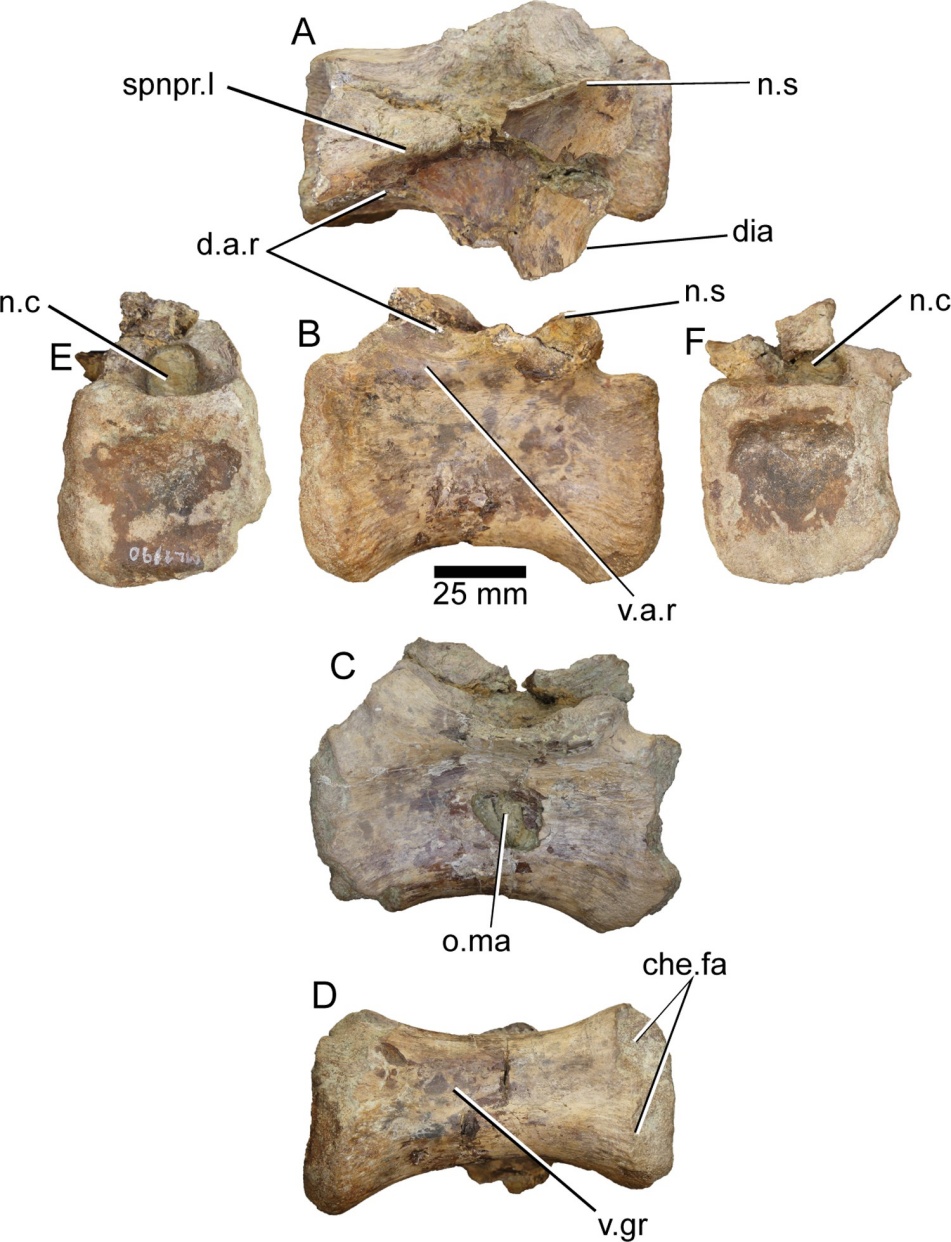
Caudal vertebra of *Iberospinus natarioi* (ML1190-18). **A**, dorsal, **B, C;** lateral, **D**, ventral, **E**, anterior, and **F,** posterior view. **che.fa**, chevron facets, **d.a.r**, dorsal anterior ridge, **dia**, diaphysis, **n.s**, broken neural spine, **n.c**, neural canal, **o.ma**, oval mark, **spnpr.l**, spinoprezygapophyseal lamina, **v.a.r**, ventral anterior ridge, **v.gr**, ventral groove.

**ML1190-18** (Fig 17) was interpreted as a mid-posterior caudal vertebra by Mateus et al., 2011 [42]. Along its left side, the centrum bears an oval mark with the major axis pointing anterodorsally-posteroventrally; measuring 21 mm in length and 11mm in width. This mark was already noted by Mateus et al., 2011 [42] and attributed to perimortem damage due to predation or scavenging. Interestingly, similar marks appear in unpublished *Suchomimus* caudals and some of the caudals of the *Spinosaurus* neotype. This might point towards a tendency in spinosaurid caudals to suffer this particular type of damage due to taphonomy. The neural arch of the vertebra is still in place and the bases of both transverse processes and the neural spine are already in place, in spite of this last feature being slightly deformed. The pre- and post- zygapophyses are completely absent. There are two ridges running anteriorly from the anterior part of the base of the transverse processes. The ventral one finishes in the lateral wall of the neural canal opening, the dorsal one runs towards the spinoprezygapophyseal lamina, with both ridges delimiting a fossa anterior to the transverse processes.

**Fig 18:**
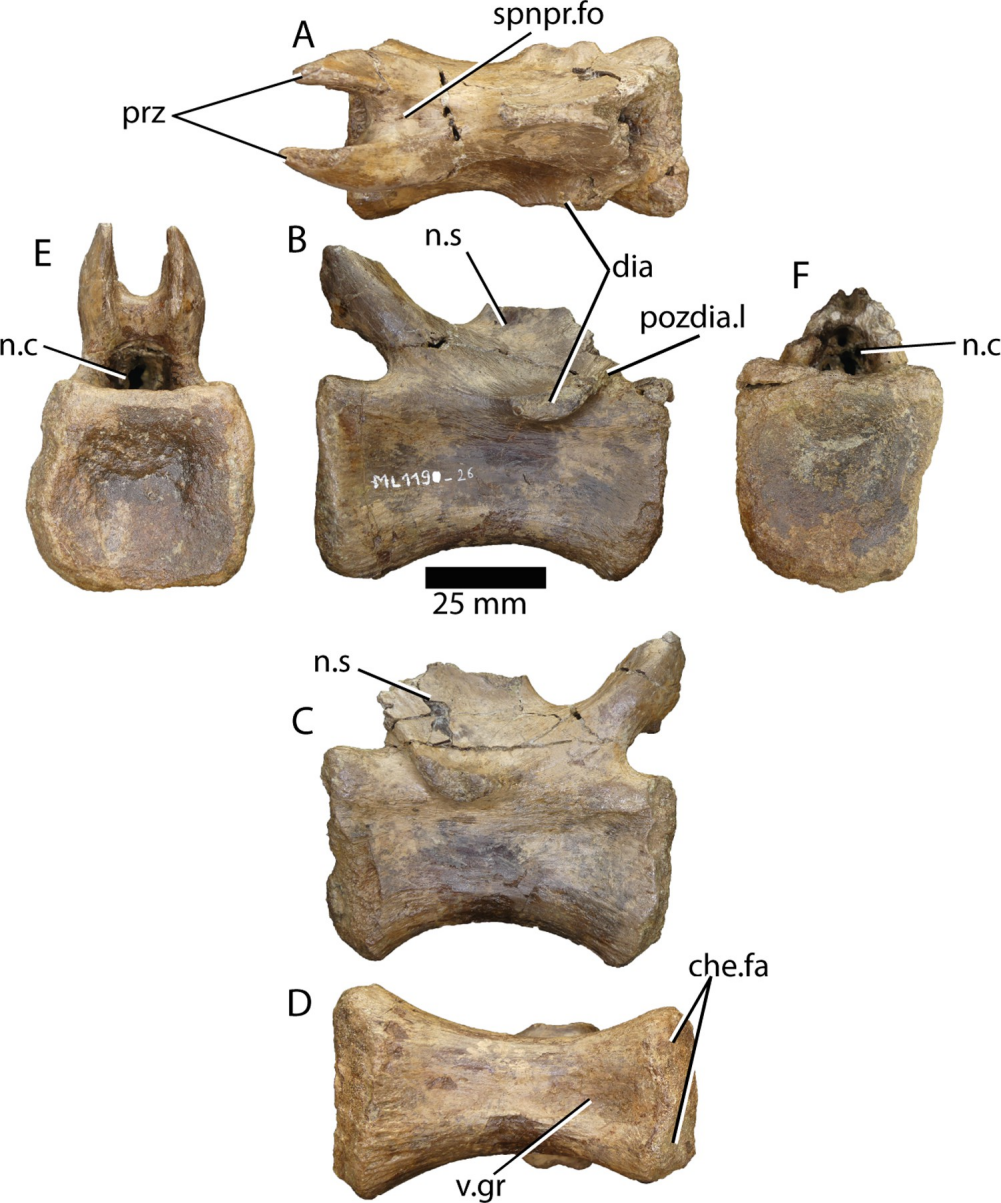
Caudal vertebra of *Iberospinus natarioi* (ML1190-26). **A**, Dorsal, **B, C**, lateral, **D**, ventral, **E**, anterior and **F** posterior view. **che.fa**, chevron facets, **dia**, diaphysis, **n.c**, neural canal, **n.s**, broken neural spine, **pozdia.l**, postzygapodiapophyseal laminae, **prz**, prezygapophyses, **spnpr.fo**, spinoprezygapophyseal fossa, **v.gr**, ventral groove.

**ML1190-26 (Fig 18)** was also a previously unpublished vertebra despite being one of the best preserved of the specimen ML1190. The neural arch is still in place, but is better preserved in the anterior part, with both prezygapophyses still in place as well as the base of the neural spine and the bases of both transverse processes. The prezygapophyses protrude just slightly anteriorly over the anterior facet of the centrum in dorsal view (this contrasts with comparable vertebrae of *Spinosaurus*, whose prezygapophyses never overhang the centrum). The prezygapophyses project anterodorsally in an angle of ~70° with the centrum and are about 30 mm long from the base (20 mm from the anterior end of the spinoprezygapophyseal fossa), with a dorsoventral diameter of about 18 mm. Their facets are pointing medially with a distance of 11mm between them. The spinoprezygapophyseal laminae are not well-developed and converge in the anteriormost point of the base of the neural spine, delimiting an extremely shallow spinoprezygapophyseal fossa. The anterior end of the neural spine is 8 mm in width and is situated only slightly anteriorly of the anteroposterior middle point of the vertebra. The diapophyses bases are situated, posteriorly to the aforementioned point and they possess a ridge running posterodorsally towards the point where the bases of the postzygapophyses would be (postzygapophyseal laminae) in an angle with the centrum similar to that formed by the prezygapophyses.

**Fig 19:**
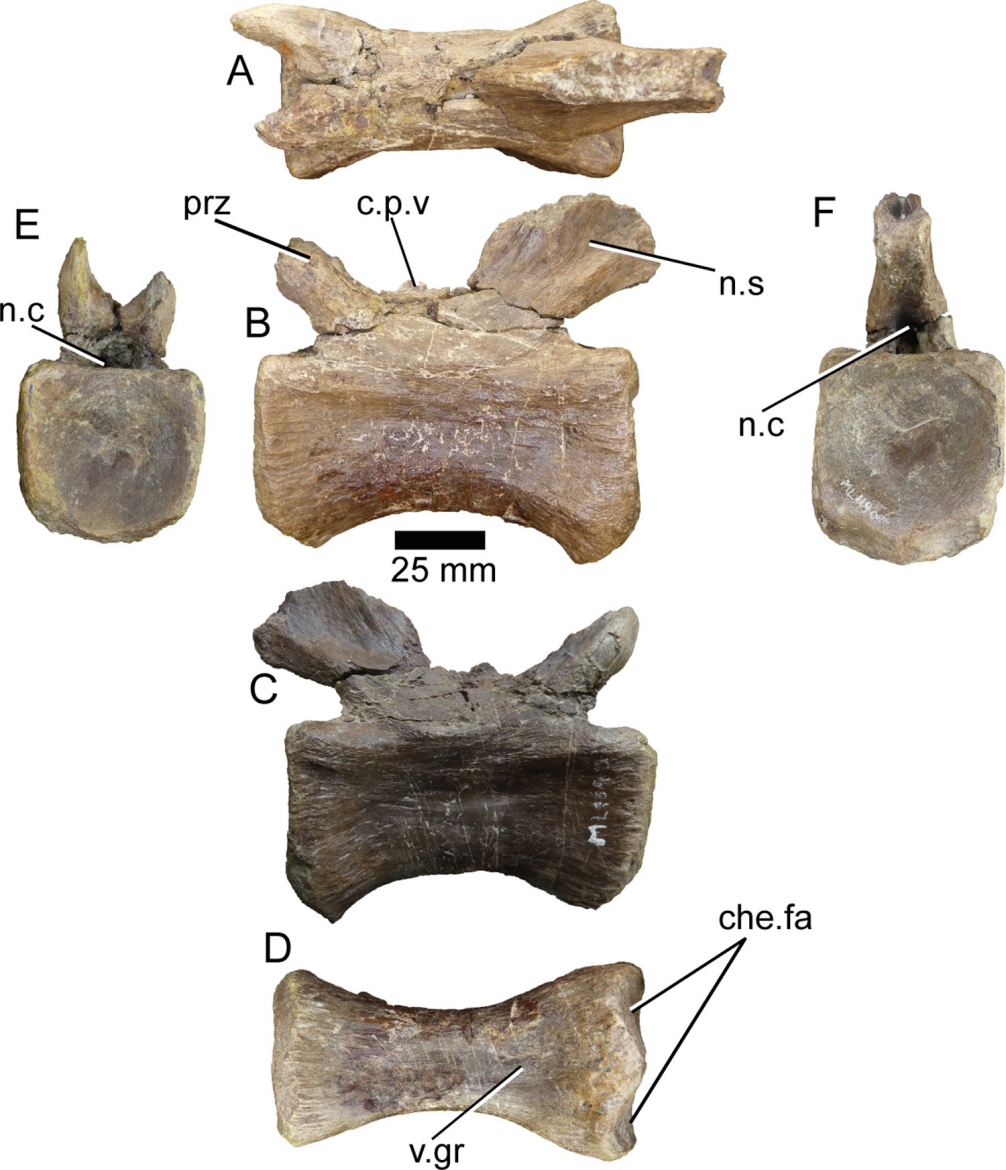
Caudal vertebra of *Iberospinus natarioi* (ML1190-19). **A**, dorsal, **B, C;** lateral, **D**, ventral, **E**, anterior, and **F,** posterior view. **c.p.v**, cranial process of the vertebra, **che.fa**, chevron facets, **n.c**, neural canal, **n.s**, neural spine, **prz**, prezygapophysis, **v.gr**, ventral groove.

**ML1190-19** (Fig 19) is a vertebra already mentioned in [42]. It is the best preserved-vertebra of the entire series. The centrum is completely preserved, with almost no erosion along the rims of the facets and the original surface mostly intact. The neural arch is more damaged, with cracks over most of its surface. The ventral surface presents a shallow groove anteroposteriorly, with the ridges delimiting it being relatively smooth. The prezygapophyses form an angle of ~60° with the centrum, with the anterior facets of them slightly overhanging in dorsal view. They are separated by approximately15 mm, although the distance is slightly distorted by the reconstruction. They are also 15 mm tall dorsoventrally and project another 15 mm anterodorsally from the neural arch. It is not possible to observe if there are spinoprezygapophyseal laminae or fossa because of the preservation, however there is a small protuberance situated in the anterior portion of the vertebra, 6 mm tall and 5 mm wide. This is herein interpreted as the cranial process of the vertebra, also mentioned from a spinosaurid from Thailand, the Phuwiang spinosaurid B [8]. There are no transverse processes in this vertebra, but there are two smooth ridges only 2 or 3 mm wide, slightly anterior to the anteroposterior midpoint of the vertebra that could be homologous to the transverse processes preserved in more anterior vertebrae. Therefore, the smooth ridge that runs from their posterior part towards the postzygapophyses would be homologous to the diapostzygapophyseal lamina. The neural spine is about 14 mm wide in the base and is broken ~40 mm above the centrum. There is a triangular canal exposed by this breakage running posteroventrally across it, 9 mm tall and 6 mm wide along the ventral surface. Only the anteriormost part of the postzygapophyses is preserved. They overhang the posterior surface of the centrum in dorsal view and are separated ventrally for about 5 mm at their base. As previously mentioned, this vertebra closely resembles that of the Phuwiang spinosaurid B (SM-PW9B-12), although there are three main differences with this one: 1) The centrum of ML1190-19 is more robustly built, being less elongated, 2) the prezygapophyses and the neural spine present a less steep angle related with the centrum, and 3) the prezygapophyses reach just slightly beyond the anterior facet of the centrum, unlike in the Thai spinosaurid.

**Fig 20:**
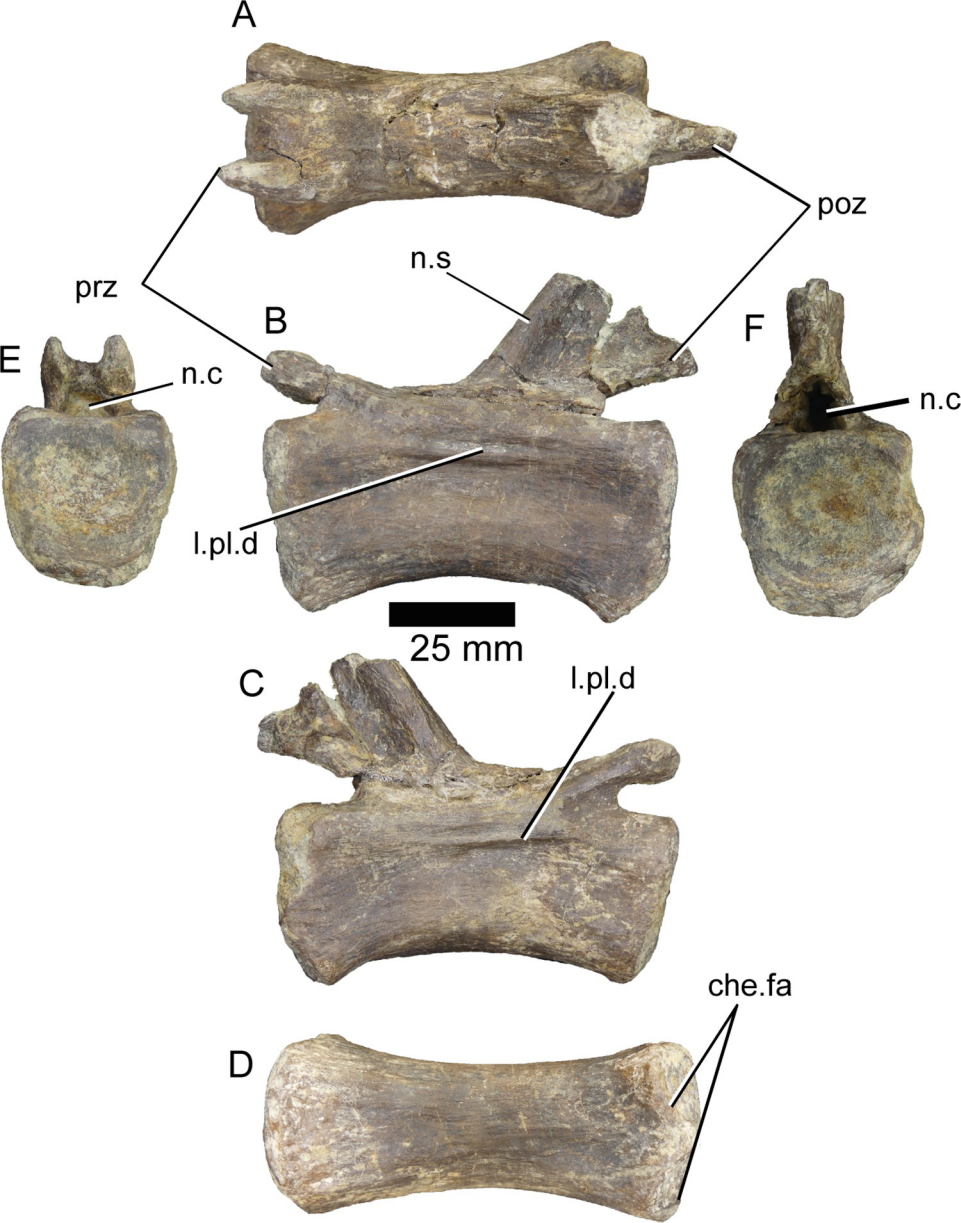
Caudal vertebra of *Iberospinus natarioi* (ML1190-240). **A**, dorsal, **B, C;** lateral, **D**, ventral, **E**, anterior, and **F,** posterior view. **che.fa**, chevron facets, **l.pl.d**, lamina in pleurocelic depression, **n.c**, neural canal, **n.s**, neural spine, **poz**, postzygapophysis, **prz**, prezygapophysis.

**ML1190-240** (Fig 20) is a caudal vertebra recovered during the excavation of June 2020 and is one of the best-preserved elements from that field season. The centrum is complete and mostly intact, as well as the prezygapophyses and the neural arch. The lateral surfaces of the centrum display relatively deep and anteroposteriorly elongated concavities, about 30 mm anteroposteriorly in length and 11 mm wide dorsoventrally. They present a ridge along their dorsal half that extends anteroposteriorly across their length and is about 3 mm dorsoventrally thick; these laminae do not appear in the caudal vertebrae of any other spinosaurid to our knowledge. The ventral surface of the centrum is mostly flat with a small ridge, centered in its posterior half. The prezygapophyses have their articular surfaces almost perfectly 90° angle with respect to the centrum in anterior view. They are separated by 9 mm and are 8 mm dorsoventrally wide and project 6 mm anteriorly from the neural arch in a sharp angle <30° in lateral view, slightly overhanging the centrum. Posterior to them in the neural arch there are no signs of an anterior process of the neural spine or spinoprezygapophyseal fossa, just a mostly flat surface. The base of the neural spine is 13 mm wide mediolaterally. It presents two ridges running dorsally along its length from each side of its anterior margin, separated by 6 mm and 8 mm wide anteroposteriorly. In the *Spinosaurus* neotype, these two ridges are extremely well developed, comprising almost the entirety of the anterior margin of the neural spine. A thin lamina, 4 mm thick connects the posterior margin of the spine with the fused postzygapophyses, whose articular surfaces are perpendicular to the centrum in posterior view. They project 22 mm posteriorly to the end of the neural canal and overhang by several mm the posterior facet of the centrum.

**Fig 21:**
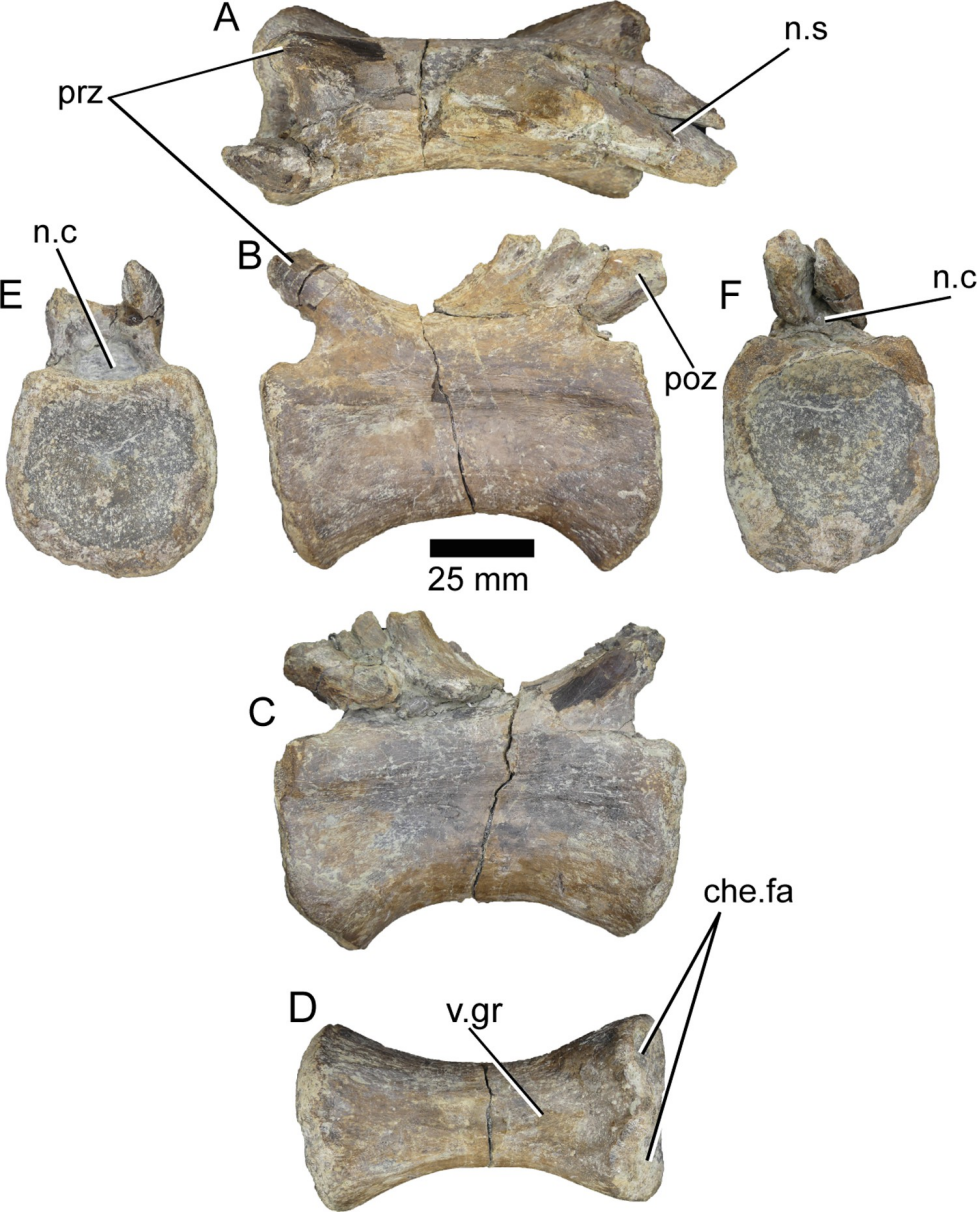
Caudal vertebra of *Iberospinus natarioi* (ML1190-241). **A**, dorsal, **B, C;** lateral, **D**, ventral, **E**, anterior, and **F,** posterior view. **che.fa**, chevron facets, **n.c**, neural canal, **n.s**, deformed base of the neural spine, **poz**, postzygapophysis, **prz**, prezygapophysis, **v.gr**, ventral groove.

**ML1190-241** (Fig 21) is also a caudal vertebra recovered during the excavation of June 2020. The right prezygapophysis has eroded away along with most of the right side of the neural arch is mostly gone, being attached thanks to sediment. The left side (in posterior view) is much better preserved with just minor cracks along its surface. The postzygapophyses are highly damaged, but still recognizable. The lateral surfaces of the centrum do not show any pleurocels or concavities. Again, the centrum is more robust than those of comparable vertebrae (e.g., *Riparovenator)*. The ventral surface has a shallow groove whose ridges finish at the chevron facets, it is about 11 mm wide. The prezygapophyses are separated by about 10 mm, do not overhang the centrum, and project in a 30° angle from the neural arch in lateral view with the centrum. The articular facet of the preserved right prezygapophysis is 14 mm wide dorsoventrally and projects about 12 mm in front of the neural arch. There is an extremely thin lamina running medially across the dorsal surface of the neural arch from the base of the neural spine to the point of connection of the prezygapophyses. The neural spine is about 13 mm wide at its anterior portion, where it emerges from the neural arch. The postzygapophyses slightly overhang the posterior centrum facet.

#### Scapula (ML1190-10)

**Fig 22:**
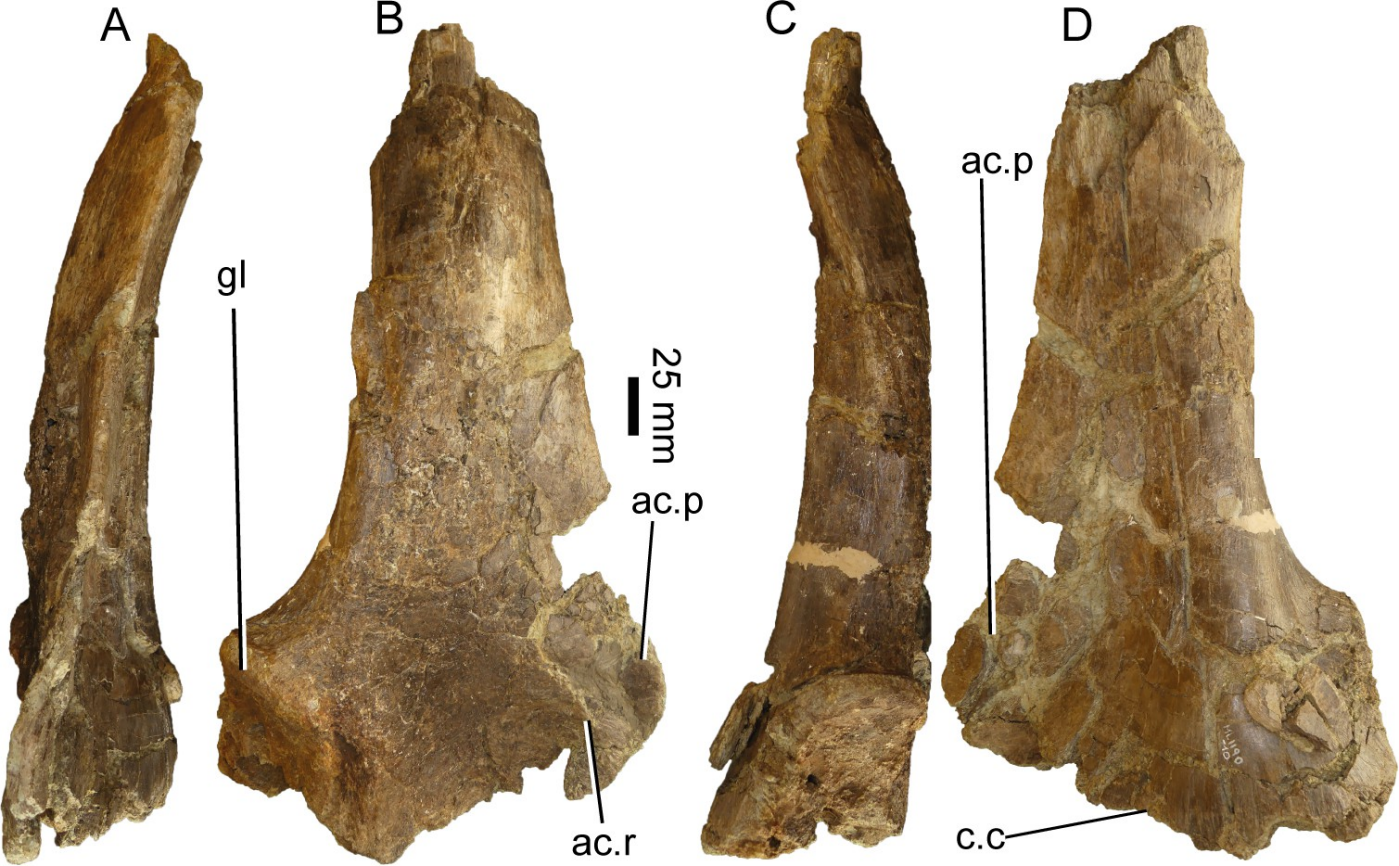
Partial right scapula of *Iberospinus natarioi* (ML1190-10). **A**, anterior, **B**, medial, **C**, posterior, and **D** lateral view. **ac.p**, acromion process, **ac.r**, acromial ridge, **c.c**, contact with the coracoid, **gl**, glenoid.

Only the right scapula is preserved, missing the distal portion. The bone surface is extremely cracked, showing some meteorization, which mainly affects the medial surface (Fig 22).

In the following description, the scapula is oriented with the scapular blade facing dorsally and the coracoid pointing ventrally.

In cross-section, the scapular blade is fusiform (eye-shaped) in outline, being more convex laterally than medially, changing ventrally to a more inverted comma-shaped outline. The anterior rim is continuous from the blade to the acromion, with just a gentle curvature. The acromion itself is not very pronounced from the blade, there is no clear acromial ridge along the lateral surface. This contrasts with the pronounced acromion of *Suchomimus* and the clear angle between the acromion and the blade on other spinosaurids (personal observation). The anterior margin is sharp, which contrasts with the flattened posterior margin of the blade, just dorsal to the glenoid.

The glenoid medial rim is aligned and sub-parallel to the posterior edge of the scapular blade, while the contact with the coracoid is perpendicular to the blade axis. The glenoid surface is facing ventrolaterally (and not ventrally as in *Suchomimus*). The contact with the coracoid is very thick (60 mm) near the glenoid and thin near the acromion (~10 mm). In the most expanded area, the scapula develops the peg-and-notch articulation with the coracoid, in which the scapula projects towards the coracoid ventrally. The contact with the coracoid occupies the entire ventral surface of the scapula, from the glenoid to the tip of the acromion, unlike in *Suchomimus*, where the acromion has a ventral area that lacks such contact (personal observation).

#### Pubis (ML1190-13)

**Fig 23:**
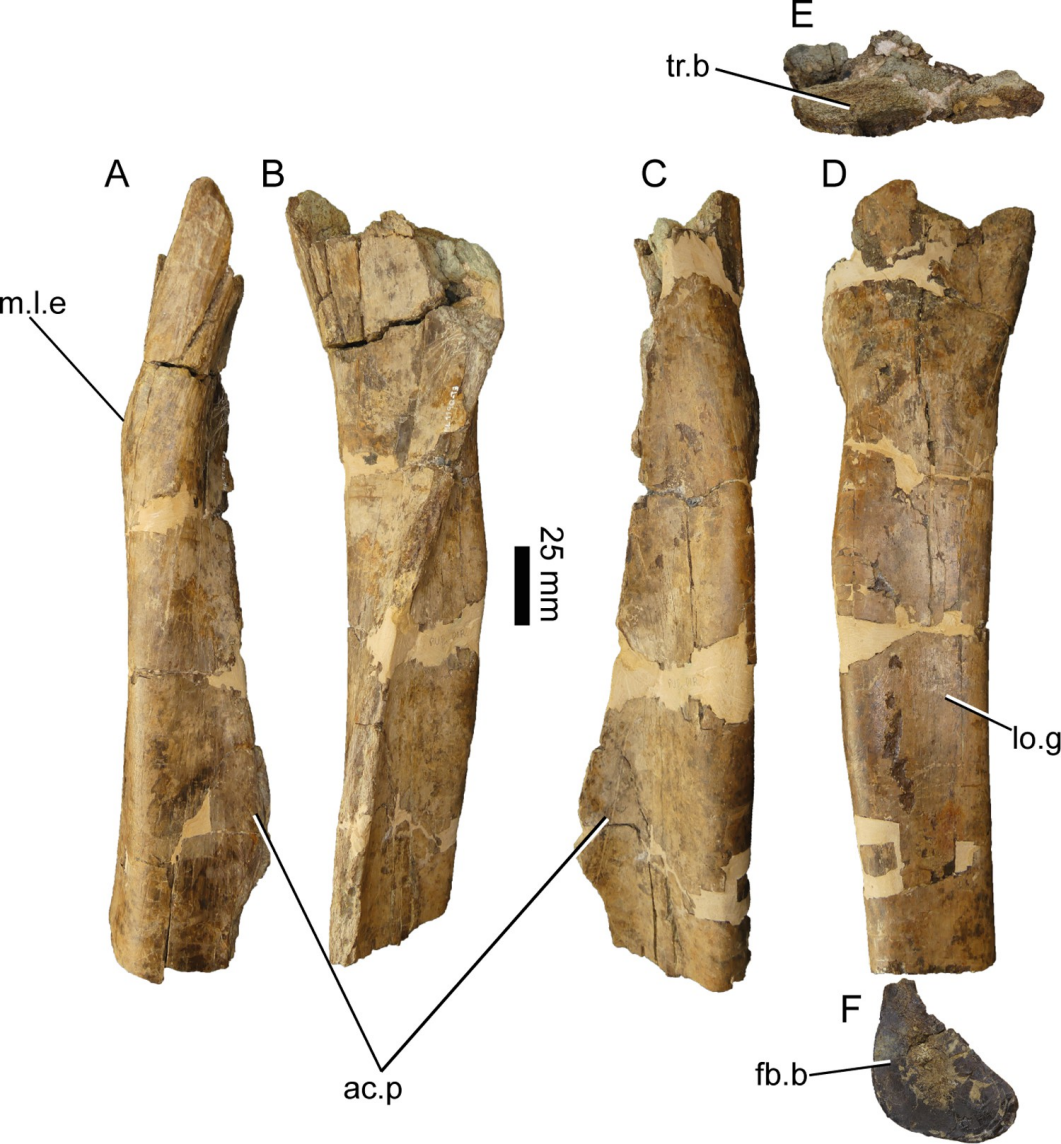
Right shaft of the pubis of *Iberospinus natarioi* (ML1190-13). **A**, anterior, **B**, medial, **C**, posterior, **D**, lateral, **E**, proximal, and **F** distal view. **fb.b**, fibrolamellar bone, **lo.g**, longitudinal groove of the shaft, **m.l.e**, proximal mound like eminence, **p.ap**, pubic apron, **tr.b**, trabecular bone.

The pubis was previously described in [42]. It comprises the shaft of the right pubis, with missing proximal and distal ends, along with the rim of the obturator laminae/pubic apron. Parts of the bone surface have parallel scratches interpreted as fish tooth marks (Fig 23).

Proximally the shaft expands anteromedially to posteroventrally, being compressed transversally (82 and 37 mm respectively). The shaft is overall straight (unlike the slightly more medially curved pubis of *Ichthyovenator*) with a gentle boss on the anterolateral facet of the proximal half of the shaft, referred [42] as a mound-like process, interpreted as interspecific variation and herein interpreted as an autapomorphy. From there subvertical longitudinal grooves occupy its dorso-medial aspect, indicating a muscular insertion. Ventral to said boss, also in anterolateral facet, the shaft is concave, forming a shallow and broad longitudinal groove (potentially autapomorphic as it does not appear in *Baryonyx*, although there is a hint of that feature in the pubis of *Ichthyovenator*).

The rim of the posteromedial longitudinal lamina, pubic apron, is damaged thus preventing an ability to address the entire medial rim of this structure. The pubic apron bears two striking features: 1) the lamina occupies the entire length of the preserved shaft and 2) the lamina runs sinusoidally, from a posterior position proximally to a nearly medial position in the distal end. In both *B*. *walke*ri *and S*. *tenerensis* it is restricted to the first third of the shaft [14], in fact the lamina in ML1190 is thicker distally. This also contrasts with the condition seen in *Ichthyovenator* or even in other theropods like *Aerosteon*, where the lamina only reaches midlength of the pubic shaft [9, 79]. On average the thickness is about 5–10 mm along its preserved length. The outline of the distal end of the preserved portion is comma-like in cross-section.

The proximal bone breakage is mainly occupied by trabecular bone, which contrasts with the distal breaking point, being mostly occupied by fibrolamellar bone (this may represent an osteosclerotic condition).

#### Calcaneum (ML1190-31)

**Fig 24:**
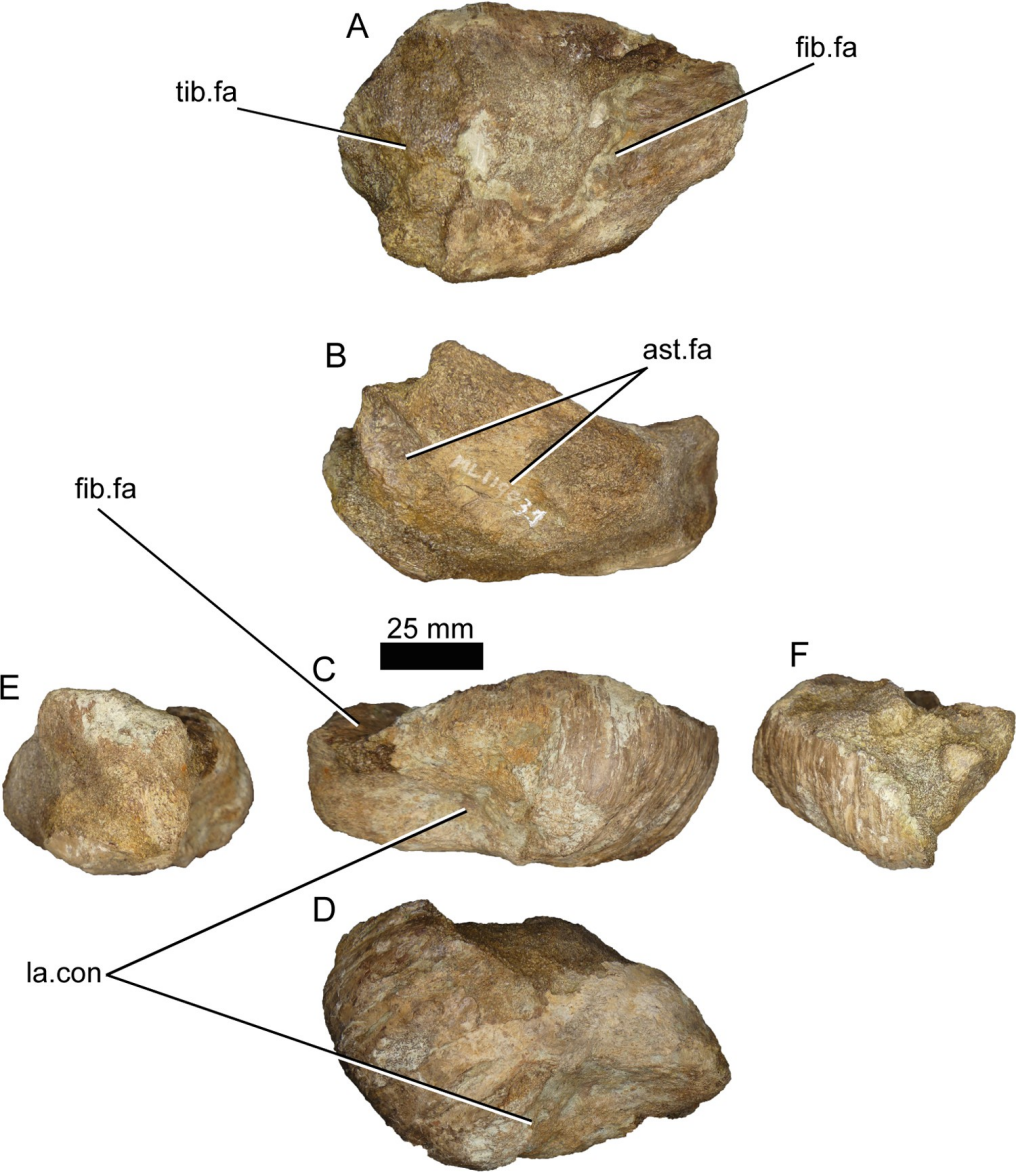
Left calcaneum of *Iberospinus natarioi* (ML1190-31). **A**, proximal, **B**, lateral, **C**, medial, **D**, distal, **E**, anterior and **F** posterior view. **ast.fa**, astragalar facet, **fib.fa**, fibular facet, **la.con**, lateral concavity, **tib.fa**, tibial facet.

Both calcanea are present and are of comparable size, while both astragali are missing. The left calcaneum (ML1190-31) is the better preserved of the two, thus the description is focused on this specimen. The bone is surprisingly massive and stout. The surface is damaged due to erosion making a good description and identification difficult. This fossil was originally figured and interpreted as a right side [42] but it is here reinterpreted as being the left calcaneum (Fig 24).

The calcaneum is anteroposteriorly long (105 mm), nearly twice the transversal width (70 mm), and proximodistally short (45 mm), these proportions might be autapomorphic, since not even in *Baryonyx is t*he calcaneum so proximodistally short while anteroposteriorly long. The distal and distolateral surfaces display conspicuous vertical grooves that accompany the bone curvature (e.g., *Baryony*x) with the surface slightly concave. The medial surface that contacts the astragalus has two astragalar facets, divided by a vertical shallow strut. It has a bigger fibular facet, while the tibial facet is damaged. The fibular facet is twice the size of the conserved tibial facet. Although the distal fibula is not known for any spinosaurid (except in FSC-KK11888, where it is known but the remains are not illustrated nor described) we predict it has a well-developed distal end [16]. The calcaneum is different from any other theropod that we observed, being much more robust.

#### Pedal ungual (ML1190-34)

**Fig 25:**
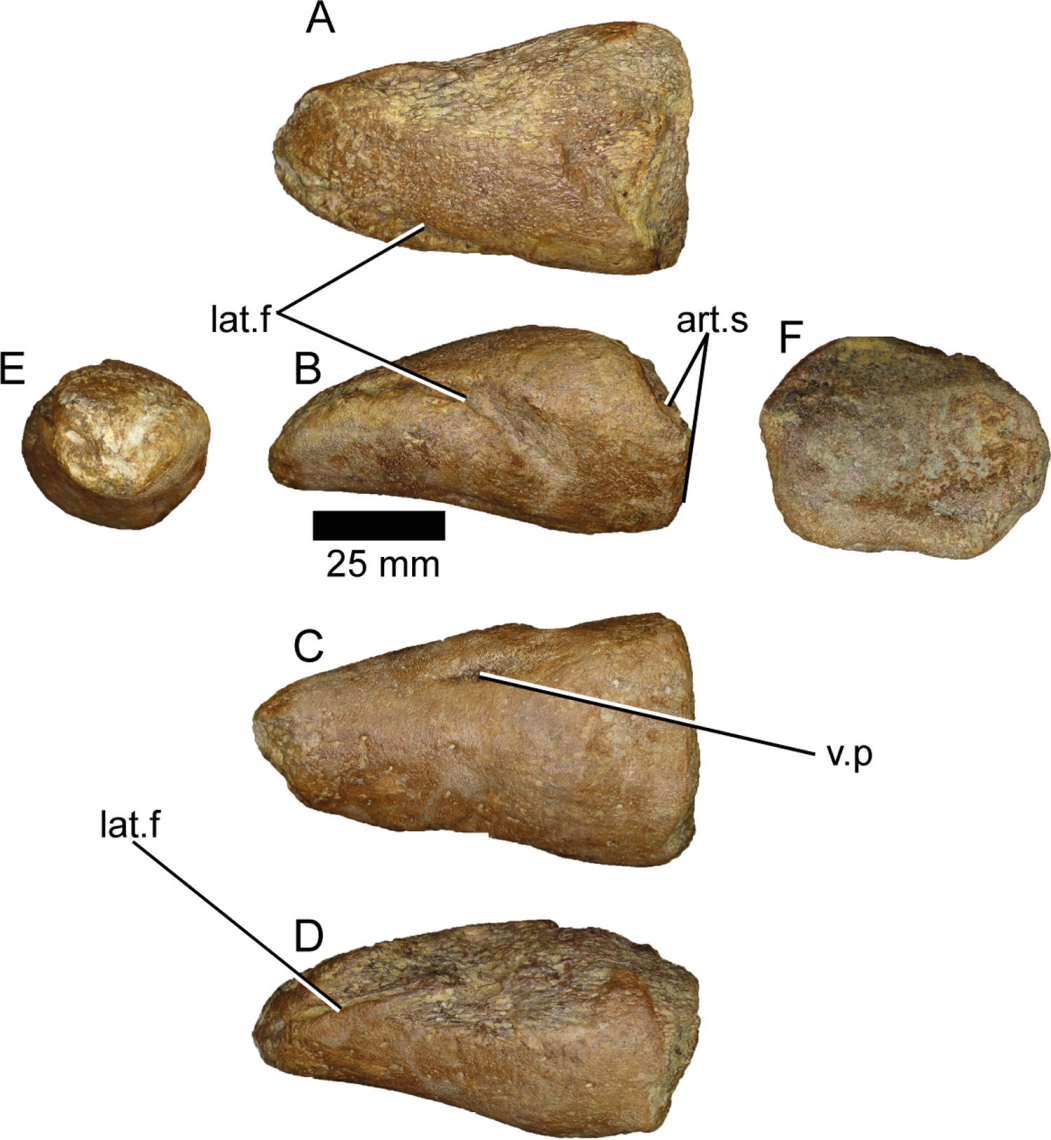
Pedal ungual phalanx of *Iberospinus natarioi* (ML1190-34). **A**, dorsal, **B, D;** lateral, **C**, ventral, **E**, proximal, and **F,** distal view. **art.s**, articular surface, **lat.f**, lateral furrows, **v.p**, ventral pit.

This piece was described by Mateus et al., 2011 [42] therefore herein we will focus on its comparison with other spinosaurid specimens. Measurements of the piece can be found in S7 File. First, it is possible to ascribe the ungual to a pedal phalanx given the wide proximal end, the overall width, and the relatively straight ventral side of the ungual compared to the manual ungueal phalanges present in the *Baryonyx* holotype (Fig 25). ML1190-34 is therefore one of the few known pedal unguals of a spinosaur not referred to *Spinosaurus*, although there are unpublished unguals belonging to *Suchomimus* and an incomplete pedal ungual from the Aptian-Albian of Brazil [80].

Morphologically, the claw does not present the clear two sub-equal articular surfaces described in a juvenile *Spinosaurus* (MSNM V6894) claw by Maganuco & Dal Sasso [81] and by Novas et al., 2005 [82] in an adult one (MPCM 13574). Given the state of preservation of the ventral half of the articular surface compared to the dorsal half it is unlikely that this absence is caused mainly due to erosion. They also lack the large pit situated in the left ventrolateral surface of ML1190-34, which is situated 38 mm from the proximal end and 36 mm from the broken tip of the phalanx. Overall the cross section is oval-shaped, with the dorsoventral axis larger than the mediolateral one compared with the aforementioned specimens belonging to *Spinosaurus*, and therefore ML1190-34 presents a more conical shape cross-section. Another difference is that the lateral furrows seem to be located more dorsally than in *Spinosaurus and* they form simple grooves unlike those of other theropods. They are more similar to those of the Brazil ungual, although the overall shape of ML1190-34 is again more oval-shaped in anterior view, compared with the triangular shape of the previously mentioned phalanx in the same view [80].

In spite of these differences the claw presents spinosaurid features like the almost flat ventral surface, the enlarged proximal and medial mediolateral axis compared with the dorsoventral one and a flexor fossa reaching the borders of the ungual [83].

### Comparative analysis

#### Anterior tip of dentary (ML1190-1)

As previously mentioned it is assumed that only one of the alveoli of the mesial part of the dentary is missing in ML1190-1, whereas there are nine well preserved alveoli. Likewise, given the complete ventral part of the dentary and the still recognizable interdental plates and paradental groove (in certain parts) it is possible to quantitatively compare this piece with other spinosaurid dentaries.

**Fig 26:**
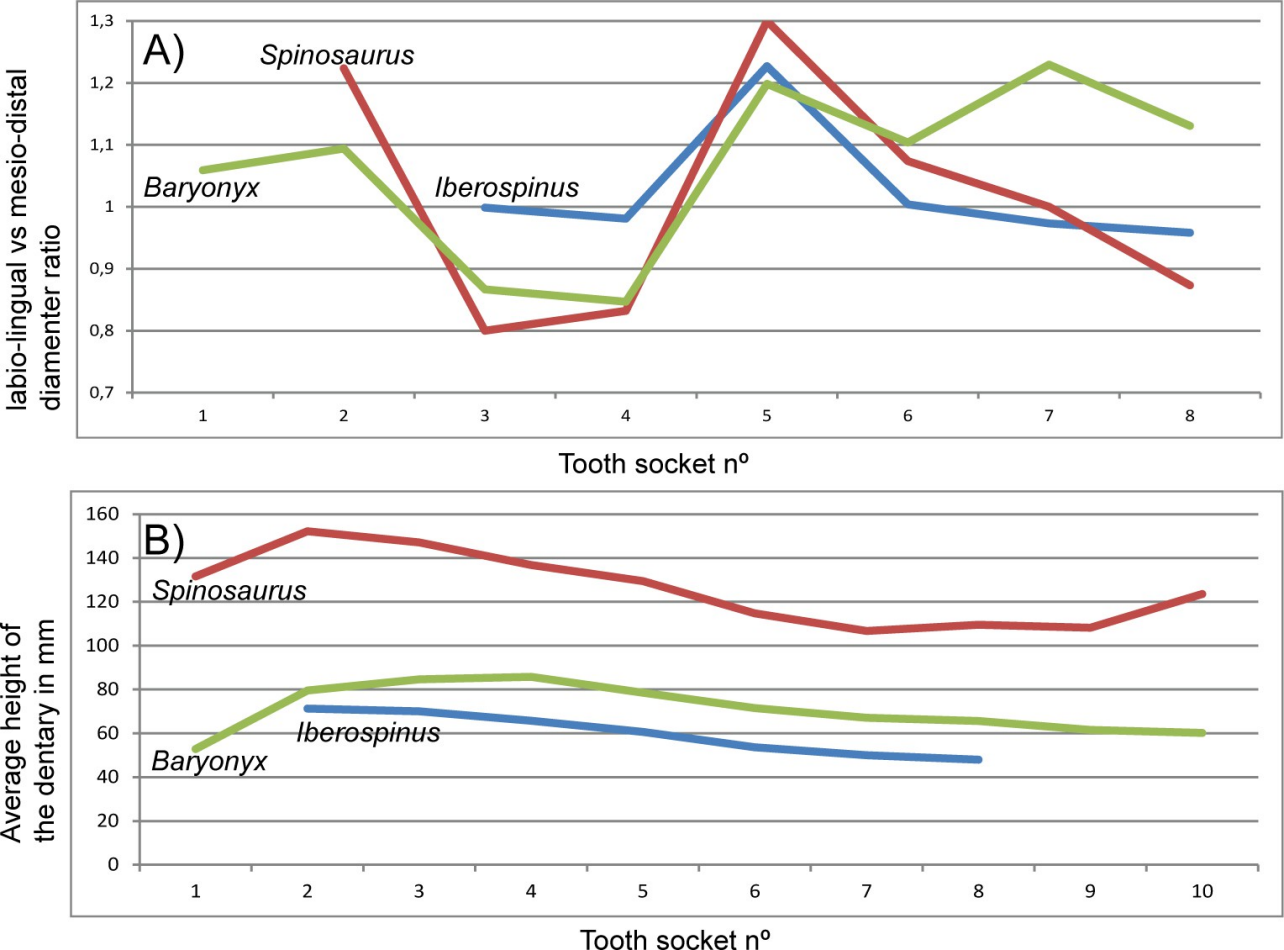
Graphical comparison of the holotype of *Iberospinus natarioi* (ML1190-1) with *Spinosaurus* and *Baryonyx* dentaries. **A**, ratio between labio-lingual and mesio-distal diameters of teeth sockets in the dentaries of ML1190-1, *Baryonyx* holotype (NHMUK VP R9951), and a dentary attributed to *Spinosaurus* (NHMUK VP 16421); **B**, average of lateral and lingual dorsoventral heights of the dentary for each teeth socket of ML1190-1, *Baryonyx* holotype (NHMUK VP R9951) and a dentary attributed to *Spinosaurus* (NHMUK VP 16421); measurements are in mm.

The ratio between the mesiodistal and labiolingual diameters of the teeth alveoli indicates that the third and fourth dentary alveoli of ML1190-1 are more circular than those of *Baryonyx* (NHMUK VP R9951) and a specimen referred to *Spinosaurus* (NHMUK VP 16421) that are more mesiodistally compressed (Fig 26 and S3 File). The 5th alveolus is the most mesiodistally compressed in the three specimens. The alveoli 6, 7, and 8 of ML1190-1 follow a more similar pattern to *Spinosaurus* than to *Baryonyx*.

The average between the height of the dentary in labial and lingual sides for each alveolus indicates that the size of ML1190-1 is the smallest among these specimens and that it follows a more similar outline to *Spinosaurus* than to *Baryonyx* (Fig 26).

#### Isolated tooth (ML1190-3)

After carrying out the PCA analysis; the first principal component (PC1) explains 95% of the variation and is strongly, positively correlated with all measurements except crown base ratio (CBR), to which, it is slightly negatively correlated. The second principal component (PC2) explains 3.5% of the variation and is weakly, positively correlated with crown base length (CBL), CBR and crown base width (CBW); weakly, negatively correlated with crown height (CH) and strongly negatively correlated with crown height ratio (CHR). ML1190 is within the range of variability of *Baryonyx* and slightly within the range of *Suchomimus*. Interestingly the Iberian Baryonychinae fall mostly outside the variability of *Suchomimus* and *Baryonyx* and far from ML1190-3.

**Fig 27:**
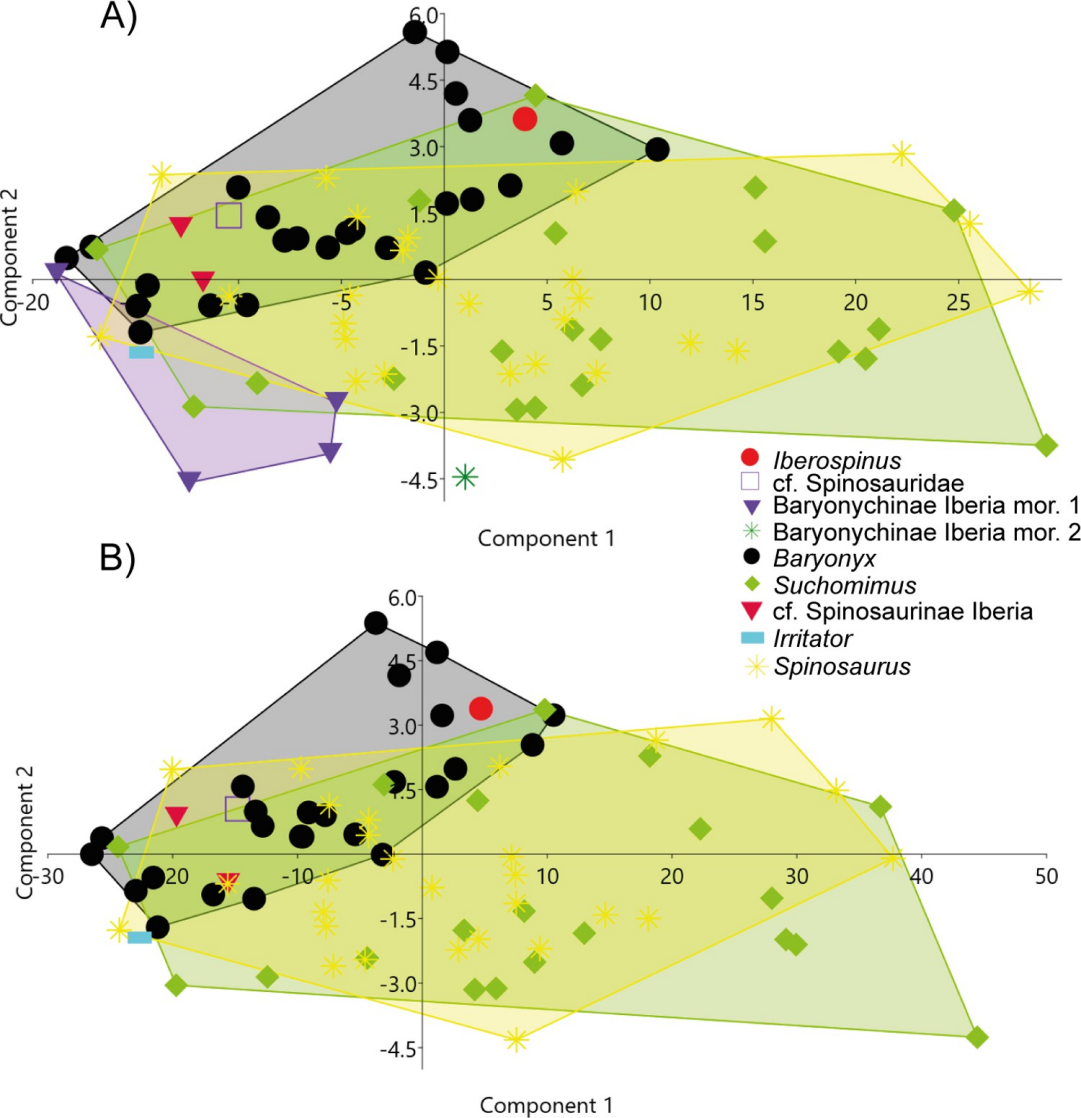
Principal component analyses (PCAs), including several measurements of the tooth of *Iberospinus natarioi* (ML1190-3) with a sample of other Spinosauridae teeth. **A**, First PCA, including Iberian Baryonychinae specimens; **B**, Second PCA, including apical length (AL) measurements and removal of specimens that were missing them in the bibliography. **Red dot**, ML1190-3; **Black dot**, *Baryonyx* teeth; **Blue square**, possible basal Spinosauridae from Niger; **Crimson inverse triangle**, possible Iberian Spinosaurinae; **Green diamonds**, *Suchomimus* teeth; **Violet inverse triangles**, Iberian Baryonychinae morphotype 1; **Green star**, Iberian Baryonychinae morphotype 2; **Blue dash**, *Irritator* tooth; **Yellow stars**, *Spinosaurus* teeth.

In the second analysis (Fig 27B), the apical length (AL) data was included, therefore specimens missing this information were removed from the PCA. The PC1 explains 97% of the variation and is strongly positively correlated with all measurements except CBR, to which it is slightly negatively correlated. The PC2 explains 1.5% of the variation and is weakly positively correlated with CBL, CBR and CBW; weakly negatively correlated with CH and AL and strongly negatively correlated with CHR. ML1190-3 falls again within the variability of *Baryonyx*, but outside the variability of *Suchomimus*.

#### Pedal ungual (ML1190-34)

Unfortunately, there are not many publications describing pedal phalanges attributed to spinosaurids aside from the two previously mentioned [81, 82] and the specimen FSAC-KK 11888 [15]. The data of the holotype of *Dilophosaurus* (UCMP 37302) was included in the analysis as a control, being an earlier branching theropod compared with derived spinosaurids [63]. The ungual phalanx of an abelisaur from Kem Kem and the ungual phalanx of *Tyrannotitan chubutensis* were also included in this analysis for further refining morphometric characterizations of the ungual ML1190-34; the measurements of the specimens appear in S7 File.

**Fig 28:**
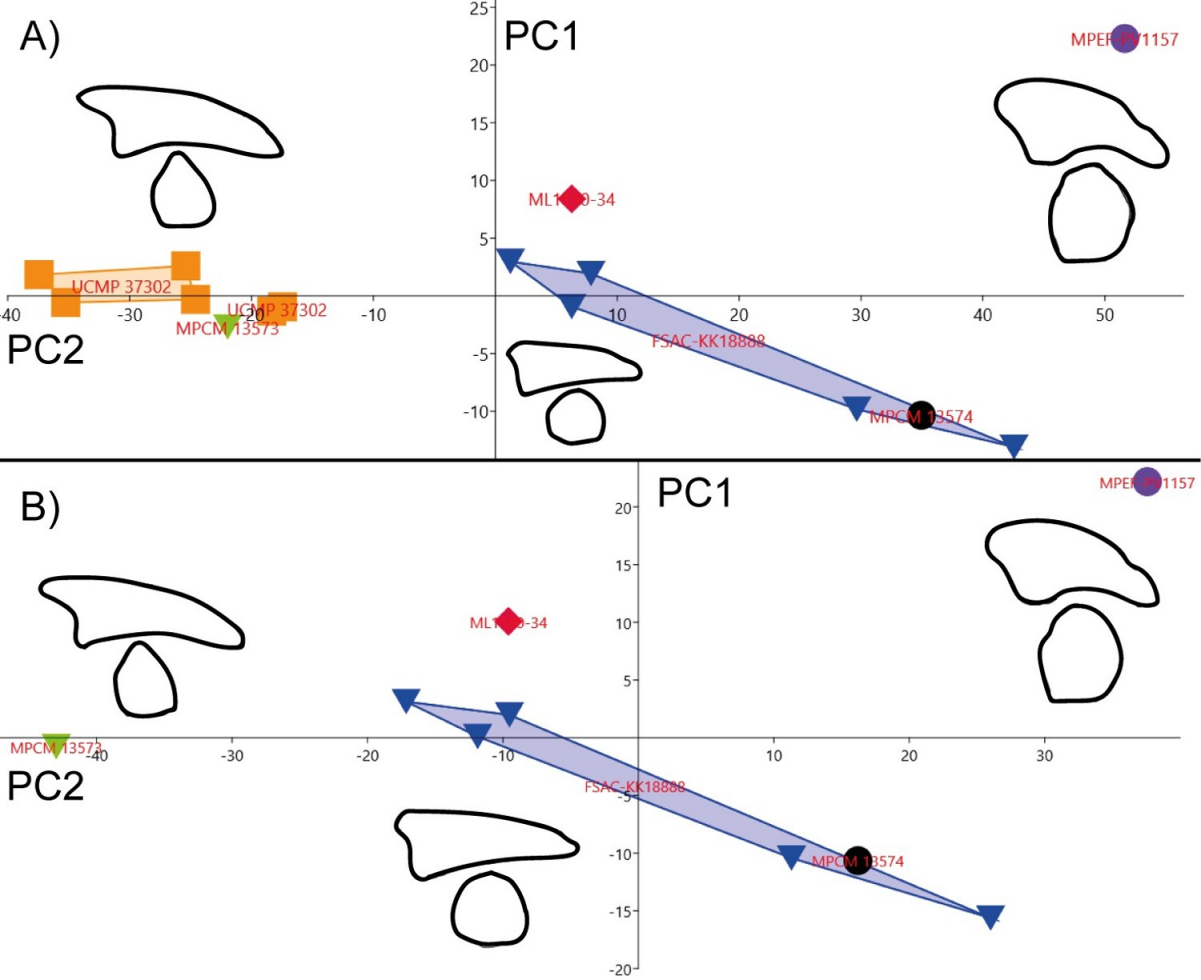
Principal component analyses (PCAs) including the ungual of *Iberospinus natarioi* (ML1190-34) compared with other theropods. **A**, limited PCA, not including the mid-length diameters of unguals; **B**, complete PCA, including all available measurements for the selected taxa. Silhouettes based on Novas et al., 2005 for *Tyrannotitan* and Abelisauria indet., and Maganuco & Dal Sasso, 2018 for *Spinosaurus*.

Results of the PCA show three clusters of specimens when the total length, the mediolateral and dorsopalmar diameters are considered (Fig 28). 1) *Dilophosaurus* and the abelisaur of Kem Kem, with low values in PC1 and medium values in PC2; 2) ML1190-34 and the spinosaurids of Kem Kem, with medium to high values in PC1 and medium to low values in PC2; and 3) *Tyrannotitan* isolated with high values in both PC1 and PC2. This scenario does not change when the midlength diameters of the unguals are included. ML1190-34 is thus most morphometrically similar to the ungual phalanx of the fifth digit of FSAC-KK 11888.

**MPCM 13574**, *Spinosaurus* ungual phalanx, black dot; **FSAC-KK18888**, relatively complete foot of *Spinosaurus*, inverted blue triangle; **UCMP 37302**, *Dilophosaurus*, orange squares; **MPEF-PV1157**, *Tyrannotitan* ungual phalanx, violet dot; **MPCM 13573**, Abelisauridae indet. ungual phalanx, green inverted triangle.

**Fig 29:**
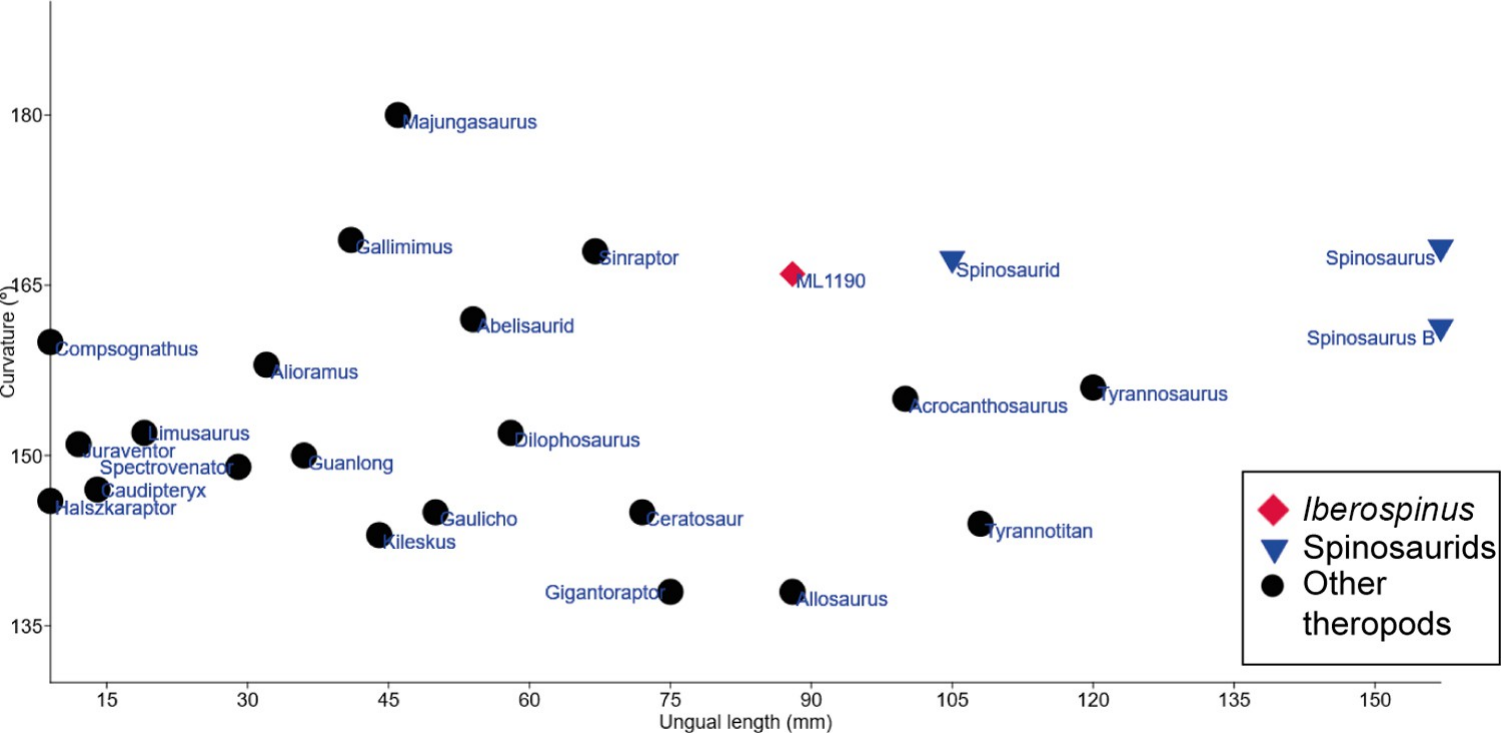
Curvature vs length of the ungual of *Iberospinus natarioi* (ML1190-34) compared with other theropods. ML1190-34, red diamond; **Spinosauridae**, inverted blue triangles; **other theropods**, black dots. Curvature is in degrees and length is in mm.

If we include the data of ML1190 into the dataset by Hone & Holtz, 2021 [17], that measures the ungual curvature vs its length (Fig 29), we can see that the closest specimen is MPCM 13574, classified as a *Spinosaurus* or a closely related form (spinosaurine) by Novas et al., 2005 [82].

#### Caudal vertebrae

The tail anatomy in spinosaurids is poorly known compared with other groups of dinosaurs. The 15 caudal vertebrae (12 with the centrum at least mostly complete and 3 fragmented) known from ML1190 make it one of the most complete caudal series of any known spinosaurid. The striking adaptations appearing in the tail of some of them, potentially related with aquatic locomotion, raises the question of the tempo and mode of this adaptation in this unique group of theropods [16, 84]. Being relatively old for a spinosaurid, ML1190 can offer insights into this question, even when there is not one single, complete vertebra recovered.

In order to properly determine the sequence of vertebrae (as they were found only in association and not articulated) we first calculated the surface in square millimeters of the anterior centrum facet and we divided this value by the square length of the centrum. We name this metric the Anterior Surface Index (ASI). The anteriormost vertebra of the sequence was determined on the basis of qualitative criteria and the others were ordered on the basis of the percentage of the ASI of the first vertebra that they present. As previously mentioned the tails of a more typical theropod (e.g., *Dilophosaurus*) [63] and the mostly complete tail of FSAC-KK 11888 (*Spinosaurus*) [16] were used as templates (See S6 File).

Another important clue to determine the position of the vertebrae in the tail was the presence of a diapophysis. The posteriormost point in the caudal sequence where the diapophysis appears in *Dilophosaurus* is the 17th vertebra [63], in *Spinosaurus* it is between the 16th and 19th [16], in *Concavenator* is around the 19–20 [85]. In other large theropods the point where the diapophyses disappear ranges between the 25th vertebra in *Majungasaurus* and the 12th in *Gorgosaurus*, with *Tyrannosaurus* at the 17th [64].

**Fig 30:**
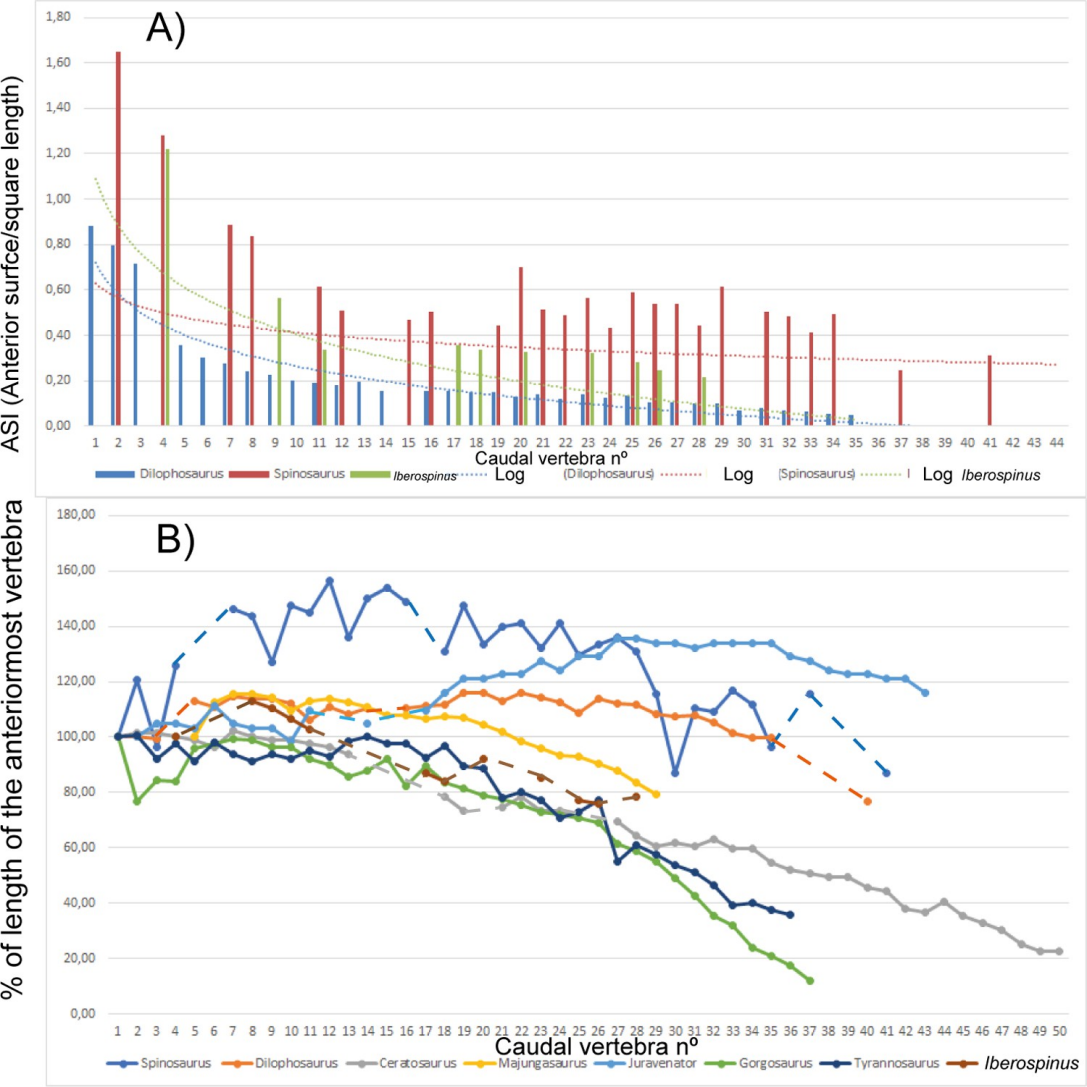
Metrics of the caudal vertebrae series of *Iberospinus natarioi* (ML1190) compared with other theropods. **A**, ASI/square length for each vertebra; **B**, percentageof the length for each caudal vertebra compared with that of the anterior-most preserved vertebra.

When we compare side-by-side the percentages of ASI from the first vertebra onwards of the caudal series of *Spinosaurus* and *Dilophosaurus*; we can see that the vertebra with diapophyses in *Dilophosaurus* range from 90% to 17%; in *Spinosaurus* they range between 97% to 29%, meaning that the tail vertebrae of the latter maintained their size more stable posteriorly in the overall sequence, an adaptation that might be related to the attachment of bigger muscles for swimming (Fig 30). In ML1190, ML1190-15 is interpreted as the fourth vertebra of the caudal series, on the basis of the ASI compared with *Spinosaurus*. The caudal vertebra with diapophysis of the ML1190 series displays similar values to *Spinosaurus*, with the last vertebra presenting diapophysis (ML1190-26) being 29% the ASI of the first one. A position of vertebrae is also given on the basis of the % of ASI and their qualitative features.

For the length of the caudal vertebrae, we examined the data from [64] for the big theropods *Dilophosaurus*, *Majungasaurus*, *Ceratosaurus*, *Gorgosaurus*, and *Tyrannosaurus* and added the spinosaurids *Spinosaurus* (FSAC-KK 11888) [16] and ML1190. *Juravenator* was also included in the analysis, given that it has been suggested to have a lifestyle similar to the one proposed by some authors [17] for *Spinosaurus* (wading predator) and this could be an interesting comparison [86]. Once again, we look at the percentage of length with respect to the first caudal, or the most anteriorly known vertebra in the series, for each vertebra. The resulting pattern (Fig 30) highlights the relative similarities between *Juravenator* and *Spinosaurus* (they are the taxa with the longest tail vertebrae with respect the first one) although they have the longest vertebrae in different parts of the tail, more anteriorly in *Spinosaurus* and more distally in *Juravenator*. Nevertheless, the pattern inferred for *Iberospinus* seems to be more comparable with other theropods like *Gorgosaurus* or *Ceratosaurus*.

### Phylogenetic analysis

**Fig 31:**
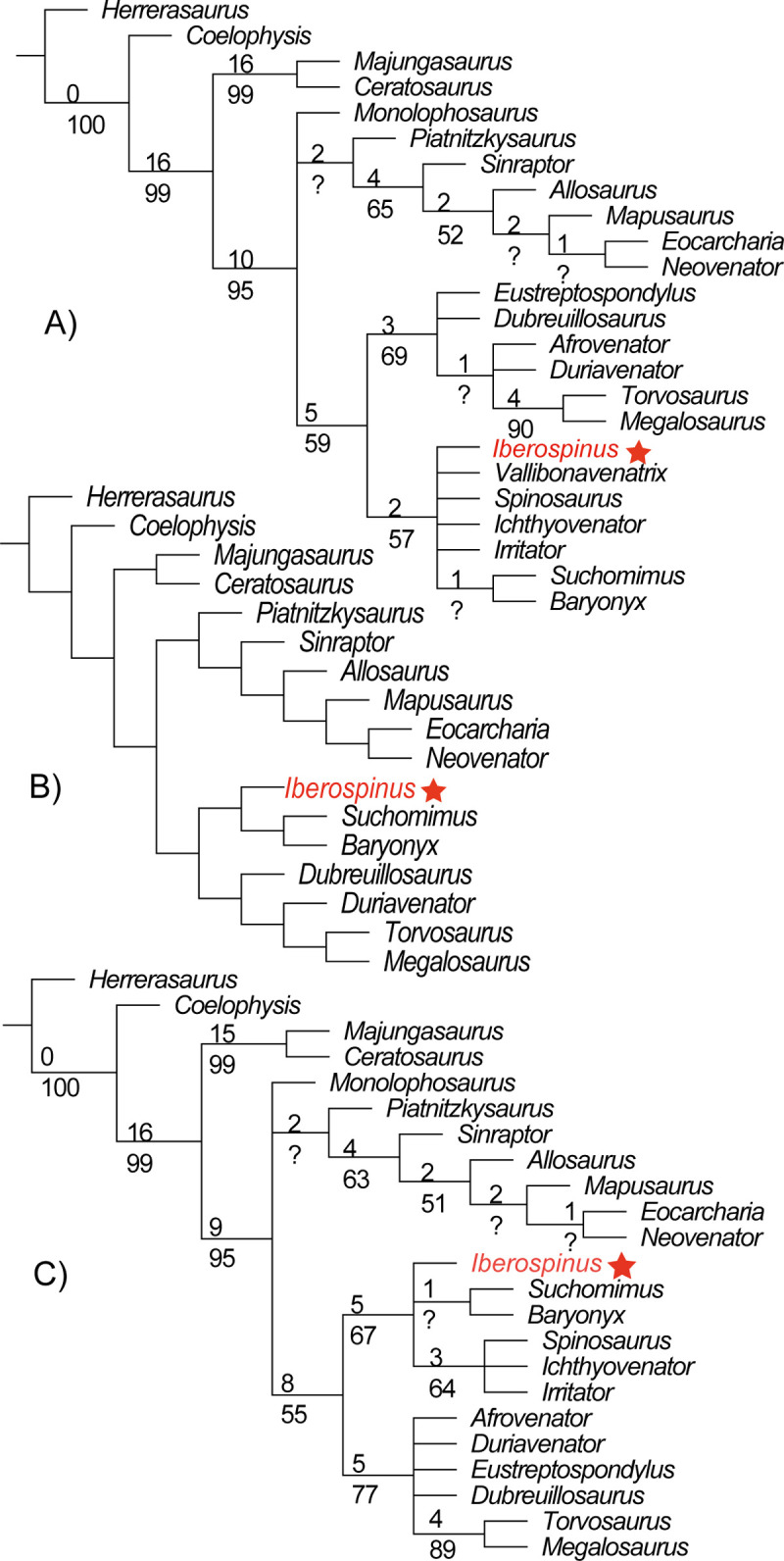
Cladograms including *Iberospinus natarioi* (ML1190) and other 23 theropods, generated with the software TNT 1.5 (No taxon limit). **A**, Strict consensus tree including all the taxa in the analysis; **B**, Agreement subtree; **C**, strict consensus tree pruning the taxon *Vallibonavenatrix cani*. **Bremer support** for the node is shown above the branch; **Bootstrap value** for the node is below the branch.

Results of the cladistic analysis performed on the data matrix yielded 48 most parsimonious trees (MPTs) with lengths of 1035 steps. The strict consensus has a Consistency Index (CI) of 0.568 and a Retention Index (RI) of 0.566 (Fig 31A). The analysis shows a polytomy within Tetanurae formed by *Monolophosaurus* plus Allosauroidea including *Piatnitzkysaurus*, as recovered by Rauhut & Pol [23]. However, the node is not well supported (both in bootstrap value and Bremer support), with only a more traditional Allosauroidea including *Sinraptor*, *Allosaurus*, and their common ancestor with all its descendants being well supported in our analysis. The third part of the polytomy is Megalosauroidea, being also well supported in our analysis. Within Megalosauroidea we recover the division between Spinosauridae and Megalosauridae as well, with the latter being better supported than the former. Within Megalosauridae the only node well supported is the one that links *Megalosaurus* and *Torvosaurus*. Within Spinosauridae we recovered a polytomy, albeit not well supported, a restricted Baryonychinae including *Baryonyx* and *Suchomimus* is recovered. In this first analysis the synapomorphies of Spinosauridae include both the presence of centrodiapophyseal lamina (Character 180) and a deep infraprezygapophyseal fossa in dorsal vertebrae (Character 181). There are no unambiguous synapomorphies for Baryonychinae in the tree but there are some ambiguous ones: the smaller and more numerous dentary teeth compared with the maxillary teeth (Character 153), an ischium with a notch ventral to the obturator process (Character 293), an anterior tip of the dentary strongly curved upward in lateral view (Character 382), second premaxillary tooth significantly smaller than the third (and fourth) tooth (Character 397), and more than 25 dentary teeth (Character 413). The agreement subtree (Fig 31B) prunes all taxa within Spinosauridae except ML1190, *Baryonyx* and *Suchomimus*. In this second analysis a local autapomorphy recovered for ML1190 is the presence of a single Meckelian foramen in the dentary (Character 124).

If *Vallibonavenatrix* is pruned from the third analysis, a well-supported Spinosaurinae with a polytomy formed by *Spinosaurus*, *Ichthyovenator*, and *Irritator* is recovered (Fig 31C). The synapomorphies of the clade include: a reduced curvature of the teeth crowns (Character 140), maxillary and dentary teeth without denticles (Character 145), posterior dorsal vertebrae with spines posteriorly inclined (Character 190), dorsal vertebrae with spines more than twice as tall as the centrum (Character 191) and neural spines with dorsal extension above the level of the intraspinous ligaments (383).

## Discussion

Currently, despite the lack of relatively complete material (a problem common in the group overall), the record of spinosaurid material from the Iberian Peninsula is one of the best in the world [13, 30]. *Iberospinus* increases the large spinosaurid diversity from Iberia despite the fragmentary nature of its remains and it is currently one of the most complete spinosaurid specimens in the world; with more material attributed to it than to the holotypes of *Cristatusaurus*, *Oxalaia*, *Sigilmassasaurus*, *Ostafrikasaurus*, *Siamosaurus*, *Angaturama* or *Suchosaurus* [13, 27]. The occurrence of more than one spinosaurid taxa in a relatively restricted area (like Iberia) seems to be a common situation, as it likely happens in the Cenomanian of North Africa for example [25–27] or in the Barremian of South England [7].

Regarding its phylogenetic placement, we recovered ML1190 in a similar position as the one recovered by Arden et al., 2019 [44], with the characters in the dentary, pubis, and caudal vertebra allowing us to differentiate ML1190 from both *Baryonyx* and *Suchomimus*. *I*n agreement with [30] on the basis of dental and vertebral characters (*i*.*e*., small denticles in teeth and longer than tall dorsal vertebrae), we favor an affinity between *Iberospinus* and the baryonychines rather than the spinosaurines. In a similar manner we could not recover *Vallibonavenatrix* as a member of Spinosaurinae, but characters in its vertebrae (*i*.*e*., the height of the neural spines and neural spines with dorsal extension above the level of the intraspinous ligaments) might point towards an affinity to spinosaurines, rather than with Baryonychinae. It is also worth mentioning that the scenario recovered by our phylogenetic analysis is relatively similar to that recovered with the use of geometric morphometrics on the premaxilla of spinosaurids, with a “restricted” Baryonychinae including *Suchomimus* and *Baryonyx* [87].

The phylogenetic position of other Iberian spinosaurids needs to be addressed. Currently the position of *Camarillasaurus* could not be resolved beyond its likely inclusion in Spinosauridae [8, 41], therefore it is not possible to assign it to either spinosaurid clade, although the differences between it and *Vallibonavenatrix* might point towards a non-spinosaurine position [30]. Nevertheless other characters, like the pachyostosis in the tibia [8], might point towards an affinity with spinosaurines as some phylogenies have pointed out [7]. Only new material from *Camarillasaurus* would confirm its placement with greater detail in the spinosaurid family tree [7]. The material traditionally assigned to *Baryonyx* from the Iberian Peninsula, as the specimen from Sala de Los Infantes [35], needs revision in light of this recent work that suggests a complex scenario between the Iberian spinosaurids in general and the baryonychines in particular [37, 39]. Although as previously shown, isolated elements such as claws, teeth and appendicular bones already hinted at a hidden diversity within this clade. This complex scenario between the baryonychines is further highlighted by the quantitative analysis of the tooth of ML1190-3, falling it far from other Iberian animals assigned to the clade, like the ones presented by Isasmendi et al., 2020 [66] and within the range of variation of *Baryonyx* and *Suchomimus*. Meanwhile, the outline of the dentary itself is more similar to *Spinosaurus* than to the one of *Baryonyx*.

The quantitative analysis of the tail vertebrae of ML1190 reveals that the anterior centrum surface area follows a relatively similar pattern as *Spinosaurus*. Meanwhile, the length of ML1190 tail vertebrae with respect to the anteriormost known vertebra seems to follow a pattern akin to that of dinosaurs with more generic tails. It is interesting to note that *Juravenator* (an animal in which sensitive scales similar to those of crocs have been found) has the most similar pattern to *Spinosaurus*, that might point towards aquatic adaptations compatible with the model of a wading predator [16, 64, 86]. This result is even more interesting if we consider the megalosauroid affinities that have been proposed for *Juravenator* [88].

Several morphometric parameters of the pedal ungual phalanx show an intermediate condition between those of theropods with typical terrestrial lifestyles and *Spinosaurus*. This coupled with the previously discussed characteristics of the tail, point towards a relatively expected paleoecology for *Iberospinus* as a baryonychine, not having the extreme adaptations (either for wading or pursuit predation) seen in some spinosaurines, but presenting a tendency towards those [16, 84].

Regarding the inner anatomy of ML1190-1, the lack of a significant number of studies of this type in the dentary (usually they are centered around the premaxilla and maxilla of spinosaurids or other Mesozoic reptiles [89, 90], as complete dentaries are not common even in other theropods [91]) precludes us from reaching significant conclusions that might have systematic significance within the Spinosauridae group itself. Compared with the replacement pattern seen in *Tyrannosaurus* one of the biggest differences is the amount of secondary replacement teeth, both in the first five alveoli and further back in the mandible. In a specimen of *Tyrannosaurus* only one secondary replacement tooth is present in the fourth alveolus of the right dentary [92], and in *Australovenator* no secondary replacement teeth appear, with even some alveoli lacking replacement teeth altogether [93]. The presence of numerous replacement teeth compared with other theropods points towards a faster replacement rate of teeth, as seen in *Spinosaurus* [94], and indicates that this adaptation, which could have been related to piscivory, dates back to the early evolution to the clade. The extent of the secondary replacement teeth in the anterior alveoli of the dentary means that the condition proposed by Hendrickx et al. [29] as synapomorphic for Spinosauridae (“Dentary rosette of four teeth’’) can be rephrased as “Dentary rosette of at least four teeth’’ or “Dentary rosette with four alveoli”.

Regarding the neurovascular anatomy, the great amount of vascularization contrasted with the relative lack of it on *Australovenator* [93]. Other studies [95] have shown the appearance of complex neurovascular nets in the dentaries of theropods not closely related with the spinosaurids. The dentary of *Tyrannosaurus* presents a comparable neurovascular system, albeit with some differences in the shape of the channels that might point to adaptative differences. The mandibular branch of the trigeminal nerve in *Tyrannosaurus* points dorsally, almost 90°, meanwhile in *Iberospinus* presents a gentler sloping angle. Although generally comparable in complexity, the neurovascular channels in *Tyrannosaurus* present more branches both in the occlusal part of the mandible (next to each alveoli) and in the venteral part, with two rows of channels in the area instead of just one row like in *Iberospinus*. Meanwhile *Iberospinus* presents more foramina and channels pointing directly to the lateral part of the dentary.

Finally, given the probable European origin of megalosauroids [96] and that most of the earliest known spinosaurids (Barremian) come from Western Europe in general [7] and the Iberian Peninsula in particular [2]; the inclusion of *Iberospinus* makes a probable European origin of the clade even more likely. Despite this, given the protracted time period (~30 million years) in which there are no undisputed spinosaurid fossils, except maybe some potential spinosaurid teeth and a possible claw [2, 4], this European origin cannot be confirmed until the discovery of unquestionable Jurassic representatives of the group in the region.

## Conclusions

**Fig 32:**
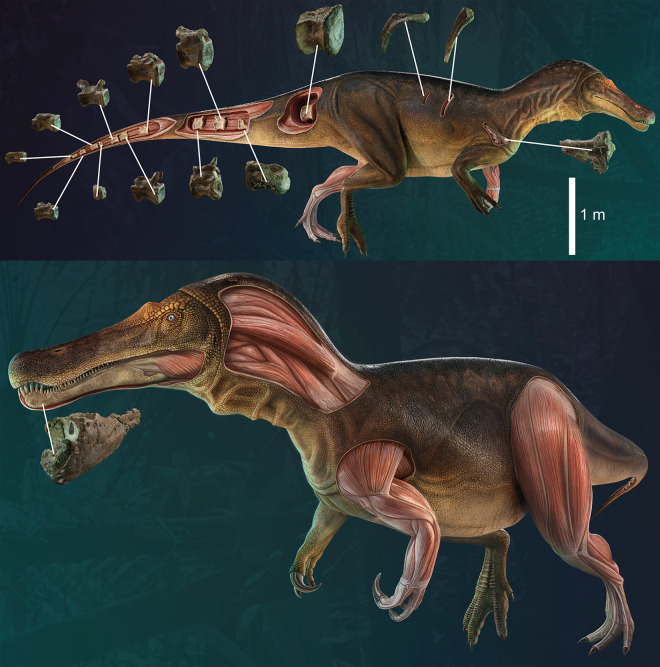
Life reconstruction of *Iberospinus natarioi* (ML1190) showing 3D digitalization of some of the recovered bones, along with reconstructed musculature. **Upper**, right lateral view; **Bottom**, anterolateral left view. Paleoart by Victor Feijó de Carvalho.

The revision of Barremian spinosaurid material previously assigned to *Baryonyx* along with newly recovered material from the site has led to the description of a new genus and species of Iberian spinosaurid, *Iberospinus natarioi*, from the Papo Seco Formation of Cabo Espichel, Sesimbra, Portugal. The specimen includes: dentary, isolated teeth, scapula, ribs, a dorsal vertebra, neural arches, pubic shaft, 15 caudal vertebrae, calcanea, and one pedal ungual (Fig 32). It is a medium sized spinosaurid diagnosable by: the dentary with only one foramen in the Meckelian sulcus and a straight ventral edge (not upturned), the presence of laminae in the pleurocelic depression of the medio-distal caudal vertebrae, the straight anterior rim of the scapula (acromion not protruding); the reduced acromial ridge of the scapula and the contact with coracoid occupying the entire ventral surface of it; the pubic apron being thick in almost the entire length of the pubis shaft, and the presence of a mound-like eminence in the proximal lateral part of the pubis. Phylogenetic analysis recovers *Iberospinus natarioi* outside of the clade formed by *Suchomimus* and *Baryonyx*, although other characters (like the teeth denticles) point towards an affinity with baryonychines. The morphometric analysis of several anatomical elements points towards an ecology similar to the expected for a basally branching spinosaurid. The addition of yet another taxon to the diversity of spinosaurids in Iberia besides *Vallibonavenatrix*, *Baryonyx*, and *Camarillasaurus* indicates that the clade possibly originated in Western Europe.

## Supporting information

S1 File Character matrix. (TXT)

S2 File Character matrix. (TNT)

S3 File Dentary measurements & dataset. (XLSX)

S4 File Dental measurements & datasets. (XLSX)

S5 File Extended description of caudal vertebrae. (PDF)

S6 File Vertebrae measurements & datasets. (XLSX)

S7 File Pedal ungual phalanx measurements & datasets. (XLSX)
