# Supplementary material for: A new theropod dinosaur from the early cretaceous (Barremian) of Cabo Espichel, Portugal: Implications for spinosaurid evolution
Source: PLoS One. 2022 Feb 16;17(2):e0262614. doi: 10.1371/journal.pone.0262614 (PMC8849621; doi:10.1371/journal.pone.0262614)
Supplement: S5 File — (PDF) [file pone.0262614.s005.pdf]

EXTENDED DESCRIPTION OF CAUDAL VERTEBRAE

ML1190-15

This vertebra is interpreted as the anteriormost and the largest of the caudal vertebrae of ML1190, that was already described by (1). It comprises a well preserved centrum lacking the neural arch, which presents some eroded surfaces mostly in the posterior facet (Fig S1).

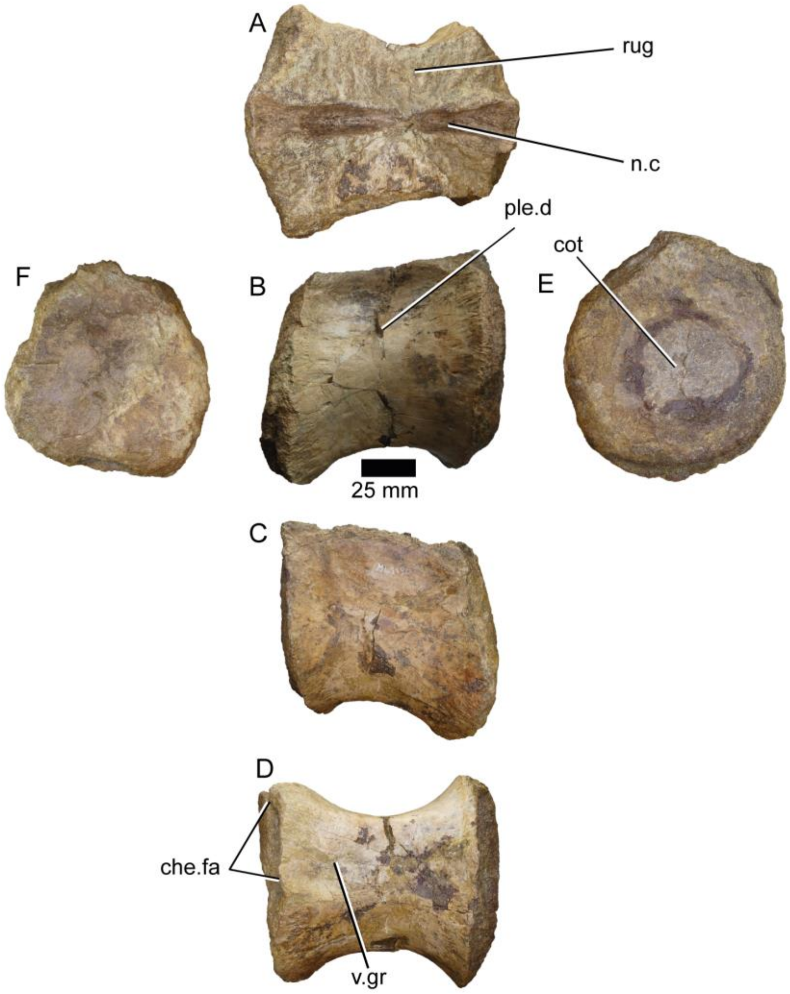

Fig S1. Caudal vertebra of *Iberospinus natarioi* (ML1190-15)

A, Dorsal, **B,C**, lateral, **D**, ventral, **E**, anterior and **F** posterior view.

cot, cotile, **rug**, rugosities, **che.fa**, chevron facets, **ple.d**, pleurocelic depression, **v.gr**, ventral groove, **n.c**, neural canal

The vertebra is roughly as tall as long. The anterior facet is concave and slightly oval, with an anterior cotile of about 7 mm of diameter in the middle.The anterior end of the neural canal extends anteriorly over the concave surface of the centrum. The area where the neural arch was attached to the centrum is extremely rough. This rugose area is wider mediolaterally in the anterior portion of the neural canal. The neural canal is hourglass shaped, wider mediolaterally at both anterior and posterior ends (although is slightly wider in the anterior end). It has some ridges running anterodistally across its ventral surface. From both ends it tapers medially towards a point slightly offset to the posterior part of the centrum, where it reaches its minimal diameter, of only about 6 mm wide. This point is also the shallowest point of the neural canal into the centrum.

The anterior part of the lateral surface of the vertebral centrum is slightly higher dorsally than in the posterior part. There is a shallow pleurocentral depression in the dorsal half of the centrum, but there are not any signs of pleurocoels. Both posterior and anterior ends of the lateral surface present some foramina and rugosities, being especially marked in the posterior ventral and anterior dorsal areas.

Ventrally the vertebra presents a clearly marked groove in the midline, whose edges end posteriorly in the chevron facets. The grove is more apparent in the posterior part of the vertebra; meanwhile it almost disappears in the anterior part. There is a small protuberance or bulge in the medial part of the groove, on its posteriormost part.

The posterior facet is mostly flat (although it is the most damaged part of the vertebra) without any signs of cotile and is about as oval as the anterior facet.

ML1190-17

This vertebra is one of the most damaged ones of the vertebral series. The neural arch is completely missing, the right anterior part of the centrum totally disappeared and the rims of both central facets are highly eroded (Fig S2).

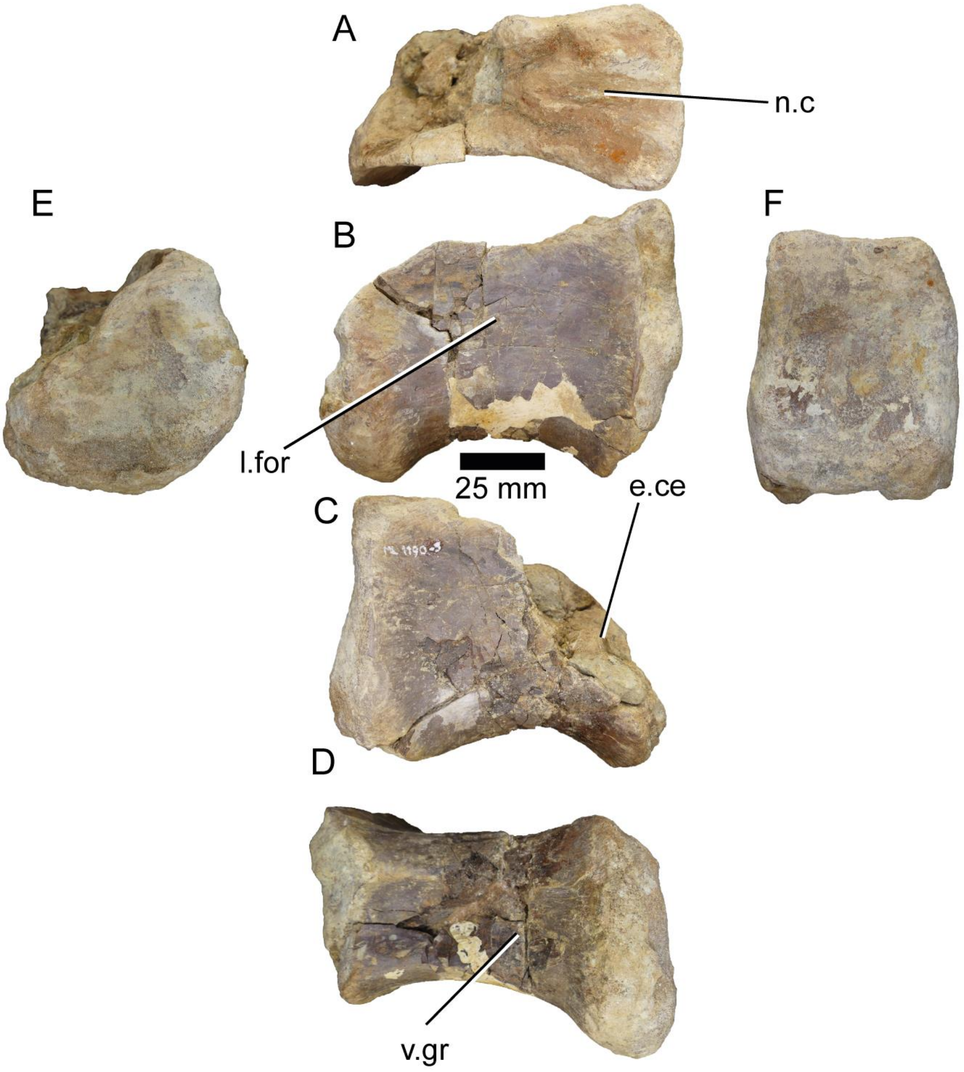

Fig S2. Caudal vertebra of *Iberospinus natarioi* (ML1190-17)

A, Dorsal, **B,C**, lateral, **D**, ventral, **E**, anterior and **F** posterior view.

n.c, posterior part of the floor of neural canal, **l.for**, lateral foramen, **e.ce**, eroded portion of centrum, **v.gr**, ventral groove.

Nevertheless, it is still possible to see the posterior part of the neural canal, whose edges tapper medially towards the middle of the vertebra, as well as ventrally as the canal goes

deeper into the centrum. The ventral half of the posterior facet is straight, meanwhile the dorsal half is slightly concave. It possesses a small foramen of about 2,5 mm in diameter in the dorsal half of the left side of the centrum, more or less in the middle between anterior and posterior facet rims.There is a clear groove running ventrally across the midline of the centrum, which almost disappears in the anterior part. Both edges that limit it dive more pronouncedly towards the groove in the posterior part.

ML1190-16

This vertebra was already described by (1) although briefly, and was interpreted as mid-anterior caudal. It possesses the neural arch, although it lacks the transverse processes, the neural spine, both prezygapophyses and postzygapophyses and only the hyposphene is still mostly intact. Also, the right posterior ventral part of the centrum and the ventral part of the anterior facet are eroded and lightly damaged. The whole vertebra is slightly crushed mediolaterally and the left side is better preserved, a situation similar to that of the entire vertebral series (Fig S3).

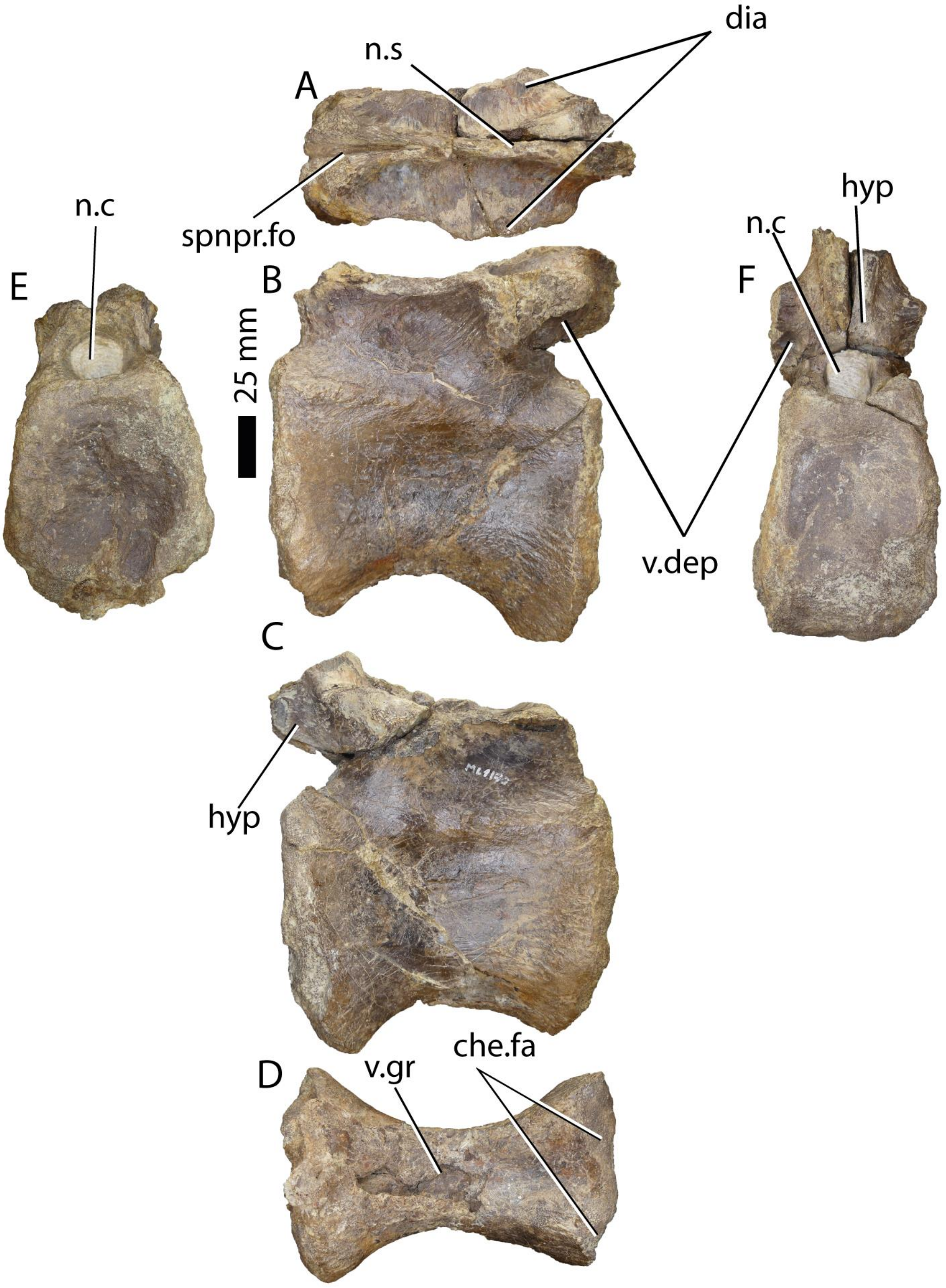

Fig S3. Caudal vertebra of *Iberospinus natarioi* (ML1190-16)

A, Dorsal, **B,C**, lateral, **D**, ventral, **E**, anterior and **F** posterior view.

n.c, neural canal, **v.dep**, ventral depression in the neural arch, **dia**, diaphysis, **n.s**, broken neural spine, **hyp**, hypantrum, **spnpr.fo**, spinoprezygapophyseal fossa, **v.gr**, ventral groove, **che.fa**, chevron facets.

The anterior facet of the centrum is clearly concave and oval, without any distinguishable cotile on it. The ventral margin of the neural canal in this anterior facet is mostly rectilinear mediolaterally.

The neurocentral suture is clearly visible in the anterior half of the vertebra, but it becomes less marked as it goes backwards, being almost invisible near the posterior rim of the facet. This suture also tapers ventrally as it runs along the centrum, in a similar manner to ML1190-15. Finally, there is a shallow depression in the middle of the lateral surface of the centrum, but not any signs of pleurocoels.

In ventral view the vertebra is clearly hourglass shaped and, with the ventral groove clearly marked in the posterior part. Both ridges form the base of the chevron facets.

The posterior facet of the centrum is relatively flat, although the dorsal half of it is more concave.

In the neural arch, the spinoprezygapophyseal laminae converge in an acute angle well in front of the transverse processes, delimiting a clear and elongated spinoprezygapophyseal fossa. The transverse processes are situated in the posterior part of the neural arch and have a small depression in their posteroventral side. The hyposphene protrudes posteriorly over the posterior facet of the centrum and is about 14 mm wide.

ML1190-22

This vertebra has not previously been described. It is strongly crushed mediolaterally, with the right ventral part of the centrum damaged. It still preserves the neural arch, although with just the eroded and damaged right prezygapophysis, all the other characters except the base of the transverse processes being lost or eroded (Fig S4).

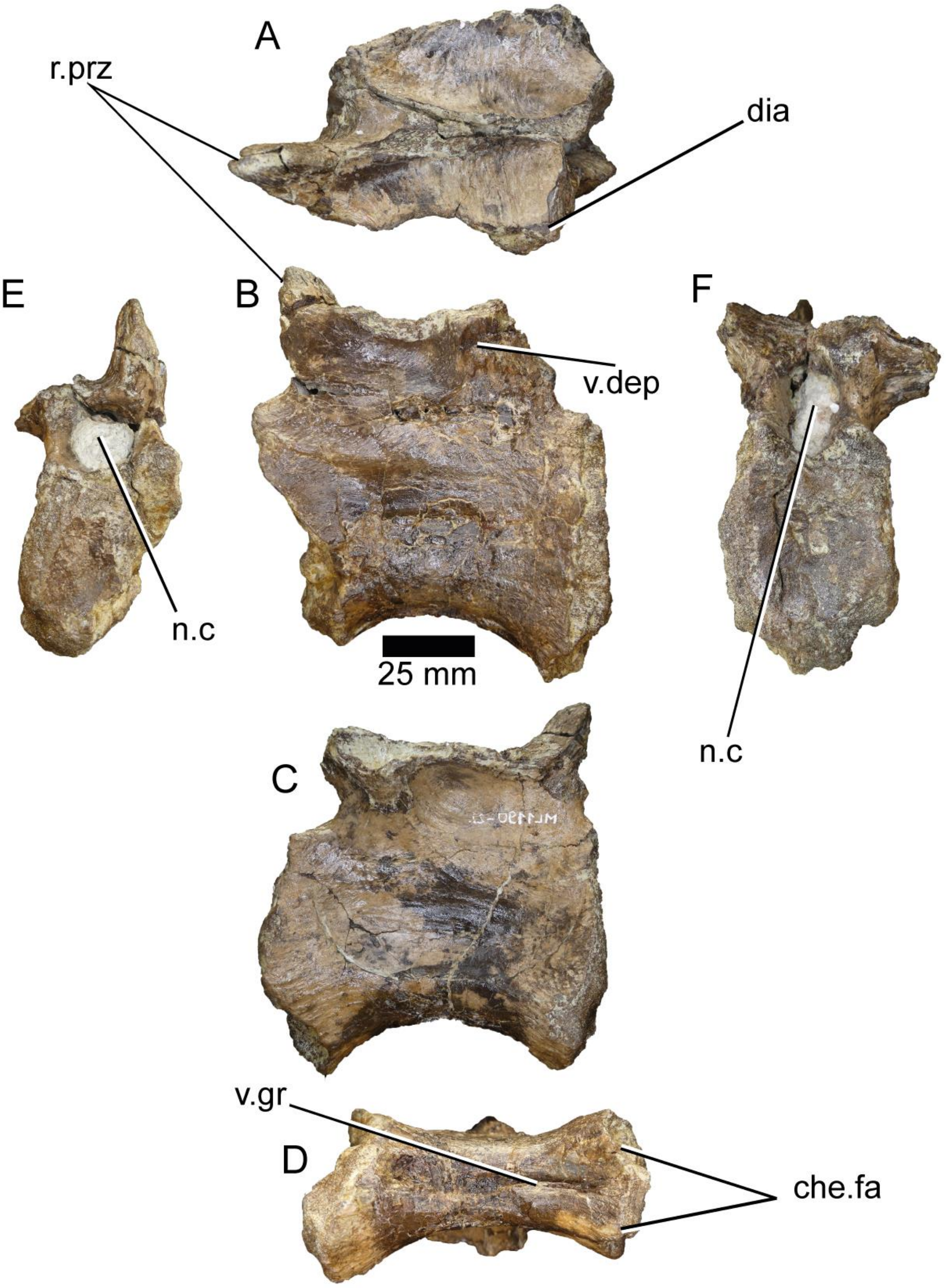

Fig S4. Caudal vertebra of *Iberospinus natarioi* (ML1190-22)

A, Dorsal, **B,C**, lateral, **D**, ventral, **E**, anterior and **F** posterior view.

n.c, neural canal, **v.dep**, ventral depression in the neural arch, **dia**, diaphysis, **v.gr**, ventral groove, **che.fa**, chevron facets, **prz**, remains of prezygapophysis.

The preserved part of the anterior facet of the centrum is markedly oval and concave. The neurocentral suture is almost invisible in the lateral surface of the centrum, being only possible to see in the anteriormost part of it.

The ventral groove runs across the midline of the vertebra, clearly marked, even in the anterior half, although the deformation of the whole vertebra might make it more pronounced. The right chevron facet is slightly depressed, although this could be distorted by the preservation. The preserved part of the posterior central facet is slightly concave.

In the neural arch, the right prezygapophysis elevates in an angle of about 70º from the dorsal margin of the centrum. The spinoprezygapophyseal laminae are not well preserved but the spinoprezygapophyseal fossa delimited by them is much shallower and less elongated anteroposteriorly than in ML1190-16. In the anteroventral part of the base of the transverse processes there is a shallow depression, of bigger size than the small depression situated in the posteroventral part of them (albeit this last one is shallower than in ML1190-16).

ML1190-18

This vertebra was figured and interpreted as a mid posterior caudal vertebra by (1). The centrum is eroded in the left anteroventral part and in the left posterior part. In its left side, the centrum bears an oval mark with the major axis pointing anterodorsally-posteroventrally; measuring 21 mm in length and 11mm in width. This mark was already noted by (1) and attributed to the perimortem damage provoked by a predator or scavenger. The neural arch of the vertebra is still in place and the bases of both transverse processes and the neural spine are in place, in spite of this last feature being slightly deformed. The pre- and post-zygapophyses are completely gone (Fig S5).

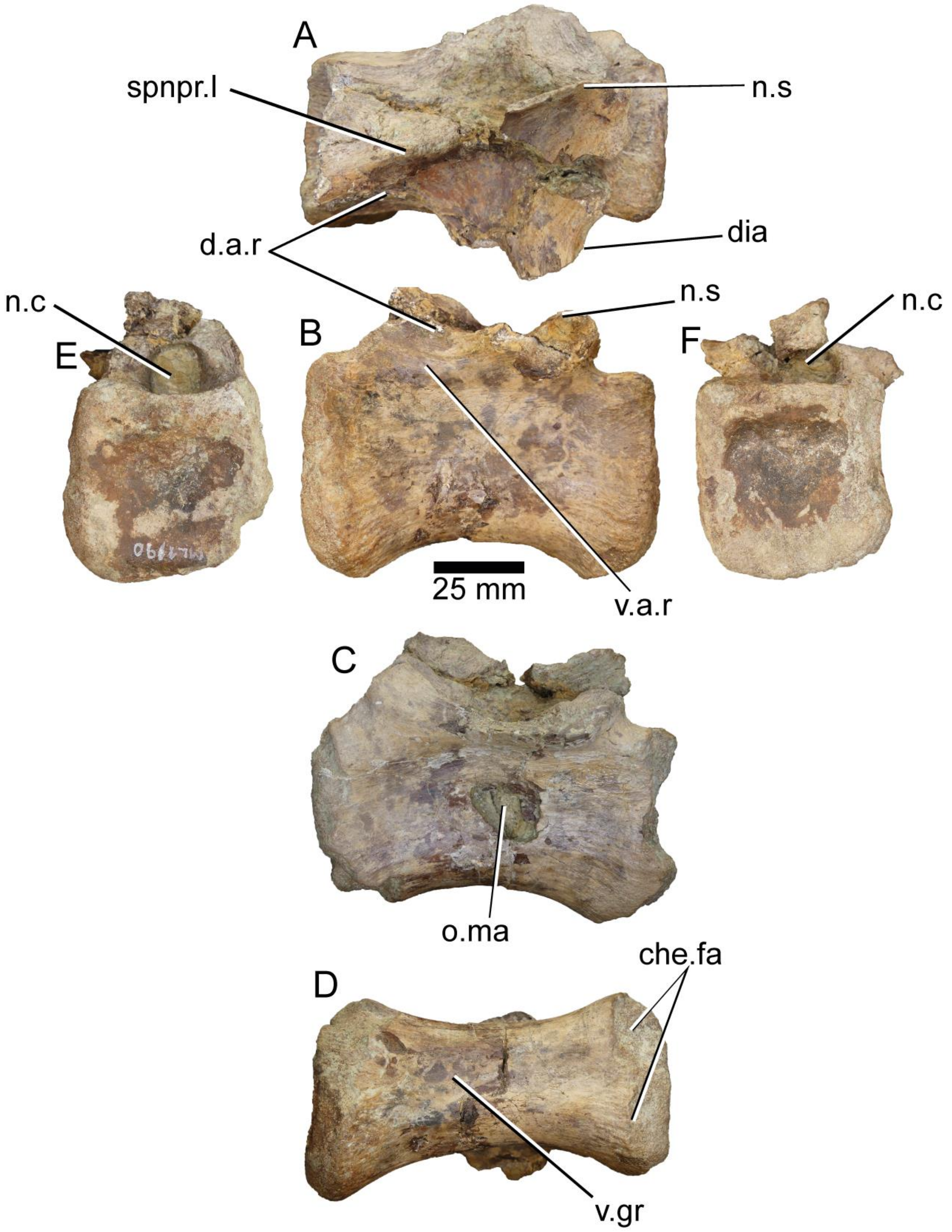

Fig S5. Caudal vertebra of *Iberospinus natarioi* (ML1190-18)

A, Dorsal, **B,C**, lateral, **D**, ventral, **E**, anterior and **F** posterior view.

n.c, neural canal, **v.a.r**, ventral anterior ridge, **d.a.r**, dorsal anterior ridge, **dia**, diaphysis, **n.s**, broken neural spine, **spnpr.l**, spinoprezygapophyseal lamina, **v.gr**, ventral groove, **che.fa**, chevron facets, **o.ma**, oval mark.

The anterior facet of the centrum is relatively square shaped, entirely concave. The ventral surface of the anteriormost part of the neural canal is slightly bent ventrally on its medial part.

The lateral surface of the centrum is relatively smooth, with some minor rugosities in the dorsal half. In the ventral view the vertebra is hour-glass shaped and its ventral surface presents a clear groove in the midline, although that is less marked than in previously described vertebrae.

The posterior facet of the centrum is even more square shaped than the anterior face, with the dorsal right part making an angle of almost 90º between the dorsal and right lateral rims. The facet is concave, but only clearly on its dorsal half, with the ventral part more flat.

In the anterior dorsal part of the neural arch it is possible to see a fragment of the right spinoprezygapophyseal lamina, as well as a portion of the floor of the spinoprezygapophyseal fossa. There are two ridges running anteriorly from the anterior part of the base of the transverse processes. The ventral one finishes in the lateral wall of the neural canal opening, the dorsal one goes towards the spinoprezygapophyseal lamina, both ridges delimiting a fossa anterior to the transverse processes. The posterior part of the neural spine is visible, but not much information can be taken from it.

ML1190-21

This vertebra is unpublished. It is the most damaged one that could be recognized in the entire series. Only the postero-ventral left portion of it has part of the bone surface intact (Fig S6).

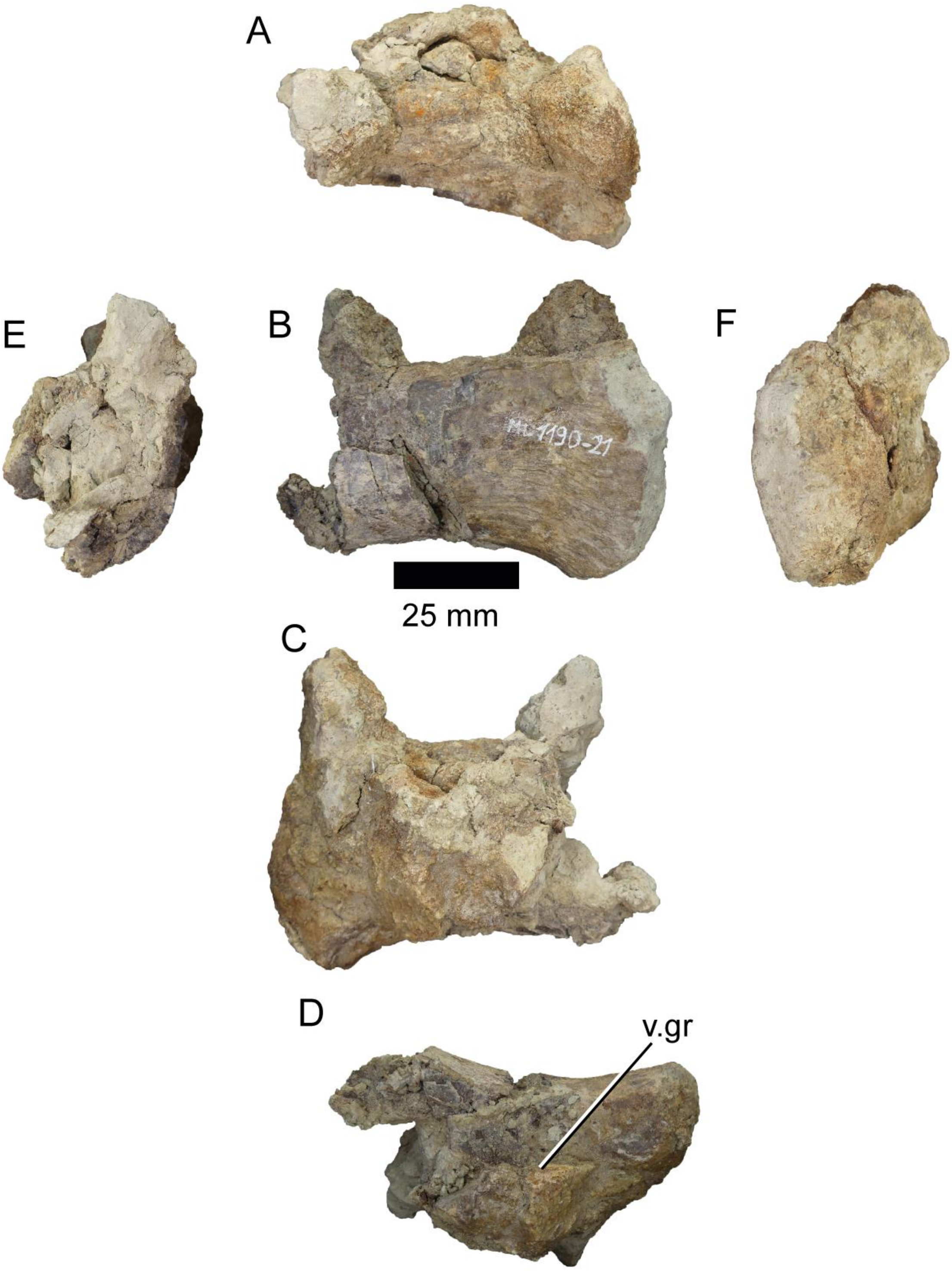

Fig S6. Partial caudal vertebra of *Iberospinus natarioi* (ML1190-21)

A, Dorsal, **B,C**, lateral, **D**, ventral, **E**, anterior and **F** posterior view.

v.gr, ventral groove.

The part that remains of the lateral surface of the vertebra has the characteristic rugosities towards the posterior part.

In the ventral surface the groove that runs across the midline is still distinguishable because there is part of the right ridge that delimited it still in place.

The posterior facet of the centrum is mainly completely eroded away, but the general pattern of the other vertebras can be recognized, with a ventral part more flattened meanwhile the part that remains of the dorsal half has a shape congruent with a more concave surface.

ML1190-20

This vertebra was already mentioned by (1). It comprises just the posterior half of a centrum, obliquely broken towards the posterior right part and missing the neural arch completely. The posterior right part is also eroded, including the small lateral surface visible (Fig S7).

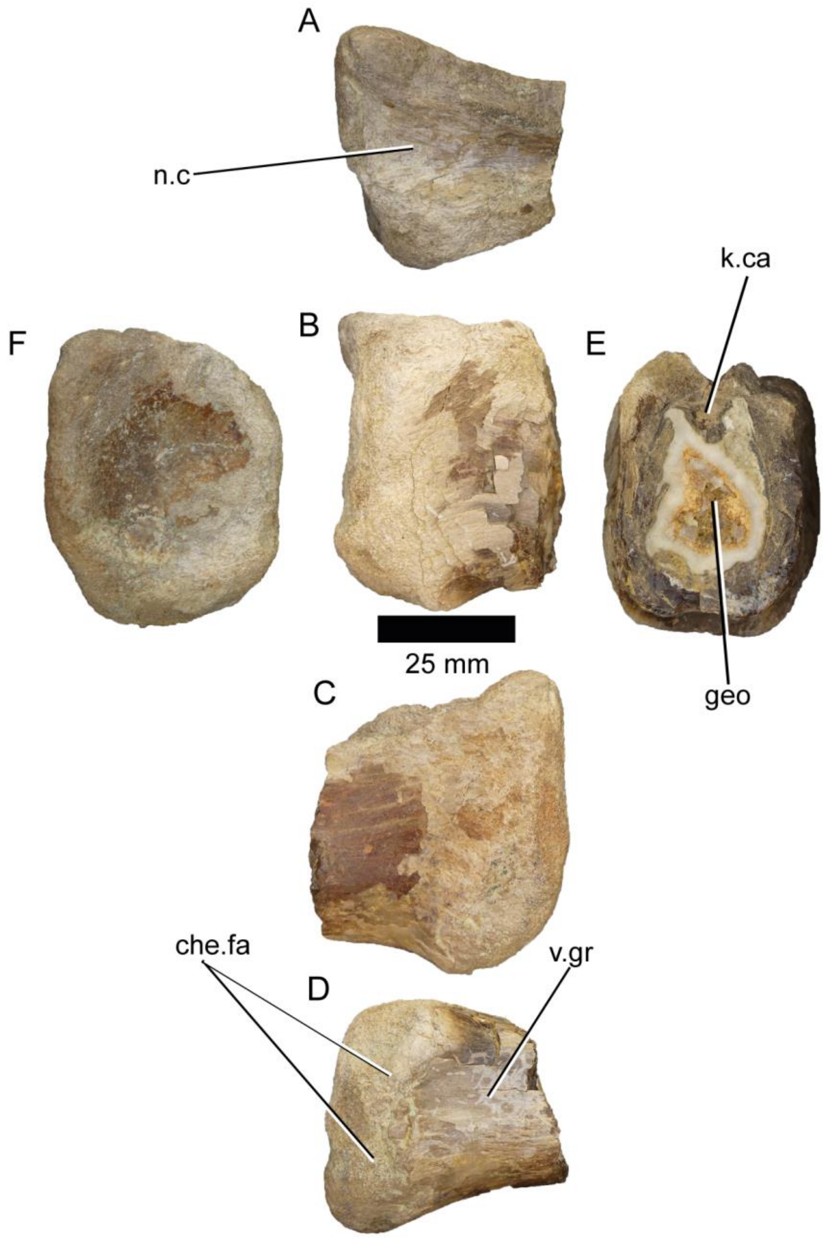

Fig S7. Caudal vertebra of *Iberospinus natarioi* (ML1190-20)

A, Dorsal, **B,C**, lateral, **D**, ventral, **E**, anterior and **F** posterior view.

n.c, neural canal, **v.gr**, ventral groove, **che.fa**, chevron facets, **geo**, geode structure, **k.ca**, keel in the dorsal surface of the vertebral cavity.

The broken surface in the centrum shows the hollow interior of the vertebra, filled with a geode structure already noted by (1). This cross section shows a morphology that reflects that of the surface of the centrum, with a keel pointing ventrally from the dorsal surface of the cavity, in conformity with the depth that the neural canal reaches into the centrum.

Dorsally, the neural canal floor poses longitudinal rugosities similar to those seen in other vertebras, meanwhile the rugosities along the neurocentral suture´s surfaces are less marked than in ML1190-15. The lateral surface of the centrum is relatively featureless. The ventral part still possesses clearly the ventral groove seen in other vertebras, whose lateral ridges finish posteriorly in the chevron facets. In the posterior facet of the centrum, only the dorsal left corner is still mostly intact, forming a slightly acute angle between the dorsal and left lateral rims of it.

The surface of the facet is slightly concave in the dorsal half; meanwhile the ventral half is mostly flat.

ML1190-26

This unpublished vertebra is one of the best preserved of the specimen ML1190. The centrum is well preserved except for the anterior and posterior right lateral rims, which are slightly eroded. The neural arch is still in place, but is better preserved in the anterior part, with both prezygapophyses still in place as well as the base of neural spine and the bases of both transverse processes. The posterior part is worst preserved with the roof of the neural canal eroded as well as both postzygapophyses gone (Fig S8).

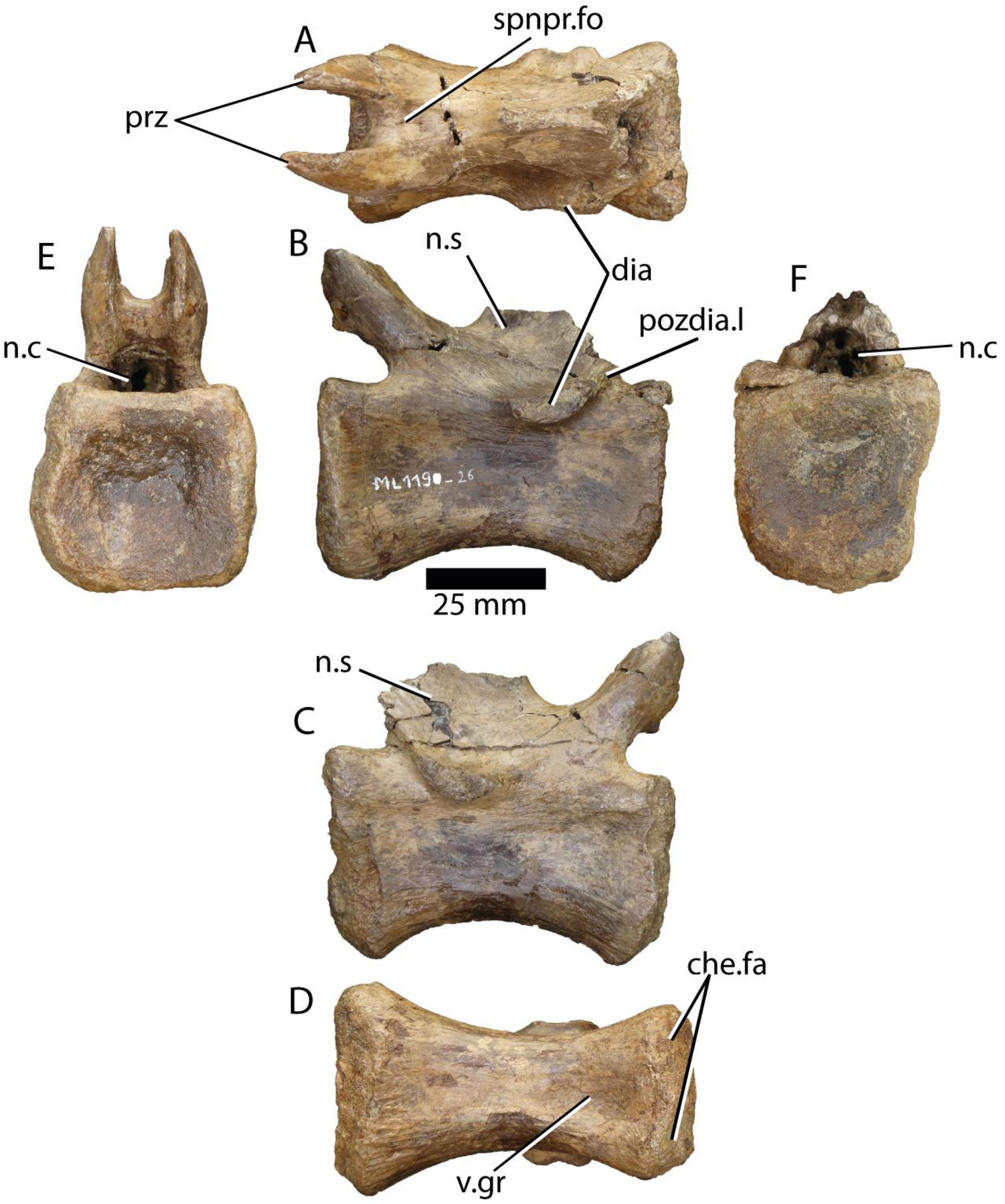

Fig S8. Caudal vertebra of *Iberospinus natarioi* (ML1190-26)

A, Dorsal, **B,C**, lateral, **D**, ventral, **E**, anterior and **F** posterior view.

n.c, neural canal, **v.gr**, ventral groove, **che.fa**, chevron facets, **prz**, prezygapophyses, **spnpr.fo**, spinoprezygapophyseal fossa, **dia**, diaphysis, **pozdia.l**, postzygapodiapophyseal laminae, **dia**, diaphysis, **pozdia.l**, keel in the dorsal surface of the vertebral cavity, **n.s**, broken neural spine.

The anterior facet of the centrum is concave as with other vertebras, the lateral rims tilt medially slightly towards the dorsal part. The lateral surface poses a slightly shallow area in its anterodorsal part below the base of the prezygapophyses. The ventral surface possesses a ventral groove, although it is relatively shallow, with the ridges that delimit it finishing in the base of the chevron facets. Also in this surface there are small ridges running ateroposteriorlly in the first 10 mm posterior to the rim of the anterior facet. The posterior facet has a concave dorsal half and a flat ventral half.

The prezygapophyses of the neural arch protrude anteriorly over the anterior facet of the centrum in dorsal view. They project anterodorsally in an angle of ~70º with the centrum and are about 30 mm long from the base (20 mm from the anterior end of the spinoprezygapophyseal fossa), with a dorsoventral diameter of about 18 mm. Their facets are pointing medially and between them there is a distance of 11mm. The spinoprezygapophyseal laminae are not well developed, and they converge in the anteriormost point of the base of the neural spine, delimiting an extremely shallow spinoprezygapophyseal fossa. The anterior end of the neural spine is 8 mm wide and is situated only slightly anteriorly of the anteroposterior middle point of the vertebra. The diapophyses bases are situated, posteriorly to the aforementioned point and they poses a ridge running posterodorsally towards the point where the bases of the postzygapophyses would be (postzygapophyseal laminae), in an angle with the centrum similar of that formed by the prezygapophyses.

ML1190-23

This vertebra is unpublished and the neural arch is badly damaged. The centrum itself is well preserved (Fig S9).

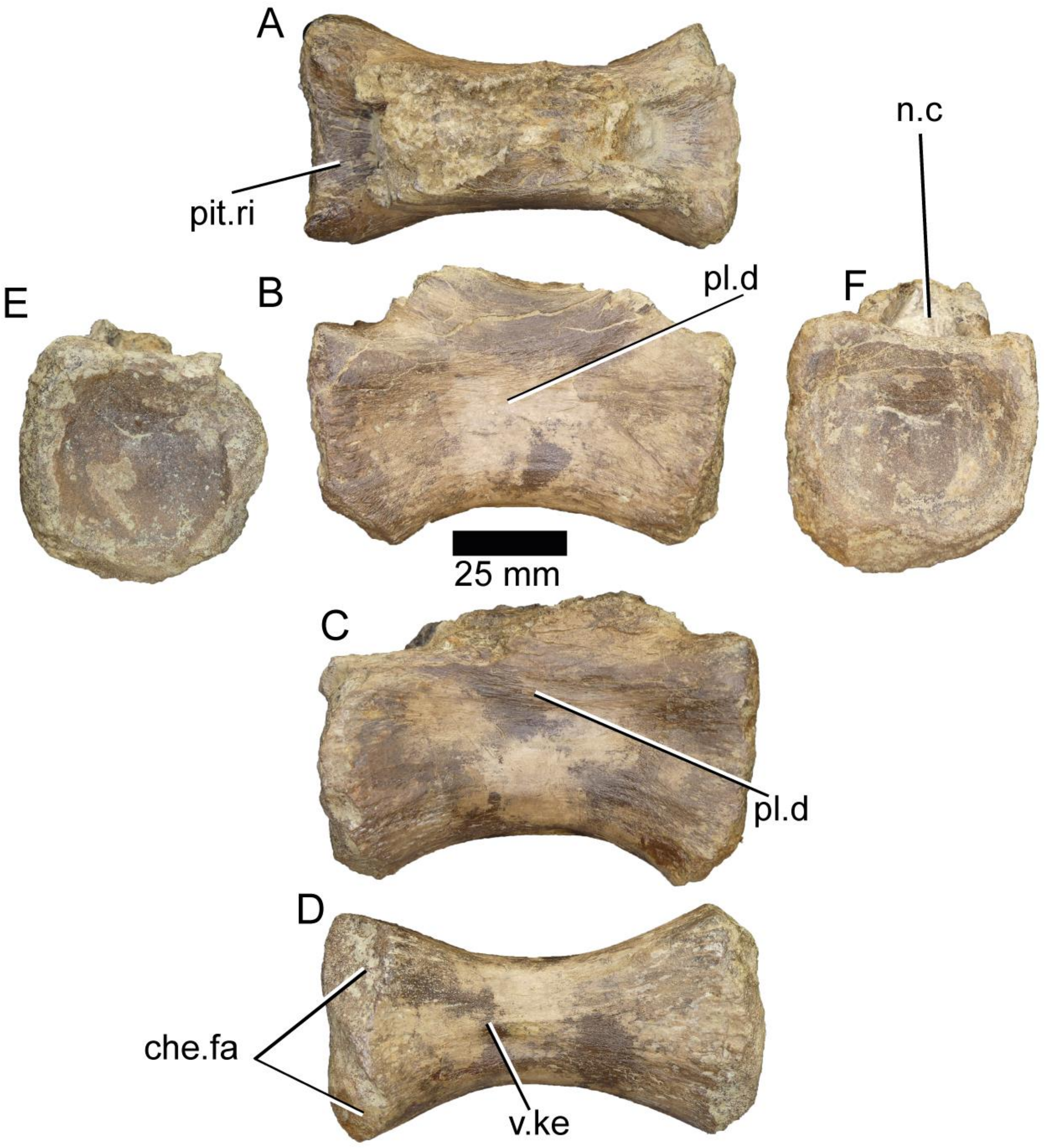

Fig S9. Caudal vertebra of *Iberospinus natarioi* (ML1190-23)

A, Dorsal, **B,C**, lateral, **D**, ventral, **E**, anterior and **F** posterior view.

n.c, neural canal, **v.ke**, ventral keel, **che.fa**, chevron facets, **pl.d**, pleurocelic depression, **pit.ri**, pits and ridges in the floor of the neural canal.

The exposed floor of the neural canal presents a series of ridges and pits. The anterior facet of the centrum is concave; with the lateral rims slightly flexing outward, although the overall

shape of the facet is square. In the dorsal half of the lateral surface of the centrum there is a shallow fossa across much of the lateral surface of the centrum (pleurocelic depression). The ventral surface is mostly smooth although there is an extremely thin keel of about 25 mm long situated slightly posteriorly in the surface. The posterior facet has clearly 90º corners between the upper and lateral rims of the posterior surface, being the overall shape rectangular, taller than wider. The chevron facets seem to be asymmetrical, probably due to a certain degree of deformation or erosion, being the right one larger and more anteroposteriorly oriented.

ML1190-19

This vertebra was already mentioned in (1). It is the best preserved vertebra of the entire series. The centrum is completely preserved, with almost no erosion in the rims of the facets and the original surface mostly intact. The neural arch is more damaged, with cracks over most of its surface. The left prezygapophysis anterior part is eroded; there is only a hint of the anterior process of the neural spine and its posterior part, as well as the postzygapophysis are damaged (Fig S10).

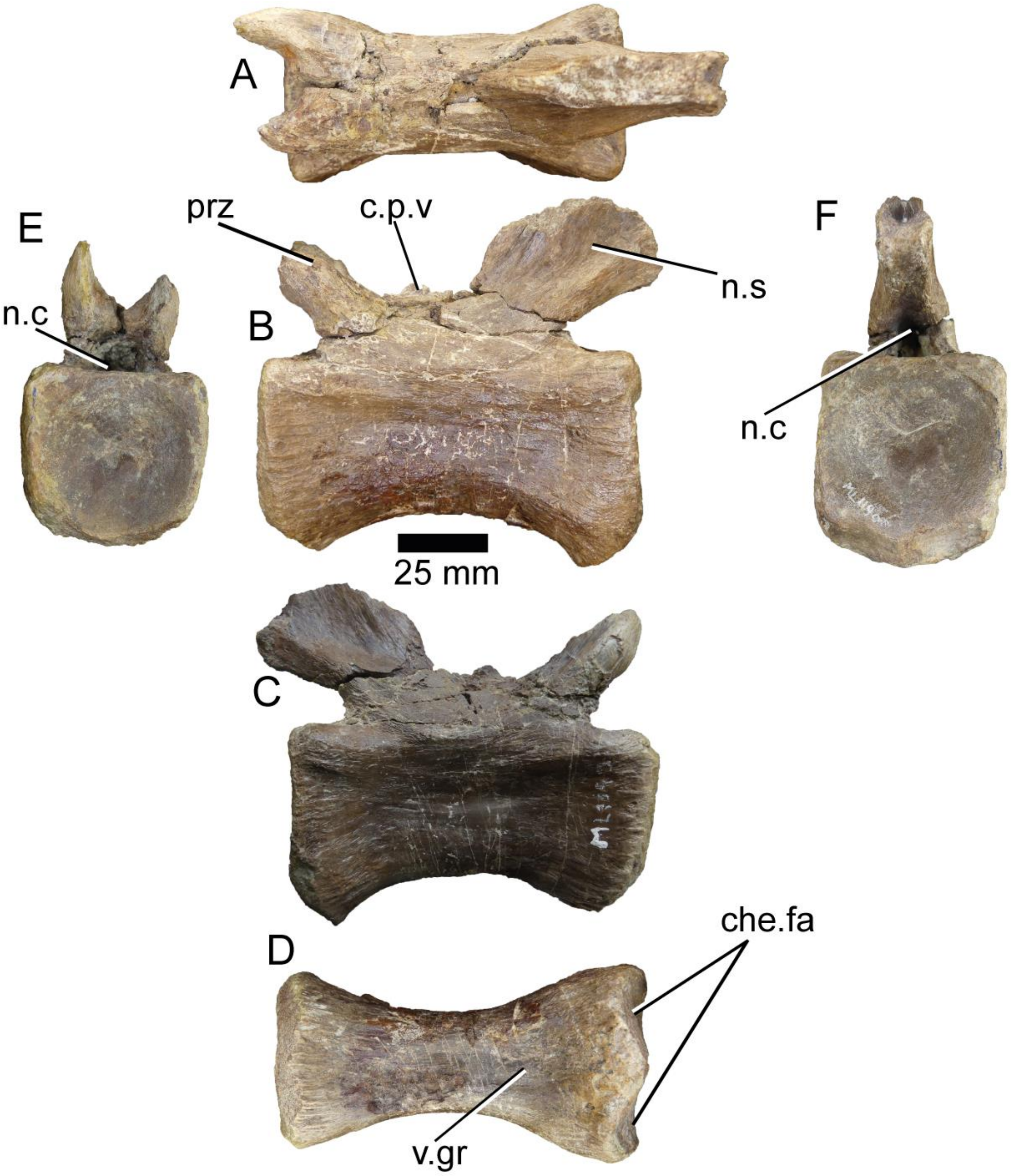

Fig S10. Caudal vertebra of *Iberospinus natarioi* (ML1190-19)

A, Dorsal, **B,C**, lateral, **D**, ventral, **E**, anterior and **F** posterior view.

prz, prezygapophysis, **c.p.v**, cranial process of the vertebra, **che.fa**, chevron facets, **n.s**, neural spine, **v.gr**, ventral groove, **n.c**, neural canal.

The anterior facet of the centrum is markedly concave, being also square in shape, mostly in the dorsal part, where its dorsal rim is completely straight and forms 90º angles with the lateral rims. The ventral rim is more oval, with two surfaces (not as marked as the chevron facets) to accommodate the chevron situated anteriorly, that are about 13 mm wide dorsoventrally. There is a hint of a cotile in this surface of about 7 mm of diameter, although its perimeter is irregular.

The lateral surfaces of the centrum present smooth protuberances of about 9 mm dorsoventrally and 20 mm anteropostertiorly, both in the dorsal anterior and dorsal posterior parts. Otherwise the surfaces are smooth.

The ventral surface presents a shallow groove anteroposteriorly, with the ridges delimiting it relatively smooth. The anterior part of the ventral surface presents a series of anteroposterior small ridges, similar to those of ML1190-26, of about 10 mm long.

The posterior centrum facet is also concave in shape, taller dorsoventrally than the anterior one. The dorsal rim of the facet is equal to the anterior one, forming 90º angles with the lateral rims. The chevron facets, situated where the ventral ridges finish, are about 17 mm dorsoventrally.

The prezygapophyses form an angle of ~60º with the centrum, slightly overhanging in dorsal view the anterior facet of it. They are separated for about 15 mm, although the distance is slightly distorted by the reconstruction. They are also 15 mm tall dorsoventrally and project another 15 mm anterodorsally from the neural arch. It is not possible to observe if there are spinoprezygapophyseal laminae or fossa because of the conservation, but there is a small protuberance slightly situated in the anterior part of the vertebra, 6 mm tall and 5 mm wide herein interpreted as the cranial process of the vertebra also mentioned in a spinosaur from thailand (2). There are no transverse processes in this vertebra, but there are two smooth ridges only 2 or 3 mm wide, slightly anterior to the anteroposterior midpoint of the vertebra that could be homologous to the transverse processes preserved in more anterior vertebrae.

Therefore the smooth ridge that runs from their posterior part towards the postzygapophyses would be homologous to the diapostzygapophyseal lamina. The neural spine is about 14 mm wide in the base and is broken ~40 mm above the centrum. There is a triangular canal exposed by this breakage running posteroventrally across it, 9 mm tall and 6 mm wide in the ventral surface. Only the anteriormost part of the postzygapophyses is conserved. They overhang the posterior surface of the centrum in dorsal view and are separated ventrally for about 5 mm at their base.

ML1190-27

This vertebra is unpublished. It is relatively well preserved, although the right lateral rim of the anterior face of the centrum is heavily eroded. The neural arch is still in place, although the pre- and post- zygapophyses (as well as the neural spine) are totally eroded away (Fig S11).

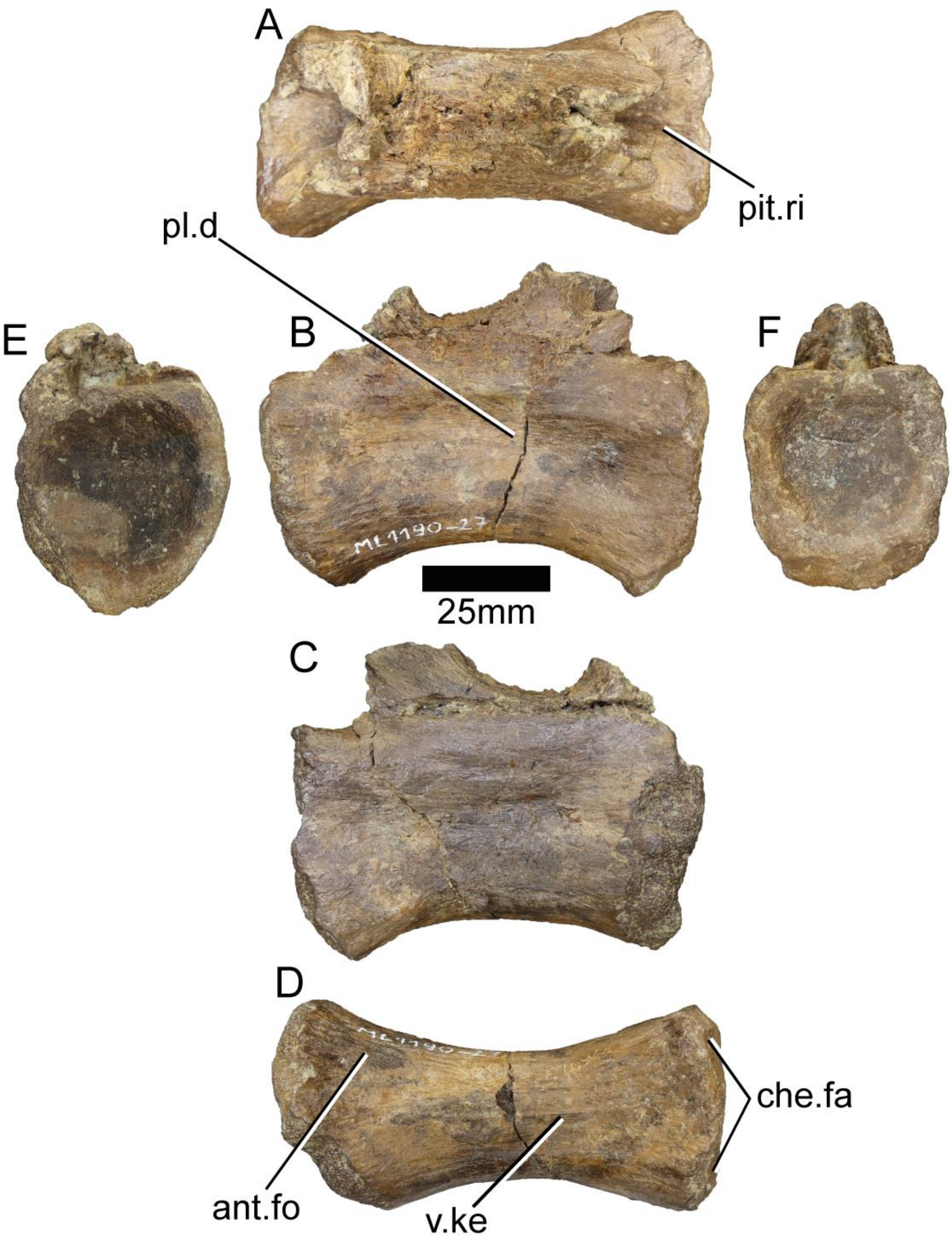

Fig S11. Caudal vertebra of *Iberospinus natarioi* (ML1190-27)

A, Dorsal, **B,C**, lateral, **D**, ventral, **E**, anterior and **F** posterior view.

ant.fo, anterior foramen, **v.ke**, ventral keel, **che.fa**, chevron facets, **pl.d**, pleurocelic depression, **pit.ri**, pits and ridges in the floor of the neural canal.

The anterior face of the centrum is concave and the lateral rim that remains is clearly bowed toward the lateral side. The lateral surface has a shallow pleurocentral depression, crossed by some longitudinal ridges. The ventral surface has a smooth and small keel across the midline; there are also some ridges and a small foramen in the anterior left ventral part. The posterior face of the centrum is also concave, the upper rim of it is mostly straight, however, the lateral rims converge slightly towards the ventral part and are slightly bowed, making this face taller than wider. The chevron facets are 13 cm high dorsoventraly.

The neural arch has lost all of its major features, but it is still possible to say that the vertebra had no transverse processes at all. There is a shallow ridge situated medially in the floor of the ventral canal posteriorly.

ML1190-25

This vertebra is unpublished and it is the smallest in overall dimensions of the sample. The right anterior part of the centrum is damaged, with most of the anterior facet gone too. The neural arch is missing. There is some minor erosion in the posterior facet (Fig S12).

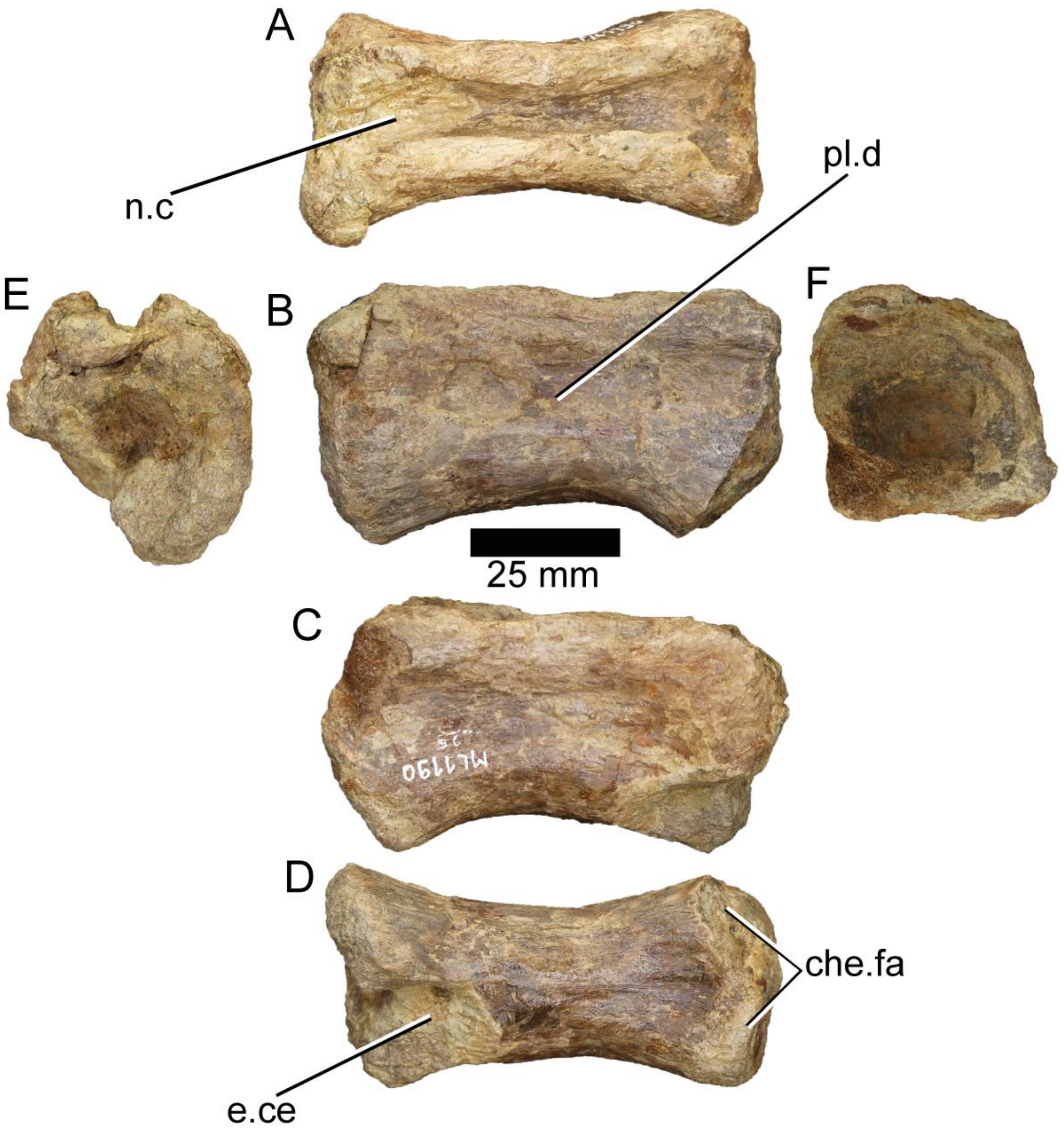

Fig S12. Caudal vertebra of *Iberospinus natarioi* (ML1190-25)

A, Dorsal, **B,C**, lateral, **D**, ventral, **E**, anterior and **F** posterior view.

e.ce, eroded part of the centrum, **n.c**, floor of the neural canal, **che.fa**, chevron facets, **pl.d**, pleurocelic depression.

By the remaining surface of the anterior face it is possible to say that it was concave in shape, although not much about the rim of the face can be said because of the erosion. It is possible to see the hollow interior of the vertebra by a small fracture in the damaged area.

The dorsal part of the centrum bears the floor of the neural canal, which is deeply excavated into the centrum. Opposed to what happens in ML1190-15 the canal in its central part is mostly regular (6 mm wide), as well as equally deep. There are no clear ridges running anteroposteriorly on it.

The left anterior lateral surface presents a small depression, not morphological but either pathological or taphonomical. Its shape is trapezoidal, with the long sides pointing anterodorsally to posteroventrally. Its length is 13 mm and the width is 9 mm. In the right side of the centrum, slightly more posterior, but still in the anterior part of the centrum there is a small bump of a similar nature, with the long axis located precisely anteroposteriorly. It is 10 mm long and 5 mm wide. Overall both lateral surfaces present a pleurocentral depression.

The ventral surface does not present any ridge or keel on in, just the buttresses of the chevron facets that project anteriorly for about 17 mm.

The posterior facet of the centrum is slightly eroded dorsally and it is concave in shape. The dorsal and lateral rims of the facet are eroded, but it is possible to say that it was nearly square shaped.

ML1190-240

This caudal vertebra was recovered during the excavation of June 2020 and is one of the best preserved in the sample. The centrum is complete and mostly intact, as well the prezygapophyses and the neural arch. Most of the postzygapophyses and the first centimeters of the neural spine are reasonably complete too, despite the damages caused by the gypsum that cuts part of the neural arch (Fig S12).

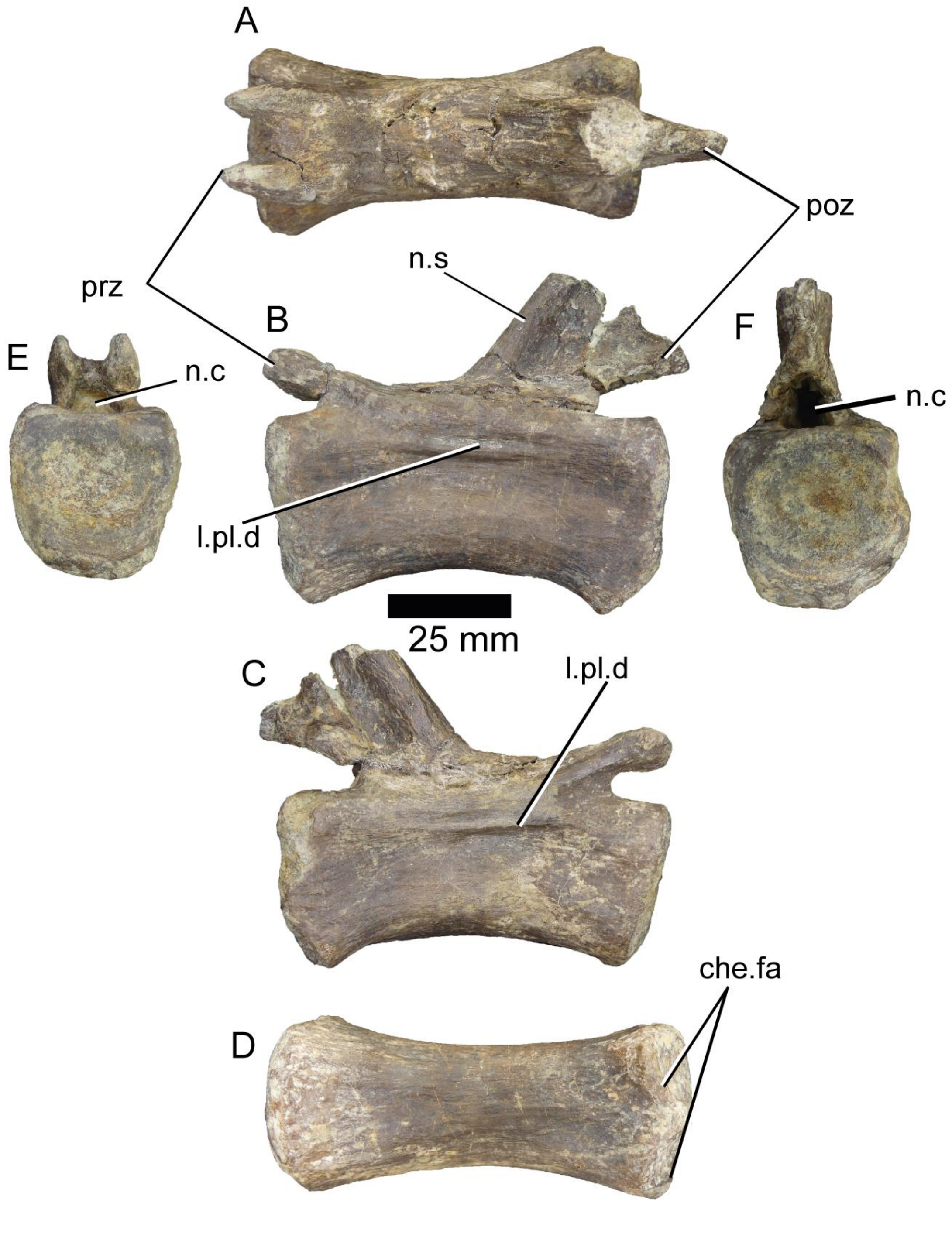

Fig S13. Caudal vertebra of *Iberospinus natarioi* (ML1190-240)

A, Dorsal, **B,C**, lateral, **D**, ventral, **E**, anterior and **F** posterior view.

prz, prezygapophysis, **poz**, postzygapophysis, **che.fa**, chevron facets, **l.pl.d**, lamina in pleurocelic depression, **n.s**, neural spine, **n.c**, neural canal.

The anterior facet of the centrum is trapezoid in shape, with the bigger side dorsally oriented, clearly concave with wide rims around the concavity, which is reniform in shape. The floor of the anterior neural canal is mostly straight, with a slight concavity looking from anterior view. The lateral surfaces of the centrum present relatively deep and anteroposteriorly elongated concavities, about 30 mm anteroposteriorly in length and 11 mm wide dorsoventrally. They present a ridge in their dorsal half that extends anteroposteriorly across their length and is about 3 mm dorsoventrally thick. The ventral surface of the centrum is mostly flat with a small ridge, centered in its posterior half. The posterior facet of the centrum is square shaped and concave except for the chevron facets that are about 15 mm in length.

The prezygapophyses have their articular surfaces almost perfectly in 90º angle respect to the centrum in anterior view. They are separated by 9 mm, 8 mm dorsoventrally wide and project 6 mm anteriorly from the neural arch in a sharp angle of less than 30º in lateral view, slightly overhanging the centrum. Posterior to them in the neural arch, there are no signs of anterior process of the neural spine or spinoprezygapophyseal fossa, just a mostly flat surface. The base of the neural spine is 13 mm wide mediolaterally. It presents two ridges running dorsally along its length from each side of its anterior margin, separated by 6 mm and 8 mm wide anteroposteriorly. A thin lamina, 4 mm thick connects the posterior margin of the spine with the fused postzygapophyses, whose articular surfaces are perpendicular to the centrum in posterior view. They project 22 mm posteriorly to the end of the neural canal and overhang by several mm the posterior facet of the centrum.

ML1190-241

This caudal vertebra was recovered during the excavation of June 2020. The right prezygapophysis is eroded away and the right side of the neural arch is mostly gone, being attached thanks to sediment. The left side (in posterior view) is much better preserved with just minor cracks on it. The postzygapophyses are highly damaged, but still recognizable (Fig S14).

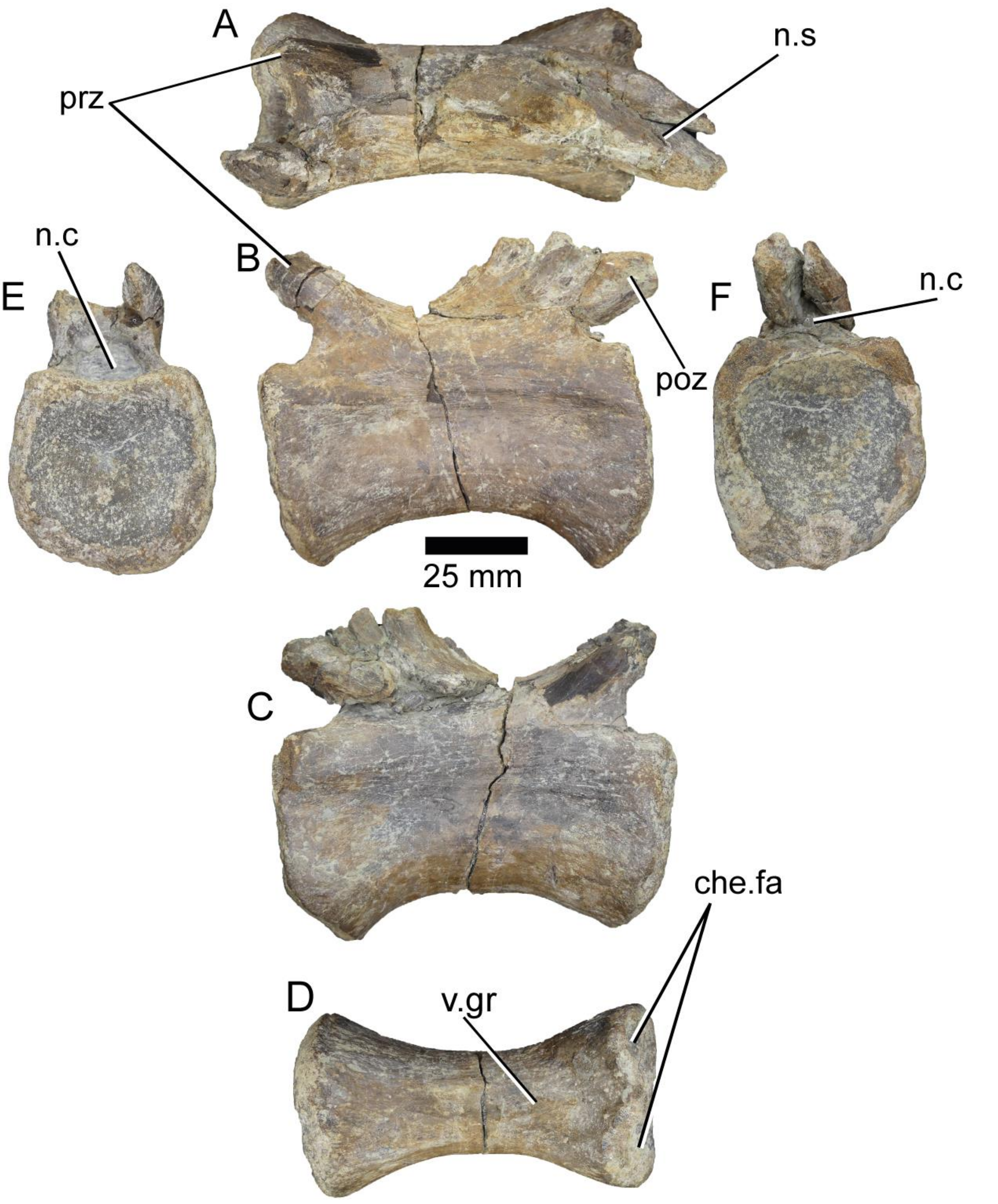

Fig S14. Caudal vertebra of *Iberospinus natarioi* (ML1190-241)

A, Dorsal, **B,C**, lateral, **D**, ventral, **E**, anterior and **F** posterior view.

prz, prezygapophysis, **poz**, postzygapophysis, **che.fa**, chevron facets, **n.s**, deformed base of the neural spine, **v.gr**, ventral groove, **n.c**, neural canal.

The anterior half of the vertebra is square in anterior view and concave, with the deepest part in the dorsal half of the facet. The lateral surfaces of the centrum do not show any pleurocels or concavities. The ventral surface has a shallow groove whose ridges finish at the chevron facets, it is about 11 mm wide. The posterior surface is rectangular in posterior view, with the deepest point in the upper quarter of the centrum facet. The chevron facets are about 18 mm long.

The prezygapophyses are separated by about 10 mm, do not overhang the centrum and project in a 30 degree angle from the neural arch in lateral view with the centrum. The articular facet of the preserved right prezygapophysis is 14 mm wide dorsoventrally and projects about 12 mm in front of the neural arch. There is an extremely thin lamina medially running across the dorsal surface of the neural arch from the base of the neural spine to the point of connection of the prezygapophyses. The neural spine is about 13 mm wide in its anterior part, where it emerges from the neural arch. The postzygapophyses slightly overhang the posterior centrum facet.

ML1190-275

This half caudal vertebral centrum was discovered in June of 2020. Given the lack of chevron facets it is interpreted as an anterior part of the centrum. Interestingly it does not fit with ML1190-20, being smaller in size. The anterior facet is mostly square shaped and concave. The floor of the neural canal presents some eroded ridges and pits. During all the preserved length it diminishes its mediolateral width, reaching 4 mm at the point where the centrum is broken. The lateral surfaces are flat and the ventral surface presents the hint of two ridges delimiting a groove, about 7 mm wide. The broken surface of the centrum is hollow, but does nor present a mirror image of the neural canal as in ML1190-20, instead the cavity is oval in shape, with the long axis dorsoventrally oriented. The walls of the vertebra around this cavity are around 7 mm thick (Fig S15).

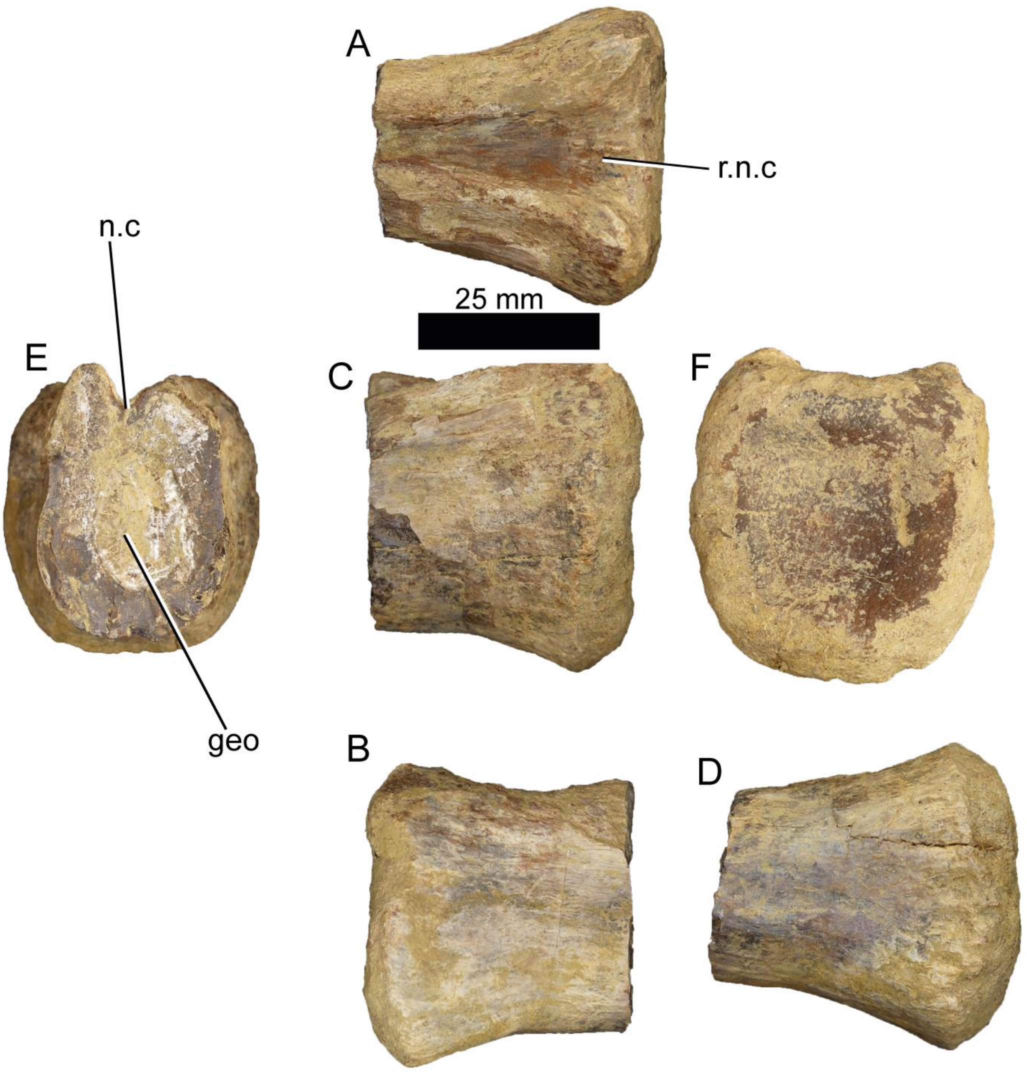

Fig S15. Caudal vertebra of *Iberospinus natarioi* (ML1190-275)

A, Dorsal, **B,C**, lateral, **D**, ventral, **E**, anterior and **F** posterior view.

r.n.c, ridges in the floor of the neural canal, **n.c**, neural canal, **geo**, geode structure.

References

1. Mateus O, Araújo R, Natário C, Castanhinha R. A new specimen of the theropod dinosaur Baryonyx from the early Cretaceous of Portugal and taxonomic validity of Suchosaurus. Zootaxa. 2011 Apr 21;2827(1):54–68.

2. Samathi A, Sander PM, Chanthasit P. A spinosaurid from Thailand (Sao Khua Formation, Early Cretaceous) and a reassessment of Camarillasaurus cirugedae from the Early Cretaceous of Spain. Historical Biology. 2021 Feb 8;0(0):1–15.
